# Nonlinear quantum light source with van der Waals ferroelectric NbOX_2_ (X = Br, I)

**DOI:** 10.1038/s41467-026-75018-4

**Published:** 2026-06-30

**Authors:** Yanru Wang, Yunkun Wu, Yong Liu, Wanfu Shen, Ya Feng, Yue Liu, Chen Yi, Kun He, Miaomiao Liu, Ping Lu, Shaojun Liu, Yingying Liu, Jingmei Tang, Liqiang Zhang, Chunguang Hu, Shula Chen, Guangcan Guo, Xifeng Ren, Xidong Duan

**Affiliations:** 1 Hunan Key Laboratory of Two-Dimensional Materials State Key Laboratory for Chemo/Biosensing and Chemometrics College of Chemistry and Chemical Engineering Hunan University, Changsha, China; 2 Laboratory of Quantum Information, University of Science and Technology of China, Hefei, China; 3 CAS Center for Excellence in Quantum Information and Quantum Physics, University of Science and Technology of China, Hefei, China; 4 Hefei National Laboratory, University of Science and Technology of China, Hefei, China; 5 Hunan Institute of Optoelectronic Integration and Key Laboratory for MicroNano Physics and Technology of Hunan Province, Hunan University, Changsha, China; 6 College of Materials Science and Engineering, Hunan University, Changsha, China; 7 State Key Laboratory of Precision Measuring Technology and Instruments, Tianjin University, Weijin Road 92, Nankai District, Tianjin, China; 8 Hunan Provincial Key Laboratory of Two-Dimensional Materials, School of Physics and Electronics, College of Semiconductors (College of Integrated Circuits), Hunan University, Changsha, China

**Keywords:** Nonlinear optics, Two-dimensional materials

## Abstract

Two-dimensional (2D) materials have been demonstrated as promising candidates for ultracompact entangled photon sources and on-chip integrated quantum photonic devices. Ferroelectric van der Waals materials have recently emerged as promising ultrathin sources of entangled photons via nonlinear optical processes. Here, the authors compare spontaneous parametric down conversion in NbOBr_2_ and NbOI_2_, demonstrating near infrared polarization entangled photon generation and linking performance to crystal structure and optical properties. Our results extend the wavelength range of NbOX_2_-based SPDC sources and demonstrate NbOI_2_ is especially suitable for near-infrared SPDC. Polarization entangled state (concurrence of 0.90 ± 0.05) can also be directly generated from NbOI_2_. Besides adding new candidates to the SPDC material library, our work provides new insight for the future generation of integrated quantum materials.

## Abstract (web summary)

Ferroelectric van der Waals materials have recently emerged as promising ultrathin sources of entangled photons via nonlinear optical processes. Here, the authors compare spontaneous parametric down-conversion in NbOBr_2_ and NbOI_2_, demonstrating near-infrared polarization-entangled photon generation and linking performance to crystal structure and optical properties.

## Introduction

Entangled photons are fundamental to the realize various quantum technologies, including quantum communication, quantum calculation, quantum imaging and quantum metrology, etc^1–5^. Spontaneous parametric down-conversion (SPDC), a second-order nonlinear optical process in which a high-energy pump photon splits into two lower-energy photons, serves as the most prevalent source for generating such entangled quantum light states. Conventionally, SPDC-based quantum light sources are typically enabled by second-order nonlinear optical (NLO) bulk crystals such as bulk beta barium borate^6,7^, potassium titanyl phosphate^8^and lithium niobate(LiNbO_3_)^9,10^. However, as the forefront field of quantum technology is advancing toward miniaturization and integration, these bulky sources, often exhibiting three-dimensional (3D) covalent bonding and relatively weak nonlinearities, impede their integration into compact integrated quantum systems. To circumvent these limitations, researchers have developed and demonstrated several new techniques for the on-chip SPDC quantum sources, including nonlinear waveguides such as periodically poled lithium niobate (PPLN)^11,12^; thin-film lithium niobate (TFLN)^13,14^, layer-poled lithium niobate (LPLN)^15^, and periodically poled potassium titanyl phosphate (PPKTP)^16^, as well as integration of nonlinear artificial photonic structures to enhance the light-matter interaction, such as aluminum nitride (AlN) microring^17^ and lithium niobate (LN) microdisk^18^. However, these approaches continue to depend on conventional nonlinear materials with dimensions typically in the tens of micrometers range and exhibiting moderate effective coefficients. Furthermore, the intricate fabrication process raises manufacturing costs and hinders large-scale batch fabrication. And their integration onto arbitrary substrates relies on epitaxial growth or wafer bonding techniques, which require strict lattice matching, severely limiting heterogeneous integration with silicon photonics or fiber optics. Thus, new materials with stronger nonlinear response need to be sought to further reduce the size of the quantum light source. Besides, thin-film based SPDC source exhibits different properties from phase-matched light sources due to the extremely small interaction length resulting from the reduction in size. The relaxation of phase-matching conditions broadens the frequency spectrum for entangled photon pairs, offering potential applications in quantum precision measurement^19^.

Materials always have three degrees of freedom in space; however, one degree of freedom in two-dimensional materials is always at the atomic thickness scale, which can be negligible, and two degrees of freedom are macroscopical or near macroscopical, including 2D layered materials and 2D non-layered materials. Two-dimensional layered materials refer to crystalline materials composed of a single atomic layer or several atomic layers, with strong covalent bonds within the layers and weak van der Waals (vdW) forces between adjacent layers. Their inherent thinness and the compatibility with substrate integration without chemical bonds or lattice matching offer significant integration advantages over conventional nonlinear materials. Moreover, their large second-order nonlinear susceptibility^20–22^ and highly tunable optical nonlinearity^23–25^ enable them as promising candidates for integrated NLO optoelectronics and photonics^26–28^. Compared to conventional nonlinear materials, 2D materials offer a broader material library for tailoring quantum light sources across diverse spectral ranges, along with enabling convenient optical tunability via novel engineering methods such as stacking^29^, twisting^30^, straining^31^, etc. Significantly, recent research has successfully demonstrated polarization entanglement in van der Waals materials, including MoS_2_^32^, WS_2_^33^ h-BN^34^ and r-BN^35,36^. Nevertheless, the development of 2D material-based quantum light sources is still in its infancy.

As the first reported 2D material-based nonlinear quantum source, ferroelectric van der Waals materials NbOCl_2_, with a distinctive distorted structure, exhibit several notable properties, such as minimal interlayer electronic coupling^33^, pronounced monolayer-like excitonic effect^34^, and strongly anisotropy^33,34^. The inherent ferroelectricity of NbOX_2_ breaks the inversion symmetry, leading to a significant second-order NLO response in both monolayer and bulk crystalline phases^27,37–39^. This behavior distinguishes NbOX_2_ from the van der Waals materials MoS_2_, WS_2_ and h-BN, which require the artificial alteration of the most stable stacking phase (2H) of the material to break the limitations of different odd-even symmetries, while NbOX_2_ inherently possesses this advantage. More importantly, unlike many 2D materials such as MoS_2_, WS_2_, and h-BN, the distinctive features of NbOX_2_ lie in its absence of fluorescence, strong in-plane anisotropy, and exceptionally large in-plane birefringence, which hold the potential to function as an ultrathin waveplate to manipulate quantum polarization states and directly achieve type‑II phase matching^40–42^. After being identified as SPDC source in the pioneering work^43^, the ultracompact polarization-entangled quantum sources using orthogonally stacked vdW NbOCl_2_ crystals were realized following closely^44,45^. More recently, Gao et al. achieved a significant enhancement of the quantum SPDC in NbOCl_2_ flakes by employing a metal-SiO_2_-nonlinear material heterostructure^34^. While NbOCl_2_ is well-established as a quantum light source, the potential of related compounds NbOBr_2_ and NbOI_2_ to function similarly remains unknown due to a lack of reported studies. Despite their isostructural nature, the slight differences in halogen atomic radii and electronegativities induce structural variations that could significantly affect their SPDC performances, warranting further investigation.

Here, we systematically investigate how individual crystal structure characteristics (e.g., polarization displacement and lattice distortion) and inherent material properties (such as refractive index dispersion and absorption) influence and modulate the performance of the SPDC process in the material. Our results show that, within the wavelength range of 800–1500 nm, the second-order optical nonlinear strength of NbOI_2_ generally exceeds that of NbOBr_2_. A notable increase in the second-order nonlinear optical coefficients can be observed at the absorption resonance energies, but the final nonlinear photon intensity is simultaneously influenced by the refractive index. The degree of nonlinear anisotropy varies among the materials, leading to distinct two-photon polarization states. Compared with NbOCl_2_ and NbOBr_2_, NbOI_2_ can directly generate polarization-entangled states at 914 nm with a concurrence of 0.90 ± 0.05. Additionally, NbOI_2_ has greater potential for quantum entangled photon sources in the short near-infrared wavelength, including in common near-infrared communication wavelengths of 1310 nm, Our work elucidates the connection between the crystal structure, intrinsic properties, and the quantum nonlinear SPDC process of NbOX_2_ materials, providing novel insights and guidance for both the targeted design of quantum light sources based on 2D materials, and the selection of appropriate 2D materials tailored to specific quantum light sources requirements.

## Results

### Crystal characterization

**Fig. 1:**
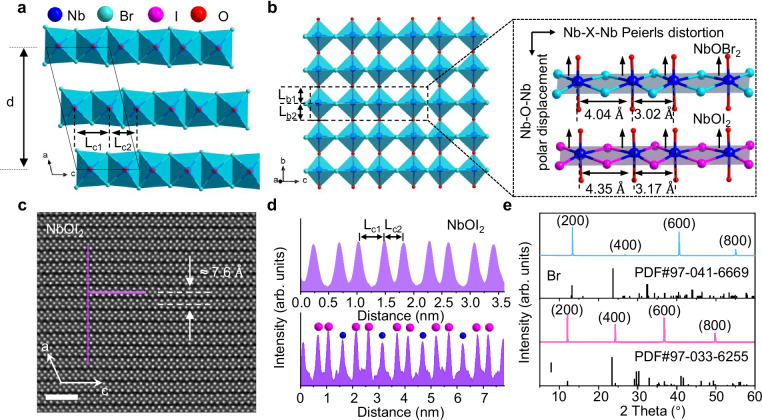
Structural characterization. Out-of-plane **a** and in-plane representations **b** of the crystal structure of NbOX_2_. The magnified area in (**b**) illustrates the structural distortion in NbOBr_2_ (top) and NbOI_2_ (bottom), highlighting the alternating Nb−Nb distances along the *c*-axis and the off-centering of Nb atoms along the *b*-axis. **c** Cross-sectional STEM image of NbOI_2_ viewed along the *b-*axis. Scale bar: 2 nm. **d** Line intensity profile of NbOI_2_ derived from (**c**), showing the alternating unequal Nb–Nb distances (top) along the *c*-axis and layer-to-layer distances over ten layers (bottom). **e** The XRD patterns of NbOBr_2_ (top) and NbOI_2_ (bottom).

Bulk NbOX_2_ crystallizes in a ferroelectric *C*2 space group, which is formed by the stacking of NbOX_2_ monolayers, with each layer shifting a constant distance along the out-of-plane direction (Fig. 1a). Similar to perovskite crystal structures^21^, each NbOX_2_ layer is composed of a mixture of distorted corner-sharing and edge-sharing NbO_2_X_4_ octahedra. Within these octahedra, Nb^4+^ is bonded to two equivalent O^2−^ ions along the *b*-axis and four X^−^ ions along the *c*-axis (Fig. 1b). As illustrated in the magnified view of Fig. 1b, a significant anisotropic bonding is observed along the in-plane two orthogonal. A first-order Peierls distortion along the *c*-axis induces the dimerization of niobium atoms, resulting in alternating Nb–Nb distances (L_c1_ ≠ L_c2_). The Peierls distortion parameter λ = |(L_c1_ − L_c2_)/(L_c1_ + L_c2_)| is introduced to quantify the degree of Peierls distortion in NbOX_2_^46^. Based on the Nb-Nb distances (L_c1_ and L_c2_) obtained from the enlarged Fig. 1b, Supplementary Fig. 1, the calculated value of λ_Br_ is 0.14, falling between λ_Cl_ (0.12) and λ_I_ (0.16). The Peierls distortion parameter λ is sensitive to the size of the halide anions. Accordingly, the degree of Peierls distortion along the *c*-axis increases as the radius of the halide anions increases. By contrast, a second-order distortion occurs along the *b*-axis, with niobium atoms deviating toward one of the corner oxygen atoms in the [NbO_2_X_4_] octahedral (Supplementary Fig. 2). Due to the high electronegativity of the Cl anion, NbOCl_2_ exhibits the largest spontaneous polarization and a maximum Nb polar displacement of 0.19 Å, followed by NbOBr_2_ (0.15 Å) and NbOI_2_ (0.13 Å). The structural asymmetry along the *b*-axis is most pronounced in NbOCl_2_ and least in NbOI_2_. The different anions in the NbOX_2_ family lead to distinct structural distortions and asymmetric distribution of niobium elements, thereby influencing the SHG properties^46,47^. Notably, the polarization displacement along the *b*-axis may lead to variations in SHG anisotropy.

High-angle annular dark-field scanning transmission electron microscopy (HAADF-STEM) was employed to investigate the structure of the NbOX_2_. As depicted in Fig. 1c, the layered arrangement of NbOI_2_ along the *a*-axis and the structural Peierls distortion along the *c*-axis can be clearly observed. These features are confirmed by the corresponding line intensity profile images (Fig. 1d). NbOI_2_ exhibited a layer thickness of approximately 7.6 Å (Fig. 1d, bottom and Supplementary Fig. 3b), whereas NbOBr_2_ displayed a thickness of approximately 6.9 Å (Supplementary Fig. 4b, bottom). Single-crystal XRD further confirms the single-crystalline nature and isomorphous structure of the two materials. Compared to NbOBr_2_, the diffraction peaks of NbOI_2_ are shifted to smaller angles, indicating its larger *a*-axis lattice parameter. This finding aligns well with measurements obtained via STEM.

**Fig. 2:**
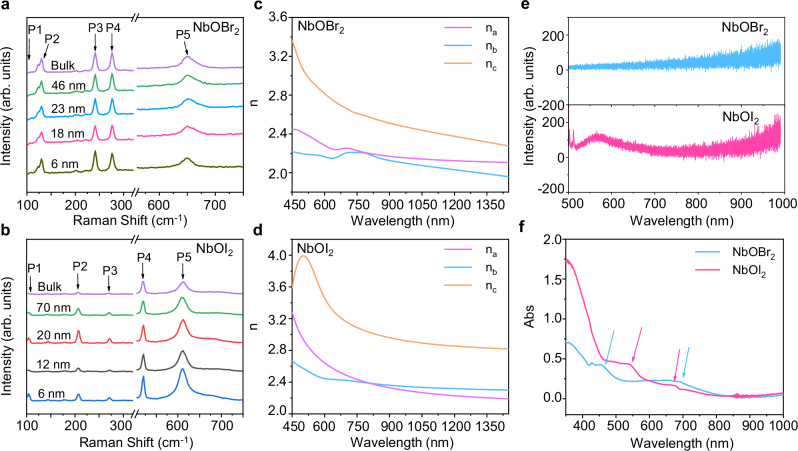
Intrinsic optical property characterization. **a**,** b** Raman spectra of 2D NbOBr_2_ (**a**) and NbOI_2_ (**b**). **c**,** d** Experimentally measured dispersion curves of refractive indices of NbOBr_2_ (**c**) and NbOI_2_ (**d**), where *n*_*a*_ denotes the refractive index perpendicular to the *b*-axis in the (100) plane, *n*_*b*_ and *n*_*c*_ represents the refractive index along *b*- and *c*-axes, respectively. **e** PL spectra of NbOBr_2_ (top) and NbOI_2_ (bottom). **f** Experimental absorption spectrum of NbOBr_2_ (blue line) and NbOI_2_ (pink line).

Due to the weak vdW forces^48^ and strong crystallographic anisotropy, the NbOX_2_ crystal can be easily exfoliated into monolayer or few-layer flakes with sharp edges. The optical image and the detailed atomic force microscopy (AFM) image of 2D NbOX_2_ flakes on a silicon wafer with a 285 nm oxidation layer are displayed in Supplementary Figs. 5, 6. An obvious optical contrast was observed between flakes with different thicknesses. The corresponding Raman spectra measured under laser excitation wavelength at 488 nm were performed to study the optical properties of NbOX_2_ (Fig. 2a, b). Five peaks are resolved within the spectral range and denoted as P1 to P5 from low to high Raman shift. The lower-energy peaks (P1–P4) are primarily governed by vibrations involving the Nb–X bonds and collective motions of the octahedral framework, while the higher-energy peaks P5 arise from vibrations of the stronger Nb–O bonds^49^. Polarization-resolved Raman spectroscopy reveals distinct behavior in the niobium oxyhalides: in NbOBr_2_, Raman modes P1–P4 display A- and P5 displays B- symmetry modes, whereas in NbOI_2_, all observed Raman peaks exclusively exhibit A- symmetry modes, thereby signifying a significant anisotropy within the NbOX_2_ crystal structure^49^ (Supplementary Figs. 7, 8). Unlike TMDCs and many other 2D materials^50,51^, the peak positions in NbOX_2_ remain almost constant with increasing thickness (Fig. 2a, b), indicating the weak interlayer coupling nature in NbOX_2_. The intrinsic Peierls distortion of NbOX_2_, combined with the weak interlayer coupling, ensures that stacking along the *a*-axis does not modify the material’s symmetry, resulting in a second-order nonlinear response that can scale with thickness. The irregular intensity evolution of Raman peaks with thickness arises from thin-film optical interference, and the anisotropic vibrational characteristics of different modes and anisotropic dielectric screening effect of the crystal, rather than structural or symmetry changes^52,53^. Calculated dielectric constants verify that strong intrinsic in-plane dielectric constants give rise to robust bulk screening, which stabilizes phonon frequencies against thickness-induced interfacial dielectric perturbations^54^. Meanwhile, the large in-plane/out-of-plane dielectric anisotropy, coupled with visible-band resonant absorption, renders these out-of-plane modes susceptible to interfacial screening fluctuations and thus induces irregular intensity changes^55^ (Supplementary Fig. 9). The P1 and P5 modes are mainly contributed by in-plane vibrations along the *b*-axis, while the P2, P3, and P4 modes are primarily associated with out-of-plane vibrations^56^. The observed shift in Raman peak position between the two materials is considered to correlate with the difference in lattice parameters and the different halogen elements between the two materials. The exfoliated NbOX_2_ flakes are relatively stable under ambient conditions (Supplementary Figs. 10, 11). Owing to the stronger electronegativity and shorter bond length of Br compared to I, the Br atom in NbOBr_2_ acquires more charge from Nb atoms than the I atom in NbOI_2_. This enhanced interatomic interaction stabilizes the surface structure, resulting in better stability for NbOBr_2_. The exfoliated NbOBr_2_ flakes remain stable under ambient conditions with no obvious changes over two weeks, whereas NbOI_2_ shows no significant changes within one week.

Figure 2c, d show the experimentally measured dispersion curves of refractive indices for NbOBr_2_ and NbOI_2_, respectively. NbOX_2_ is a biaxial crystal and exhibits strong optical anisotropy with large in-plane birefringences (Δn = 0.39 for NbOBr_2_, 0.6 for NbOI_2_ at 900 nm), which are larger than those of most conventional birefringent materials^57^. The calculated optical constants along the three axes (Supplementary Fig. 12) show excellent agreement with the experimental data. Notably, the in-plane birefringence values of NbOI_2_ exceeded those of NbOBr_2_ in the wavelength range of 560 nm to 920 nm, indicating significantly greater anisotropy of NbOI_2_ within this spectral range. This large in-plane birefringence may offer several potential advantages in SPDC. First, it can serve as an ultrathin waveplate to manipulate the polarization state of photon pairs, enabling the design of biphoton polarization states beyond the limits imposed by the nonlinear coefficient matrix and opening a new route for more complex quantum states. Second, its large birefringence may allow direct satisfaction of type‑II phase matching by tuning the pump polarization angle and wavelength—without requiring additional structural engineering—thereby significantly enhancing SPDC efficiency. The photoluminescence (PL) signal of NbOBr_2_ and NbOI_2_ under 488 nm excitation was measured (Fig. 2e). Since NbOX_2_ is an indirect bandgap semiconductor and difficult to excite, the smooth straight line of NbOX_2_ indicates PL is inactive. This inactive PL signal reduces background noise during nonlinear excitation, lowers accidental counts, simplifies the isolation of entangled pairs and enhances the clarity of the quantum signal. The micro-optical absorption measurement of NbOX_2_ nanoplate was acquired over the wavelength range of 350–1000 nm (Fig. 2f). NbOBr_2_ exhibited a predominant peak at about 470 nm and a broad peak near 700 nm, while NbOI_2_ showed two main peaks at approximately 550 nm and 700 nm. These absorption characteristics of NbOBr_2_ and NbOI_2_ are crucial for their second-order optical susceptibility, which we will discuss in the SHG section. Apart from the above observations, NbOI_2_ exhibits a prominent absorption peak at 350 nm. It is attributed to high-energy excitonic transitions in the polar (*b*-axis) direction and is expected to significantly enhance the second-order nonlinear susceptibility^27^. Restricted by experimental conditions, we were unable to measure the SHG and SPDC photons at this wavelength. Nevertheless, we believe the ultraviolet absorption peaks of these two materials show great potential for ultraviolet lasers and visible quantum light sources.

### SHG of NbOX_2_

**Fig. 3:**
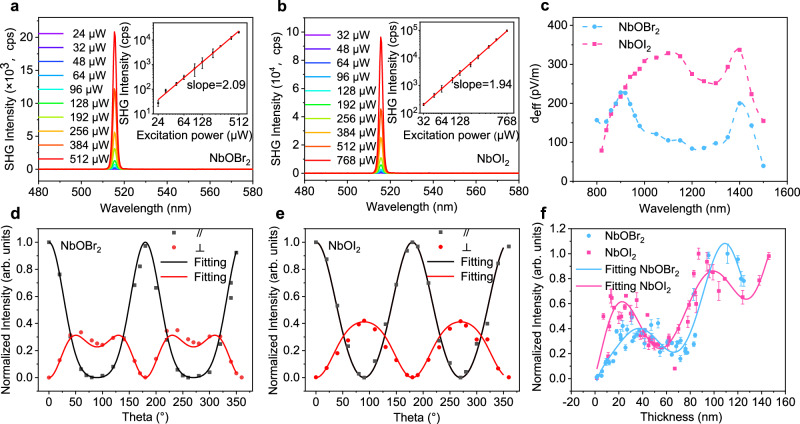
Anisotropic and scalable SHG response of the NbOX_2_. **a**,** b** Power-dependent-SHG spectra of NbOX_2_. Insets, the excitation power dependence of SHG intensity with the slope fitted to be 2.09 for NbOBr_2_ (**a**) and 1.94 for NbOI_2_ (**b**), indicating the quadratic nature of the nonlinear optical process. (cps: counts per second; Laser spot radius: 1 micrometer). **c** Wavelength-dependent effective second-order nonlinear coefficient of NbOBr_2_ (blue circles) and NbOI_2_ (pink squares). **d**,** e** Polarization-resolved SHG intensity of NbOBr_2_ (**d**)and NbOI_2_ (**e**) along parallel (black squares) and perpendicular (red circles) direction. The zero degree is aligned with the crystallographic *b*-axis. **f** Thickness-dependent SHG intensity of NbOBr_2_ (blue circles) and NbOI_2_ (pink squares) in a broader thickness range under a back-reflection configuration with 1030 nm wavelength pump. Error bars represent the standard deviation of three independent measurements.

The second-order NLO response of NbOX_2_ was investigated through SHG test experiments under a back-reflection configuration. Figure 3a, b clearly display SHG signals from NbOBr_2_ and NbOI_2_ (thickness of 100 nm) pumped by a 1030 nm wavelength laser. The representative quadratic dependence of the SHG intensity on the pump power was demonstrated in both materials with a fitted slope of 2.09 and 1.94, respectively. The results also reveal that NbOI_2_ possess stronger SHG response than NbOBr_2_ under 1030 nm wavelength. We further measured the dispersion curves of the effective second-order nonlinear coefficient of the NbOX_2_ via different wavelengths. The measured wavelength-dependent effective second-order nonlinear coefficients for the NbOX_2_ in Fig. 3c, comparing with the standard LN sample (500 nm thick) and calculated by the method reported in Ref. ^43^ (see Supplementary Section 4), show the optimal 2^nd^ order optical nonlinear changing with wavelength for the two materials. The enhanced effective second-order nonlinear coefficient, attributing to the resonantly coupling between SHG signal photons and materials’ electronic interband transitions^33^, is consistent with the result of the optical absorption spectrum in Fig. 2f. In contrast of NbOCl_2_ which is more suitable to prepare visible SPDC sources^43^, NbOBr_2_ is appropriately to produce SHG signal at wavelength approximately 460 nm/700 nm or SPDC photon pairs at wavelength approximately 920 nm/1400 nm, while NbOI_2_ is expected to generate even stronger SHG signal at wavelength around 550/700 nm and SPDC photon pairs at wavelength approximately 1100 nm/1400 nm, making it a potential quantum material preparing entangled photons at short near-infrared band.

Based on the *C*2 space group^58^ of NbOX_2_ crystals, the second-order nonlinear coefficient matrix is expressed as:

$$\left[\begin{array}{cc}\begin{array}{ccc}0 & 0 & 0\end{array} & \begin{array}{ccc}{\chi }_{{abc}}^{(2)} & 0 & {\chi }_{{aab}}^{(2)}\end{array}\\ \begin{array}{ccc}{\chi }_{{baa}}^{(2)} & {\chi }_{{bbb}}^{(2)} & {\chi }_{{bcc}}^{(2)}\end{array} & \begin{array}{ccc}0 & {\chi }_{{bac}}^{(2)} & 0\end{array}\\ \begin{array}{ccc}0 & 0 & 0\end{array} & \begin{array}{ccc}{\chi }_{{cbc}}^{(2)} & 0 & {\chi }_{{cab}}^{(2)}\end{array}\end{array}\right]$$

there are three matrix elements --$\,{\chi }_{{bbb}}^{(2)},\,{\chi }_{{bcc}}^{(2)}$ *and* ${\chi }_{{cbc}}^{(2)}({\chi }_{{ccb}}^{\left(2\right)})$ can be used when the crystals are pumped by normal incidence (along *a*-axis) under paraxial approximation. Therefore, the polarization-resolved SHG measurements of the NbOBr_2_ and NbOI_2_ are analyzed to evaluate the relative strength of these effective matrix elements. To reduce the interference effect of thickness, the experimental parallel component and perpendicular component of the two materials’ SHG were measured by thin films with a thickness below 50 nm. Although the structural symmetry is the same, the polarization-resolved SHG intensity of NbOBr_2_ and NbOI_2_ along parallel (black) and perpendicular (red) directions, as shown in Fig. 3d, e, exhibits different curve shapes, attributing to the different strength relations of the matrix elements for NbOBr_2_ and NbOI_2_. By adjusting the strength of matrix elements, the experimental data (dots) agree well with the corresponding theoretical results (curves). The fitting lines demonstrate that the ratio of ${\chi }_{{bbb}}^{\left(2\right)}/\,{\chi }_{{bcc}}^{\left(2\right)}\approx 2.10$ for NbOBr_2_ and ${\chi }_{{bbb}}^{\left(2\right)}/\,{\chi }_{{bcc}}^{\left(2\right)}\approx 1.56$ for NbOI_2_, while the contribution of ${{{{\rm{\chi }}}}}_{{cbc}}^{\left(2\right)}({{{{\rm{\chi }}}}}_{{ccb}}^{\left(2\right)})$ is negligible for both materials (see more details in Supplementary Section 5). These results contrast with NbOCl_2_, whose ratio of ${{{{\rm{\chi }}}}}_{{bbb}}^{(2)}/\,{{{{\rm{\chi }}}}}_{{bcc}}^{(2)}$ clearly exceeds NbOBr_2_ and NbOI_2_^45^, resulting in different polarization states in SPDC as demonstrated in the following text.

The thickness-dependent SHG intensity measured with NbOBr_2_ and NbOI_2_ under a fixed pump power of 500 μW (Fig. 3f) further proves the weak interlayer electronic coupling and the non-centrosymmetric structure when NbOX_2_ stacked layer-by-layer. As expected, a similar phenomenon is observed in NbOBr_2_ and NbOI_2_, when the interaction length is very thin, the SHG intensity exhibits a nearly squared relationship with the crystal thickness. But as the thickness continues to increase, the interference among the SHG from surface effect and from different depths of the crystal should be taken into account, resulting in the peaks and dips^59^. It should be noted that Fig. 3f is measured under a back-reflection configuration, while the coherence lengths of the same materials under a transmission configuration are much longer (see Supplementary Section 6). In addition, given that the coherence length is wavelength-dependent, the thickness corresponding to the maximum SHG intensity also varies with wavelength. By selecting an appropriate thickness, the SHG efficiency can be optimized for specific target wavelengths. The various thicknesses are confirmed by the AFM results (Supplementary Figs. 13, 14).

### SPDC of NbOX_2_

**Fig. 4:**
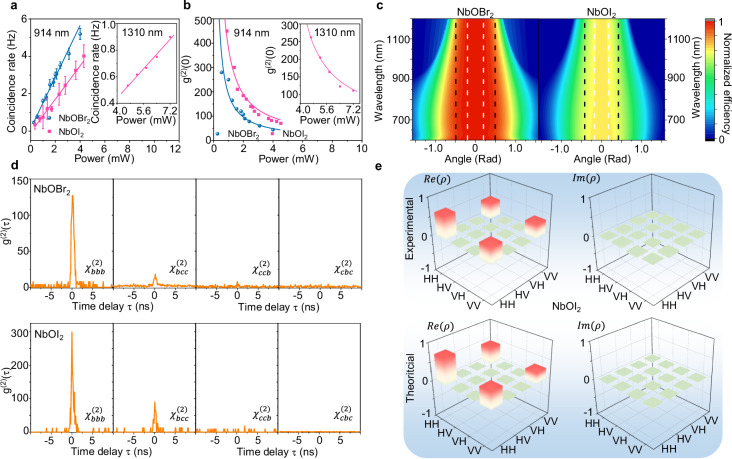
Results of the quantum SPDC process with NbOBr_2_ and NbOI_2_. **a**,** b** Power-dependent coincidence rates and ${g}^{\left(2\right)}(0)$ of NbOBr_2_ (145 nm thick, blue circles) and NbOI_2_ (140 nm thick, pink squares) under 457 nm pump, fitted by proportional and inverse functions, respectively. Insets, near-infrared SPDC sources based on NbOI_2_ (thickness ~100 nm) under pump at 655 nm wavelength. Error bars represent the standard deviation of three independent measurements. **c** Calculated wavelength-angular spectrum of the emission SPDC photons from NbOBr_2_ (left) and NbOI_2_ (right) engineered by ${\chi }_{{bbb}}^{(2)}$, and the relative efficiencies are normalized by $1/({l}^{2}\cdot {{d}_{{eff}}}^{2})$. **d** Measured second-order correlation functions of the photons of NbOBr_2_ (top) and NbOI_2_ (bottom), engineered by different effective nonlinear matrix elements under the same pump power. **e** Experimental (top) and corresponding expected theoretical density matrix (bottom) of the SPDC photons generated from NbOI_2_ under H-polarized pump at 914 nm, which polarization direction is along the *b*-axis. Real part (left), Imaginary part (right).

The low fluorescence signal due to the indirect bandgap structure of NbOX_2_ provides a broad available bandwidth for the preparation of quantum entangled photon sources with minimal background interference. We selected a 457 nm pump wavelength where both NbOBr_2_ and NbOI_2_ exhibit relatively high and comparable effective second-order nonlinear optical coefficients, as demonstrated in Fig. 3c, to directly compare the quantum SPDC process in these two crystalline materials. Experimental characterization presented in Fig. 4 reveals that both materials generate well-defined twin photon pairs, as confirmed by our home-built Hanbury Brown-Twiss (HBT) setup (Supplementary Fig. 17). More details of the experimental configurations of the SPDC measurements can be found in the method.

The power-dependent coincidence rates of 145 nm-thick NbOBr_2_ and 140 nm-thick NbOI_2_ were measured to evaluate the brightness of these two SPDC sources, both of which exhibit a characteristic linear power dependence of the SPDC process, as shown in Fig. 4a. Notably, despite possessing a slightly lower effective nonlinear coefficient, NbOBr_2_ demonstrates a superior coincidence rate than NbOI_2_. This anomalous contrast can be mainly attributed to three reasons. Firstly, the coherence length of the main contribution element ${{{{\rm{\chi }}}}}_{{bbb}}^{(2)}$ for NbOBr_2_ (~2450 nm) is significantly longer than the counterpart for NbOI_2_ (~775 nm)(see Supplementary Section 6). This implies the degree of phase mismatch for NbOI_2_ is greater than that for NbOBr_2_ under this specific thickness and wavelength of the experiment, resulting in a faster decreased normalized relative efficiency (Supplementary Fig. 16). Secondly, the efficiency of second order nonlinear optical conversion inversely proportional to the refractive index of the participating photons: ${{{\rm{\eta }}}}\propto \frac{1}{{n}_{i}\cdot {n}_{s}\cdot {n}_{p}}$^60,61^, while the refractive index contrast at wavelength of pump light and SPDC photon pairs both exhibit n(NbOI_2_) > n(NbOBr_2_) (Fig. 2c, d). Figure 4c shows the corresponding calculated wavelength-angular spectrum of the emission SPDC photons engineered by ${\chi }_{{bbb}}^{(2)}$^19^, where the displayed relative generation efficiencies are normalized by thickness and respective effective second-order optical nonlinear coefficient ($1/({l}^{2}\cdot {{d}_{{eff}}}^{2})$). Here, the calculated lower efficiencies of NbOI_2_ compared to NbOBr_2_ confirm the influence of longitudinal phase mismatch and index contrast. The broad bandwidth for frequency of SPDC photons from thin NbOX_2_ has been further demonstrated in experiment (Supplementary Fig. 18), the measured spectrum is restricted by the working bandwidth of the detectors. Thirdly, the calculated angular spectrum of radiation broadening in the thin-film SPDC process is clearly revealed in Fig. 4c, where black and white dashed lines indicate the total reflection angle and the practical collection angle considering the N.A. of objective, respectively. Higher refractive index also induces substantial total internal reflection and smaller collection angle, trapping a considerable portion of generated SPDC photons within the NbOI_2_ crystals. These findings further establish refractive index as an informative factor governing nonlinear optical process, influencing not only the generation efficiency, but also the collection efficiency in experiments. The power-dependent second-order correlation functions were also measured in Fig. 4b, exhibiting a high coincidence to accidental ratio (CAR) and consistent inversely proportional relations, another typical evidence of the SPDC process.

Specifically, the NbOBr_2_ crystal achieves a brightness of 1.5 Hz/mw at 914 nm, which is comparable to the performance of the NbOCl_2_ crystal at 808 nm in Ref. ^43^. Considering the detection efficiency of our experimental system, estimated as around 0.26%, this corresponds to a normalized merit value of 577 Hz/mw (see in methods, SPDC measures). Another appropriate pump wavelength for SPDC in NbOBr_2_ is expected at 700 nm. As for NbOI_2_, dispersion analysis of d_eff_ and refractive index suggests enhanced suitability for entangled photon generation at longer wavelengths, with quantum sources operating at 1100 nm and 1400 nm for instance, it is projected to surpass the brightness of NbOBr_2_ at 914 nm and NbOCl_2_ at 808 nm. To investigate the potential for NbOX_2_ in preparing short near-infrared quantum sources, we also measured the SPDC process for the two materials at 1310 nm, one of the fiber optic transparent windows and a widely used wavelength for communication. The experimental results shown in the inset of Fig. 4a, b, demonstrate that NbOI_2_ is potential for generating the entangled photons at this important wavelength, while NbOBr_2_ is much inferior (Supplementary Fig. 19). The measured 1310 nm coincidence rates were limited by the single-mode fiber’s collection efficiency, corresponding to approximately half the efficiency achieved with multi-mode fiber collection in 914 nm SPDC measurements (see more details in “Methods”).

The primary tensor elements ${{{{\rm{\chi }}}}}_{{bbb}}^{(2)}$,$\,{{{{\rm{\chi }}}}}_{{bcc}}^{(2)}$ and ${{{{\rm{\chi }}}}}_{{cbc}}^{\left(2\right)}({{{{\rm{\chi }}}}}_{{ccb}}^{\left(2\right)})$ were then directly characterized through the polarization-resolved SPDC measurement, in which the polarization of pump light and collected signal/idler photons have been controlled correspondingly. The second-order correlation analyses in Fig. 4d revealed tensor elements dominance between SPDC and SHG measurements—${{{{\rm{\chi }}}}}_{{bbb}}^{(2)}$ predominantly governs the SPDC process with negligible contribution from ${{{{\rm{\chi }}}}}_{{cbc}}^{\left(2\right)}({{{{\rm{\chi }}}}}_{{ccb}}^{\left(2\right)})$ in both materials, mirroring their SHG response hierarchy. Additionally, NbOI_2_ exhibits a higher $\,{{{{\rm{\chi }}}}}_{{bcc}}^{(2)}/\,{{{{\rm{\chi }}}}}_{{bbb}}^{(2)}$ ratio compared to NbOBr_2_, also consisting with the SHG results. Consequently, this progression leads to distinct polarization states from the reported NbOCl_2_^43,44^. While NbOCl_2_ at 808 nm demonstrates remarkable in-plane anisotropy approximating a type-0 SPDC condition, NbOI_2_ possesses a relative balanced $\,{{{{\rm{\chi }}}}}_{{bbb}}^{(2)}$ and $\,{{{{\rm{\chi }}}}}_{{bcc}}^{(2)}$ tensor contribution, enabling direct generation of polarization entangled state when pumped with *b*-axis linearly polarized light. Quantum state tomography using a 120 nm-thick NbOI_2_ crystal confirmed this mechanism (Fig. 4e). The experimental reconstructed density matrix agrees well with the theoretical two-photon polarization state 0.84|HH〉 + 0.54|VV〉, predicted according to the ratio derived from Fig. 3e (see Supplementary Section 7). The fidelity between the experimental result and the theoretical one achieves F = 0.94 ± 0.03, and the concurrence (entanglement degree) of the experimental density matrix achieves C = 0.90 ± 0.05, in contrast with the nearly pure state |HH> generated with one piece of NbOCl_2_ crystal. These results demonstrate that the polarization-entangled state with high concurrence at 914 nm can be prepared from a monolithic NbOI_2_ sample, eliminating the requirement of stacking in the case of NbOCl_2_^44^.The polarization states exhibited by NbOBr_2_ are anticipated to lie between those of NbOCl_2_ and NbOI_2_.

In conclusion, we systematically compared classical and quantum second-order nonlinear optical processes based on NbOBr_2_ and NbOI_2_, demonstrating their potential as SPDC quantum light sources. Notably, NbOI_2_ exhibited a superior effective second-order nonlinear optical coefficient across the entire near-infrared spectral range, rendering it more suitable than NbOBr_2_ for SPDC applications at common near-infrared communication wavelengths of 1310 nm. Furthermore, when pumped with 914 nm *b*-axis linearly polarized light, NbOI_2_ could directly generate polarization-entangled states with concurrence of 0.90 ± 0.05 and fidelity of 0.94 ± 0.03. In comparison, NbOBr_2_ is better suited as a source for SPDC at shorter wavelengths. Through detailed characterization and investigation of the respective crystal structures and intrinsic properties of the materials, we elucidated the influence of lattice distortions and absorption on the effective second-order nonlinear optical coefficient. Further, the measured intensity of the nonlinear light source is strongly influenced by the refractive index dispersion. In addition, we also analyzed the coincidence-accidence ratio and two-photon polarization state of the SPDC source modulated by NbOX_2_. Our work establishes a connection between the intrinsic material physicochemical properties of 2D materials and their SPDC nonlinear optical behavior, providing a novel perspective for understanding the SPDC response of various 2D materials.

Within the context of the extensive and evolving 2D material library, our study offers insights and assistance for the development of future quantum light sources. Despite achieving brightness comparable to the brightest 2D materials for SPDC reported to date (Table S1), this ultra-compact and ultra-thin quantum light source is still in the early research stage, and its absolute brightness remains far from practical application levels. To overcome this limitation, future work will focus on combining this van der Waals platform with advanced micro- and nano-optical architectures, such as cavity-photonics, metasurfaces, and integrated optical platforms, we anticipate realizing significant advancements in both scientific research and applications, propelling the development of ultra-compact quantum light sources, which will further advance frontiers in the fields of quantum computing, quantum sensing, and quantum simulation.

## Methods

### Crystal growth

Crystalline NbOBr_2_ and NbOI_2_ were grown by the chemical vapor transport method. The reagents of Nb, Nb_2_O_5_ and hexabromobenzene (C_6_Br_6_) with 9:3:5 were used as precursors for the growth of NbOBr_2_. The mixture of precursors was placed in a quartz ampule, and the ampule was sealed after being evacuated (10^−5^ Torr). Similarly, Nb, iodine, and Nb_2_O_5_ with 1:1:2 were used to grow NbOI_2_. The sealed quartz ampules were placed at the center of a horizontal dual-zone furnace. Both heating zones were slowly heated to 650 °C, kept at this temperature for 7 days and cooled to room temperature with the furnace cooling naturally. Finally, the shiny black rectangle crystals were collected from the cold end of the tube. In order to avoid degradation, the NbOX_2_ crystals are stored in an N_2_-filled glovebox.

### Characterization

The morphology and thickness of the exfoliated NbOX_2_ were characterized by optical microscope (DP27, Olympus) and atomic force microscope (Biope System, Bruker). The crystal structure of the exfoliated flakes was performed by HAADF-STEM (JEOL JEM-ARM200F, 200 kV). The XRD patterns of NbOX_2_ crystal were measured by an XRD Bruker D8 Advance Powder with Cu−K*α* target with the angle ranging from 5° to 60°.The Raman and PL spectra were obtained using a confocal microscope (inVia Reflex, Renishaw with a 488 nm laser as the excitation source). To detect the polarized Raman spectra, the incident laser was polarized, and the scattering light was in the direction parallel and perpendicular to the incident light. The birefringence data were measured using a commercial spectroscopic ellipsometer (J.A. Woollam RC2).The optical absorption spectra were measured using Microscopic UV-Vis-NIR spectrometer (MSV-5200).

### SHG·Measurements

The reflected SHG mesasurements were obtained in a self-made confocal microscope optical system. A 1030 nm pulse laser with 80 MHz repetition rate was focused onto NbOX_2_ nanaosheets by a 100× objective (Olympus·MPlanFL·N, NA = 0.9). The SHG signal was collected by the same objective, and filtered through a 650 nm short-pass dichroic filter to block the excitation laser in front of the spectrometer. The wavelength-dependent and polarization dependent SHG in Fig. 3c–e were measured under transmission geometry using a supercontinuum laser operating at 80 M Hz repetition rate and a pulse duration of 6 ps. The pump light was focused onto the sample with a 50× objective (NA = 0.42), and the generated SHG signal was collected by another 10× objective (NA = 0.25). The polarization-dependent SHG measurements in Fig. 3d, e were pumped by 914 nm wavelength. To evaluate the second-order susceptibility χ^2^, we employ *x*-cut LiNbO_3_ (500 nm thick, d = 40 pm/V) as a standard material. We conducted SHG measurements on both the LiNbO_3_ and the sample, maintaining consistent excitation conditions. The resulting χ^2^ values were then computed using the method reported in Ref. ^43^.

### SPDC measurements

The experimental setup for quantum-correlation and quantum-state tomography measurements is illustrated in Supplementary Fig. 17. A 457 nm continuous-wave laser is used as the pump and focused by an objective (NA = 0.25) onto the vdW crystals (NbOBr_2_ and NbOI_2_). The generated SPDC twin photons are collected by another objective (NA = 0.42) and separated by a polarization-independent beam splitter. A 900 nm long-pass filter is used to remove the pump light. After being respectively projected into different polarization state, signal and idler photons are coupled into the multimode fibers to be detected and analyzed by the TCSPC setup. For SPDC measurements at wavelength 1310 nm, the experimental setup is similar, only replacing the pump light with a 655 nm continuous-wave laser, and the generated photons were measured by the superconducting detectors after being collected by the single-mode fiber. Notably, the two HWP, QWP and PBS on the collection optical path are absent when measuring the power-dependent coincidence rates and second-order correlation functions in Fig. 4a, d. Results in Fig. 4c were calculated for 145 nm thick NbOBr_2_ and 140 nm thick NbOI_2_, based on the dispersion data in Fig. 2c, d. The calculation arise from three conserted equations simultaneously--energy conservation, transverse momentum conservation and the modulation of the longitudinal momentum mismatch.

### Calculation methods

All calculations were carried out for the material in the framework of Density Functional Theory (DFT) using the Vienna Ab initio Simulation Package (VASP 6.3.2). The generalised gradient approximation (GGA) of the Perdew-Burke-Ernzerhof (PBE) function was used to describe the exchange-correlation energy. The projected augmented wave (PAW) method and pseudopotentials were used to describe the interactions between valence electrons and ions. For the atomic displacement calculations, the Brillouin zone was sampled by a 2 × 6 × 4 k-mesh generated according to the Monkhorst-Pack (MP) scheme, and for the calculation of birefringence, a 1 × 8 × 5 k-point grid was utilized, also under the MP scheme, with an energy cutoff of 500 eV. The total energies and the forces were converged to less than 10^−5^ eV and 0.01 eV/Å, respectively.

## Supplementary information

Supplementary Information

Transparent Peer Review file

## Source data

Source Data

## Data Availability

The relevant data generated in this study are provided in the Supplementary Information/Source Data file. Source data are provided with this paper.
