# Supplementary material for: Nonlinear quantum light source with van der Waals ferroelectric NbOX2 (X = Br, I)
Source: Nat Commun. 2026 Jun 30;17:8148. doi: 10.1038/s41467-026-75018-4 (PMC13458526; doi:10.1038/s41467-026-75018-4)
Supplement: Supplementary file 1 [file 41467_2026_75018_MOESM1_ESM.pdf]

Supplementary materials for

Nonlinear quantum light source with van der Waals ferroelectric $NbOX_2$ (X=Br, I)

Yanru Wang[1,#], Yunkun Wu[2,3,4,#], Yong Liu[5,6], Wanfu Shen[7], Ya Feng[8], Yue Liu[2,3,4], Chen Yi[8], Kun He[8], Miaomiao Liu[1], Ping Lu[1], Shaojun Liu[8], Yingying Liu[1], Jingmei Tang[1], Liqiang Zhang[1], Chunguang Hu[7], Shula Chen[5,6], Guangcan Guo[2,3], Xifeng Ren[2,3,4,*], Xidong Duan[1,*]

[1]Hunan Key Laboratory of Two-Dimensional Materials State Key Laboratory for Chemo/Biosensing and Chemometrics College of Chemistry and Chemical Engineering Hunan University Changsha 410082, China

[2] Laboratory of Quantum Information, University of Science and Technology of China, Hefei, China.

[3]CAS Center for Excellence in Quantum Information and Quantum Physics, University of Science and Technology of China, Hefei, China.

[4]Hefei National Laboratory, University of Science and Technology of China, Hefei 230088, China

[5]Hunan Institute of Optoelectronic Integration and Key Laboratory for MicroNano Physics and Technology of Hunan Province, Hunan University, Changsha, China

[6]College of Materials Science and Engineering, Hunan University, Changsha, China

[7]State Key Laboratory of Precision Measuring Technology and Instruments, Tianjin University, Weijin Road 92, Nankai District, Tianjin 300072, China.

[8]Hunan Provincial Key Laboratory of Two-Dimensional Materials, School of Physics and Electronics, College of Semiconductors (College of Integrated Circuits), Hunan University, Changsha, 410082 China

[#]These authors contributed equally: Yanru Wang and Yunkun Wu

*Corresponding author. E-mail: xidongduan@hnu.edu.cn, renxf@ustc.edu.cn;

Supplementary Note 1. Crystal characterization of $NbOX_2$

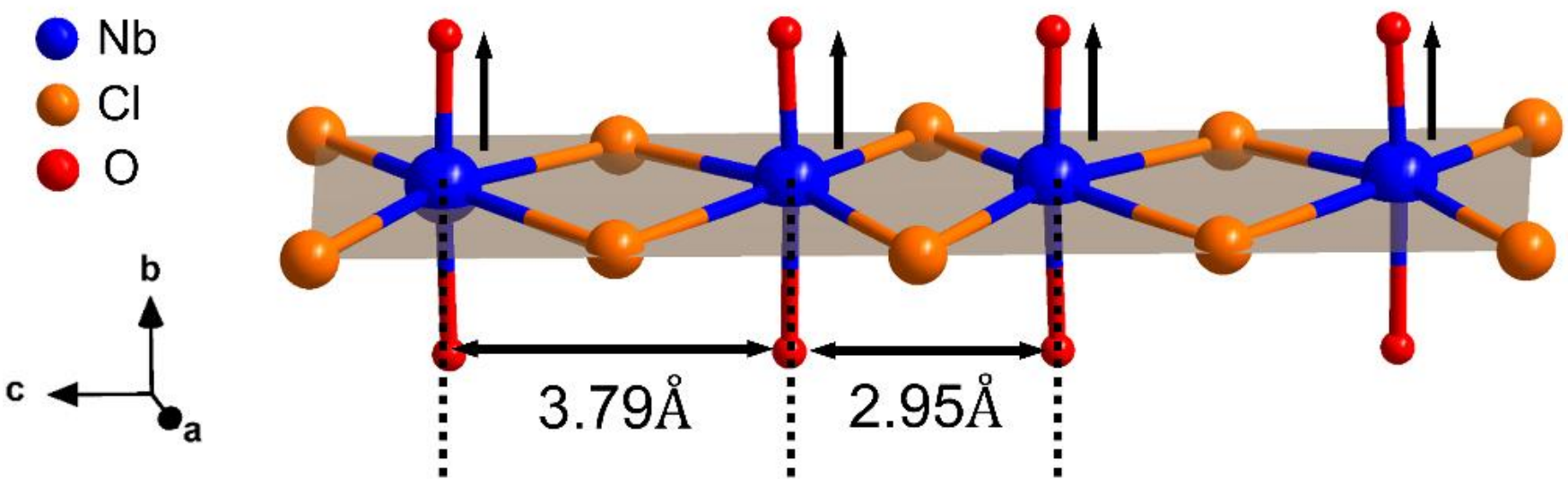

Supplementary Figure S1. A schematic illustration of the distortion of $NbOCl_2$. The alternating Nb−Nb distances along the *c*-axis and the off-centering of Nb atoms along the *b*-axis can be clearly seen.

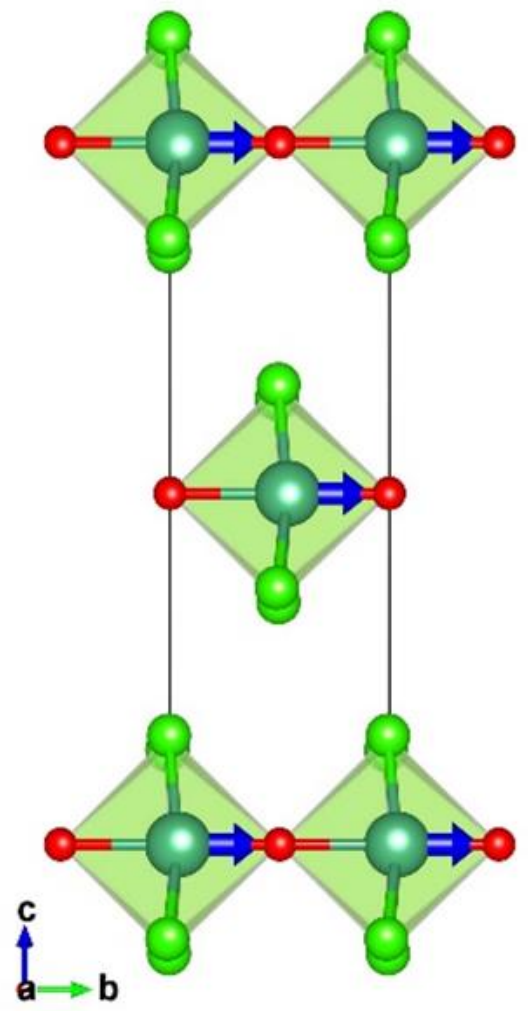

Supplementary Figure S2. Schematic illustrations of the FE phase $NbOX_2$ created using VESTA. The calculated displacement of the Nb atom along *b*-axis within the [NbO_2X_4] octahedron towards one of the corner oxygen atoms, with values of 0.19, 0.15, and 0.13 Å for $NbOCl_2$, $NbOBr_2$, and $NbOI_2$, respectively.

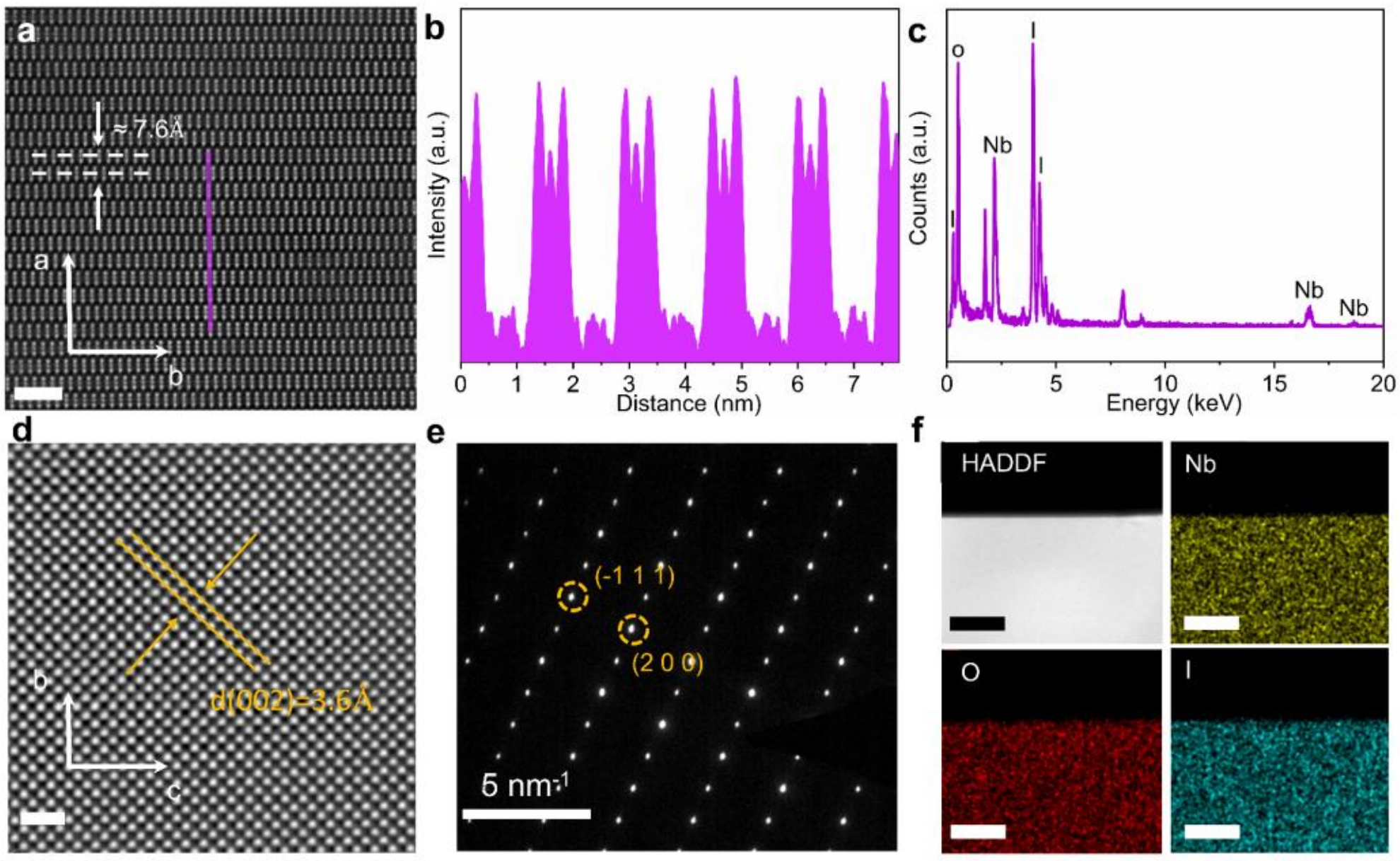

Supplementary Figure S3. **Structural characterization of $NbOI_2$. a,** HAADF-STEM images of $NbOI_2$ viewed along the c axis. Scale bar: 2 nm. **b,** Line intensity profile of $NbOI_2$ in **a**, showing the ten layers thick distances. a.u., arbitrary units. (Scale bar 2 nm). **c,** The EDS spectra of $NbOI_2$. **d,** STEM image of $NbOI_2$ crystal viewed along the *a* axis. Scale bar: 1 nm. **e,** SAED pattern of $NbOI_2$. **f,** HAADF-TEM image of and $NbOI_2$ and the corresponding EELS elemental mapping images. Scale bar, 50 nm.

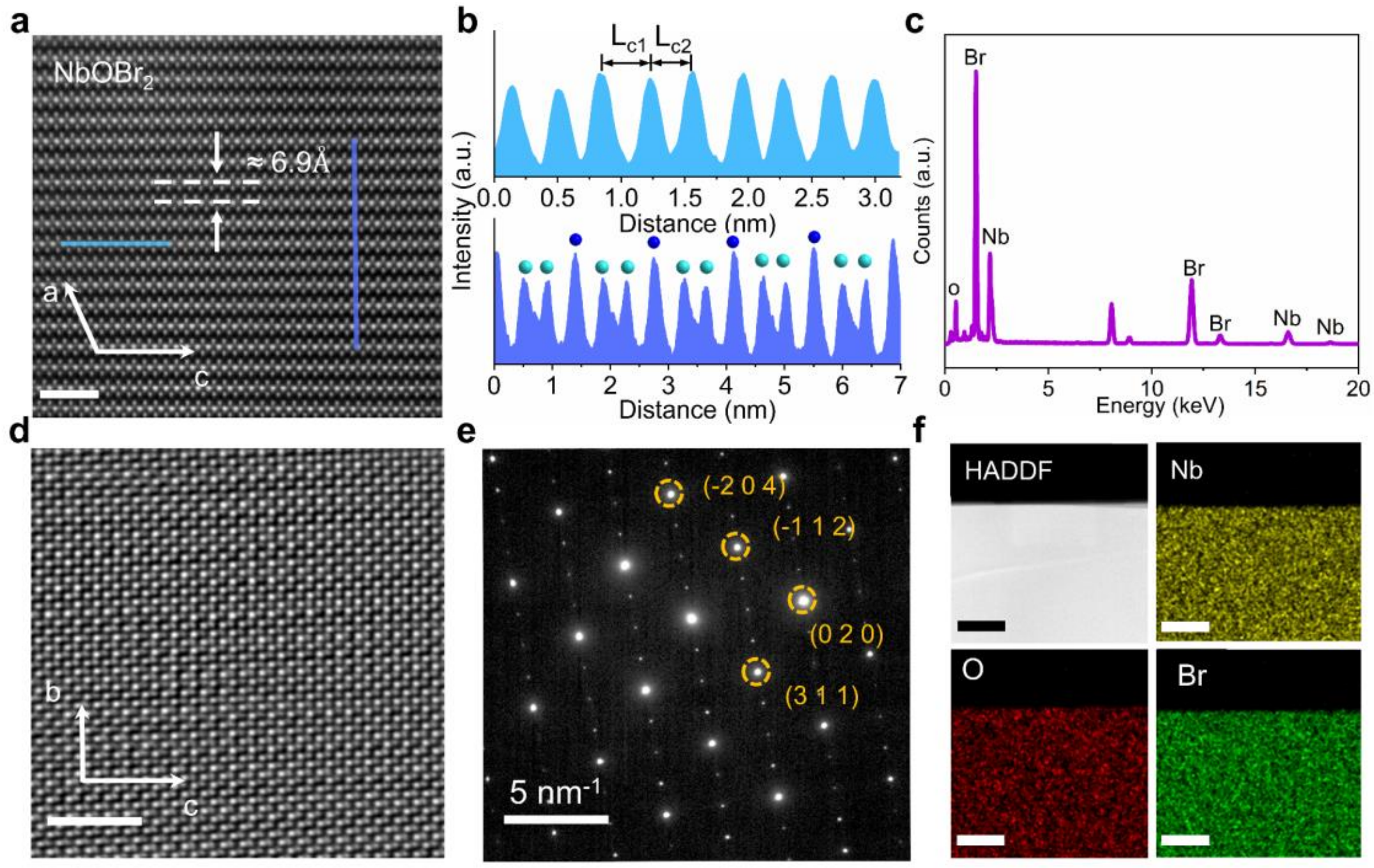

Supplementary Figure S4. Structural characterization of $NbOBr_2$. a, Cross sectional STEM image of $NbOBr_2$ viewed along the *b* axis. Scale bar: 2 nm. **b,** Line intensity profile of $NbOBr_2$ in **a**, showing the alternating unequal Nb–Nb distances(top) along the *c*-axis and ten layers thick distances(bottom). a.u., arbitrary units. **c,** The EDS spectra of $NbOBr_2$. **d,** STEM image of $NbOBr_2$ crystal viewed along the *a* axis. Scale bar: 2 nm. **e,** SAED pattern of the $NbOBr_2$ crystal. **f,** The EDS spectra of $NbOBr_2$. Scale bar: 50 nm.

High-resolution transmission electron microscopy (HRTEM) was used to investigate the crystal structure of $NbOX_2$. The HAADF-TEM images of $NbOX_2$ nanosheets transferred on the copper grid show a sharp edge, and the corresponding transmission electron microscopy energy-dispersive spectroscopy (TEM-EDS) mapping indicates that Nb, O, Br and I is uniformly distributed. SAED pattern of $NbOBr_2$ and $NbOI_2$ shows the sharp SAED spots, reflecting the high crystallinity of as-exfoliated 2D $NbOX_2$ flake. Elemental analysis by energy dispersive spectrometry (EDS) demonstrates that the $NbOX_2$ nanoplates consist only of Nb, O and X elements, with the mole ratio of Nb:O:X ≈ 1:1:2, consistent with the expected stoichiometric ratio.

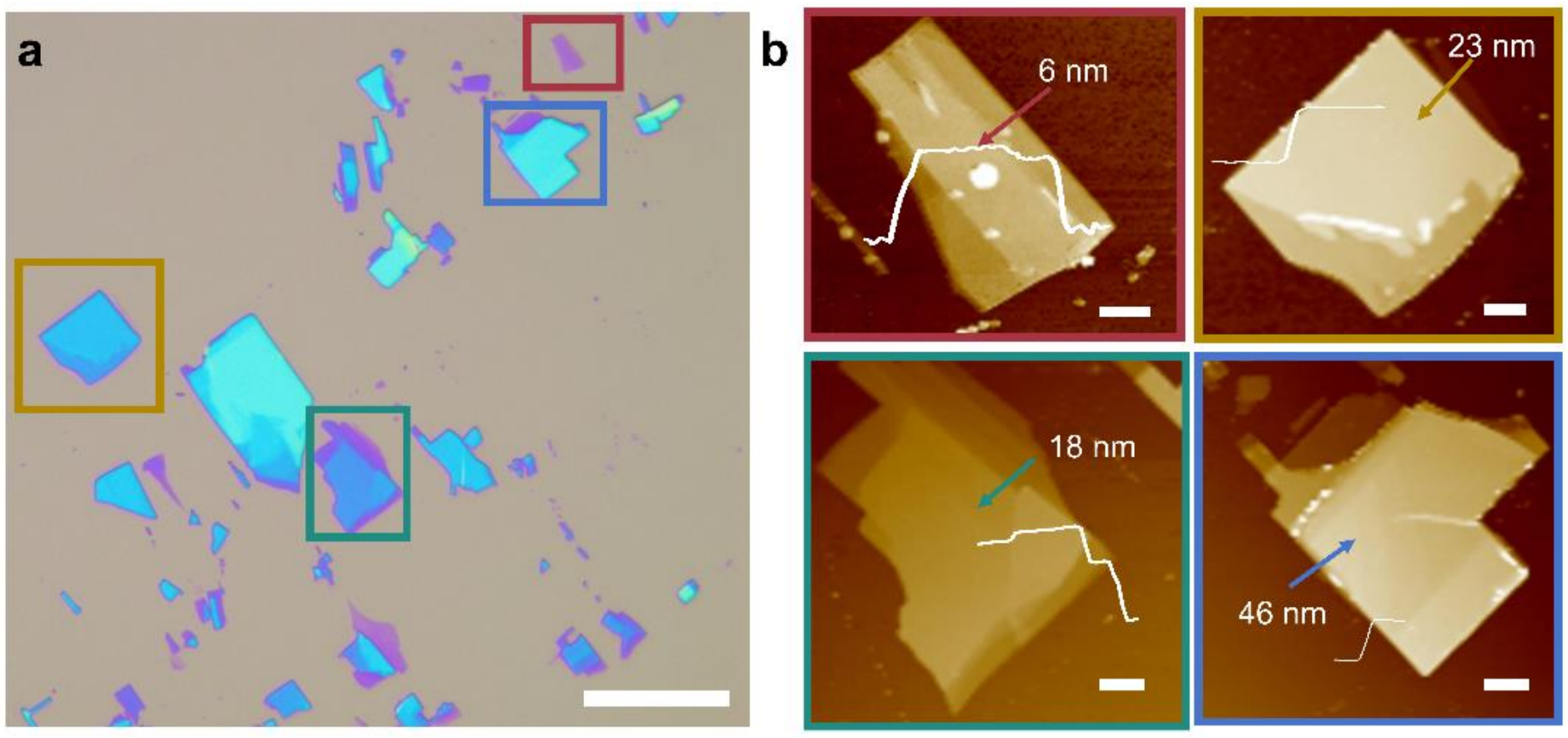

Supplementary Figure S5. OM and AFM images of exfoliated $NbOBr_2$ flake for the measurements of Raman. Scalebar: **a,** 20 μm; **b,** 2 μm.

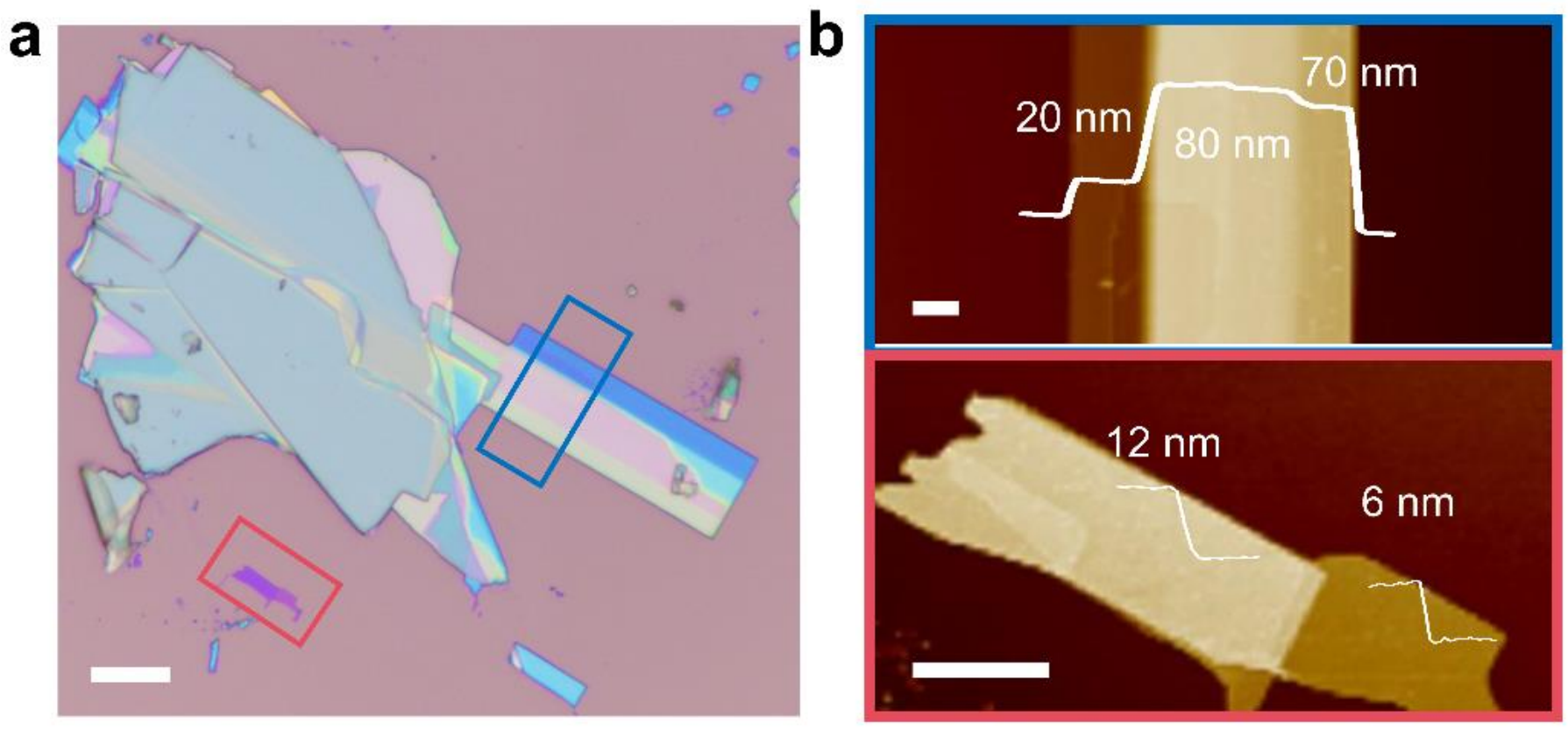

Supplementary Figure S6. OM and AFM images of exfoliated $NbOI_2$ flake for the measurements of Raman. Scalebar: **a,** 10 μm; **b,** 2 μm.

Supplementary Note 2. Lattice vibrational properties

The $NbOX_2$ crystal belongs to the $C2$ space group with eight atoms in per unit cell, supports a total of 21 Raman-active optical phonons[1,2]. These modes include $11A + 10B$, where A and B denote the irreducible representations and can be expressed as

$$\mathbf{R}(A) = \begin{bmatrix} a & 0 & d \\ 0 & b & 0 \\ d & 0 & c \end{bmatrix}, \mathbf{R}(B) = \begin{bmatrix} 0 & e & 0 \\ e & 0 & f \\ 0 & f & 0 \end{bmatrix}$$

where a, b, c, d, e, and f are tensor elements depended on the cross-section of Raman scattering. When considering the effects of light absorption, the Raman tensor is redefined as

$$\mathbf{R}(A) = \begin{bmatrix} |a|e^{i\varphi_a} & 0 & |d|e^{i\varphi_d} \\ 0 & |b|e^{i\varphi_b} & 0 \\ |d|e^{i\varphi_d} & 0 & |c|e^{i\varphi_c} \end{bmatrix}, \mathbf{R}(B) = \begin{bmatrix} 0 & |e|e^{i\varphi_e} & 0 \\ |e|e^{i\varphi_e} & 0 & |f|e^{i\varphi_f} \\ 0 & |f|e^{i\varphi_f} & 0 \end{bmatrix}$$

where φ_a is the phase of the Raman tensor element a.

Under ambient conditions, some vibration modes are not obvious, only five Raman peaks are observed in experiment. Through symmetry analysis and polarized Raman measurements (presented subsequently), the five Raman peaks was confirmed as A- or B-symmetry modes. The Raman scattering intensity (***I***) is related to the polarization of the incident light $\mathbf{e}_i$ and the scattered light $\mathbf{e}_s$ and **R**

$$I \propto |\mathrm{e}_i \cdot \mathrm{R} \cdot \mathrm{e}_s|^2 \quad (1)$$

To facilitate calculations, the polarization of the incident light is defined as $\mathbf{e}_i = (0, \sin\theta, \cos\theta)$. Under parallel polarized configuration $\mathbf{e}_s = \mathbf{e}_i = (0, \sin\theta, \cos\theta)$, while under perpendicular configuration $\mathbf{e}_s = (0, -\cos\theta, \sin\theta)$.The Raman intensity of A- and B-symmetry modes is calculated as below:

$$I_A^{\parallel} \propto |b|^2 cos^4\theta + |c|^2 sin^4\theta + \frac{1}{2}|b||c| sin^2 2\theta \cos(\varphi_c - \varphi_b) \quad (2)$$

$$I_A^{\perp} \propto \frac{1}{4} sin^2 2\theta |b| + |c|^2 - 2|b||c| cos(\varphi_c - \varphi_b) \quad (3)$$

$$I_B^{\parallel} \propto |f|^2 sin^2 2\theta \quad (4)$$

$$I_B^{\perp} \propto |f|^2 cos^2 2\theta \quad (5)$$

Obviously, the polarization-dependent Raman intensity show a period of π in the parallel configuration and a period of π/2 in the perpendicular configuration for A-

symmetry modes, while the period is π/2 in both parallel and perpendicular configurations for *B*-symmetry modes.

The polarization-dependent Raman peak intensities of the five observed modes of $NbOX_2$ are plotted below in Figure S5 and Figure S6. Specifically, P1, P2, and P3 of NbOBr are attributed to the *A*-symmetry modes, whereas P4 and P5 belong to the *B*-symmetry modes. In $NbOI_2$, all observed Raman peaks correspond to the *A*-symmetry mode All of the five modes for both materials can well fit the above formula.

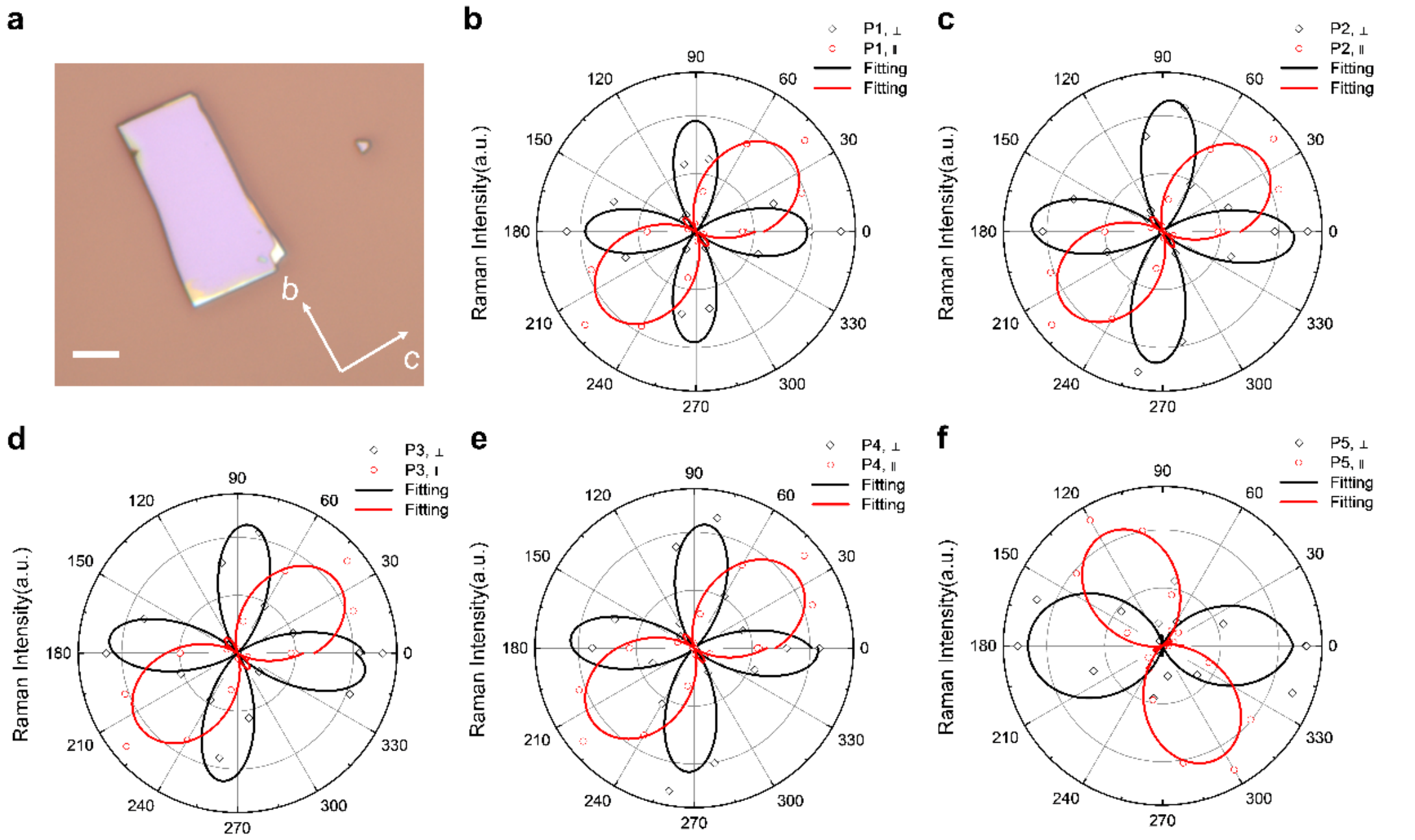

Supplementary Figure S7. **Polarized Raman spectra of $NbOBr_2$.** The optical image of the test sample is shown in **a**. The polarization-dependent Raman peak intensity of **b**, P1 (104.6 cm^{-1}); **c**, P2 (130.3 cm^{-1}); **d**, P3 (241.6 cm^{-1}); **e**, P4 (276.2 cm^{-1}); and **f**, P5 (649.7 cm^{-1}) at various polarization angles under parallel (red line) and perpendicular (black line) configuration.

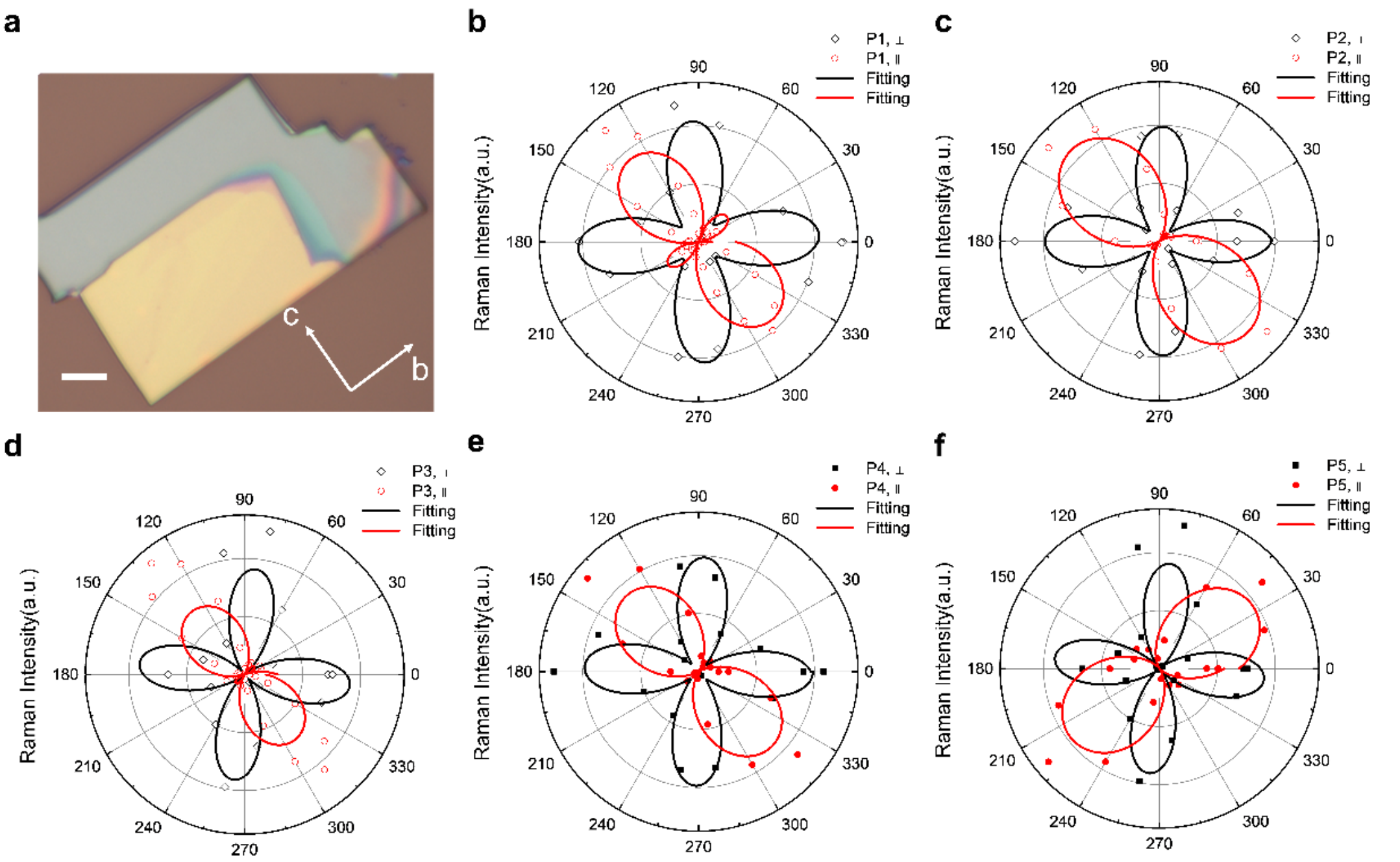

Supplementary Figure S8. **Polarized Raman spectra of $NbOI_2$.** The optical image of the test sample is shown in **a**. **b-f,** The polarization-dependent Raman peak intensity of **b**, P1 (104.1 cm^{-1}); **c**, P2 (206.3 cm^{-1}); **d**, P3 (271.1 cm^{-1}); **e**, P4 (519.3 cm^{-1}); and **f**, P5 (611.2 cm^{-1}) at various polarization angles under parallel (red line) and perpendicular (black line) configuration.

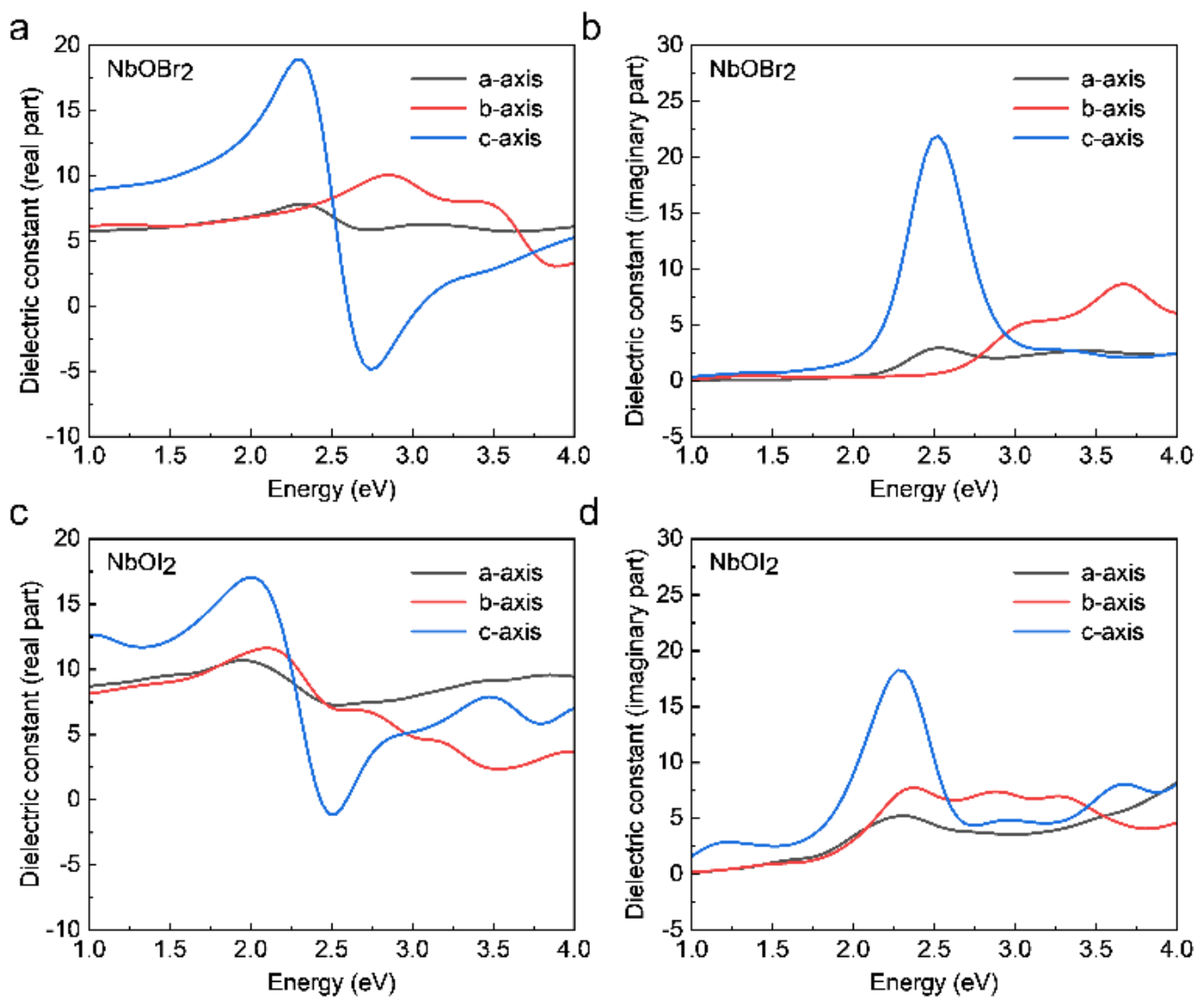

Supplementary Figure S9. Anisotropic dielectric constants of bulk $NbOBr_2$ and $NbOI_2$. (a, c) Real part and (b, d) imaginary part of dielectric constants along different crystallographic axes.

Supplementary Note 3. Stability of the $NbOX_2$ samples

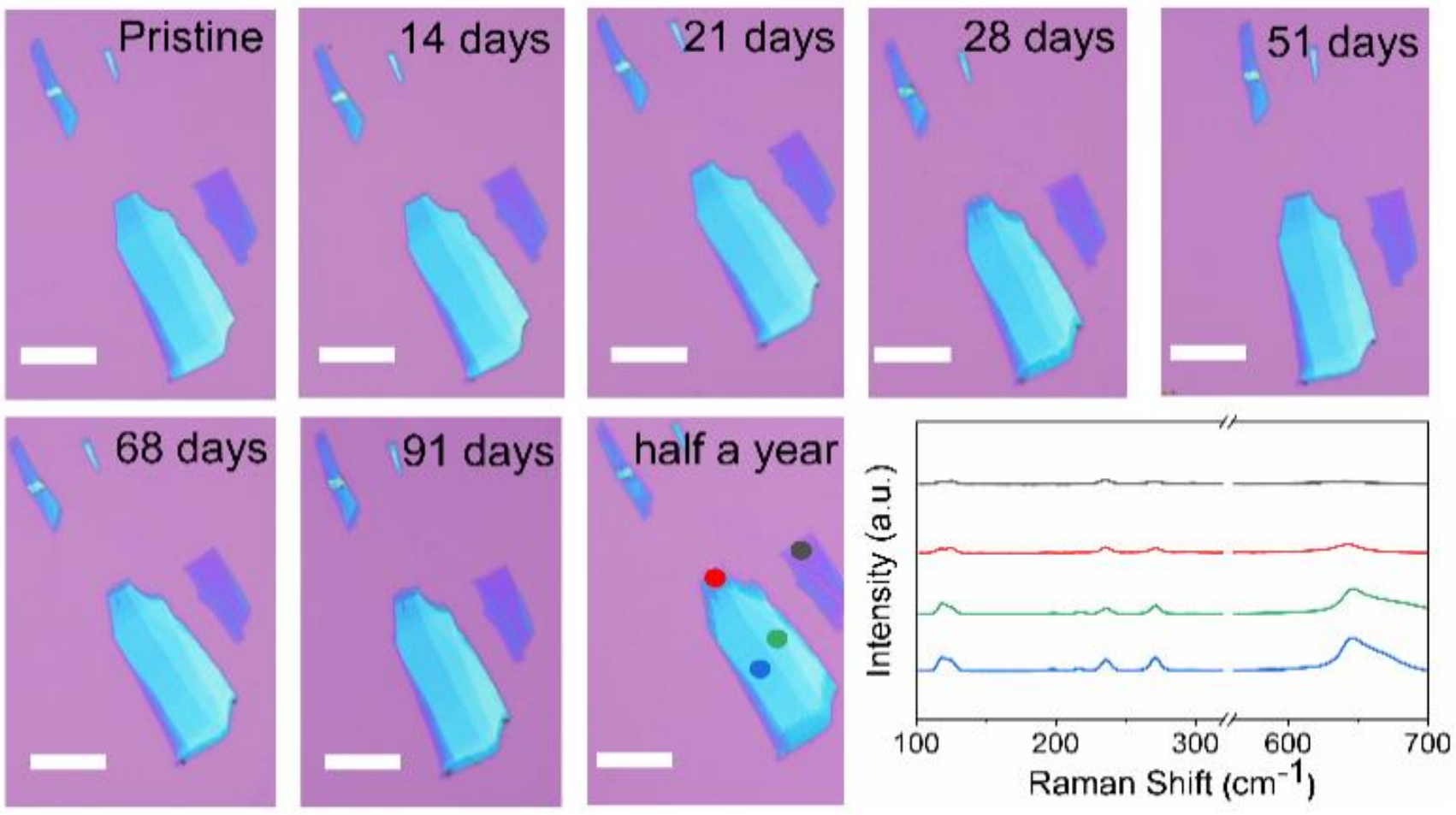

Supplementary Figure S10. Stability investigation of exfoliated $NbOBr_2$ Nano Sheets. Scale bar:20 μm. The Raman spectrum (bottom right) with certain color is take from the dot area with the same color in the left image.

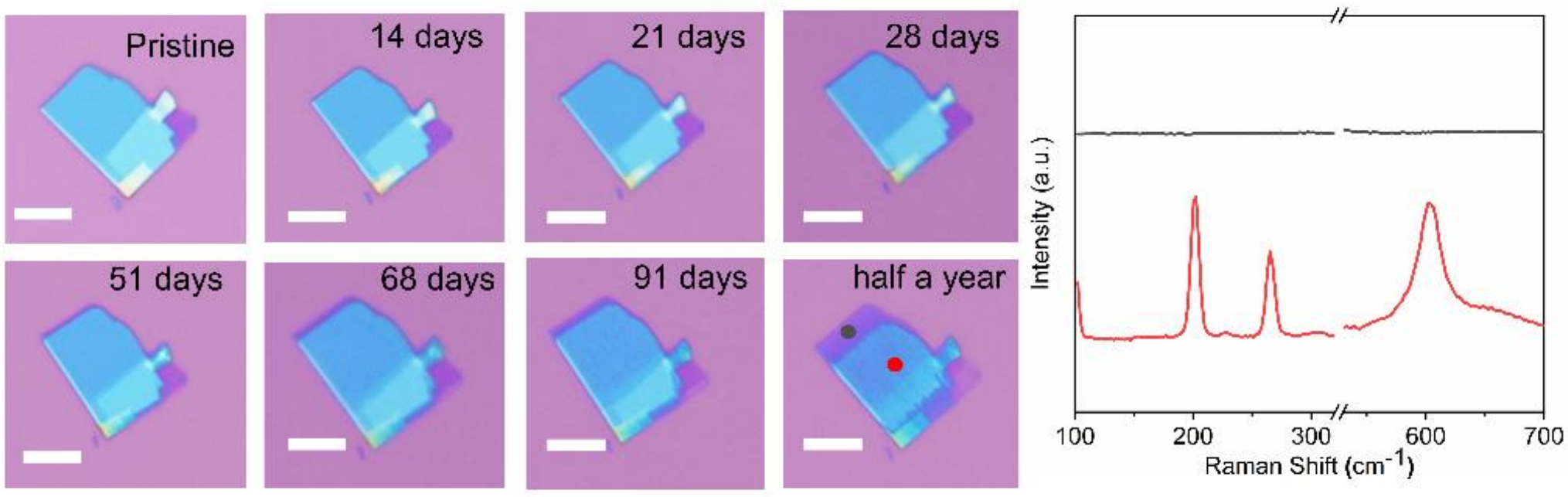

Supplementary Figure S11. Stability investigation of exfoliated $NbOI_2$ NanoSheets. Scale bar:10 μm. The Raman spectrum (bottom right)with certain color is take from the dot area with the same color in the left image.

Over time with exposure to air, $NbOX_2$ exhibited minor degradation along the flake edges, while the flake remains its initial status for the most part. No detectable changes in $NbOX_2$ were observed within the first week after exfoliation. But slight change at the edge could be noted from the second week for $NbOI_2$ and the third week for $NbOBr_2$, which gradually become obvious with time. Raman spectroscopy at different position ($NbOBr_2$ are shown on the bottom right, $NbOI_2$ are shown on the right, the Raman spectrum (bottom right)with certain color is take from the dot area with the same color

in the left image) revealed that no characteristic Raman peaks can be observed in the degraded area, whereas the signals in the central regions remain unchanged, implying that the lattice structure and crystallinity in the central was well preserved.

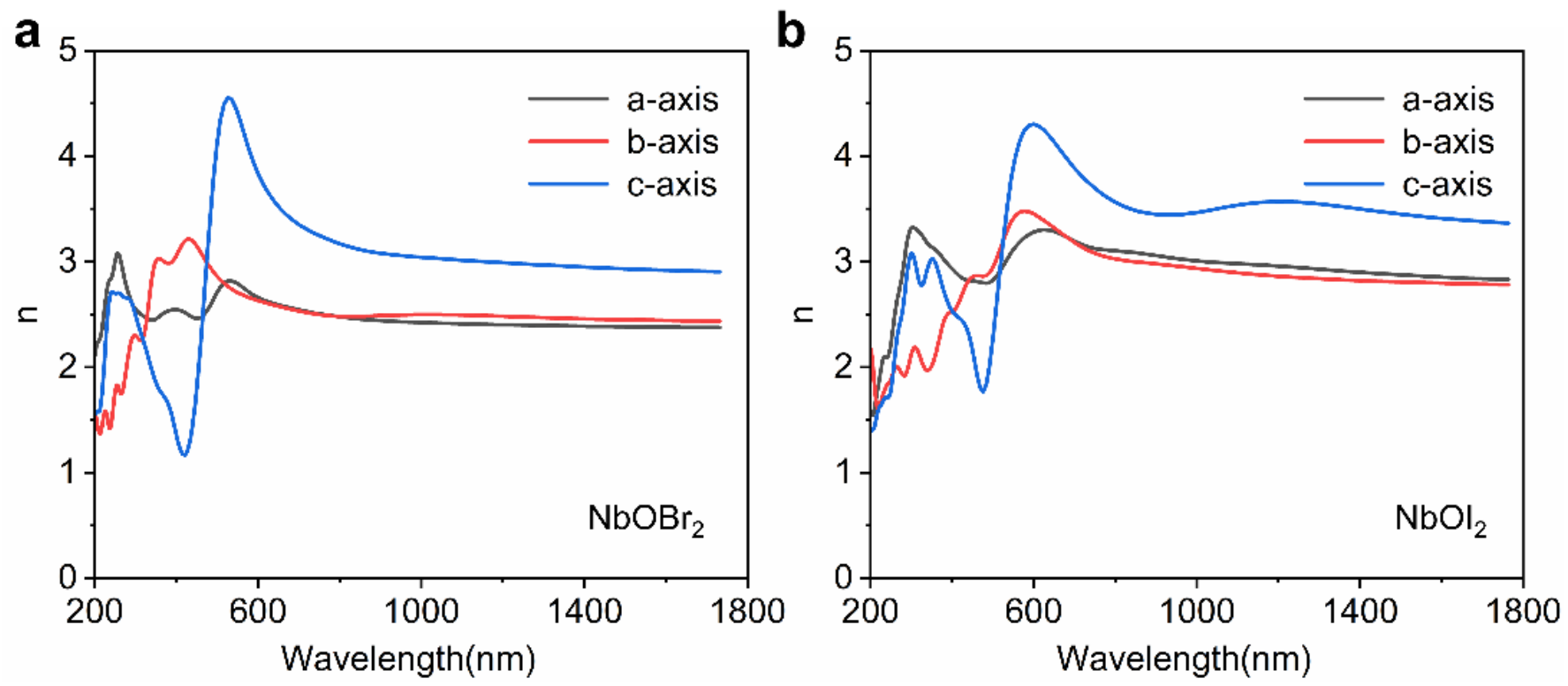

Supplementary Figure S12. **Calculated refractive index of $NbOBr_2$ and $NbOI_2$.** The theoretical refractive index n of $NbOBr_2$ **a** and $NbOI_2$ **b** along three crystallographic axes, derived from the GW-BSE calculation.

Supplementary Note 4. Calculation of the second-order nonlinear susceptibilities

In general, the power of the SHG signal follows the following relationship[2]:

$$P_{2\omega} \propto \frac{l^2 d^2 P_\omega^2}{{n_\omega}^2 n_{2\omega} {\lambda_\omega}^2}$$

where P_ω and $P_{2\omega}$ are the excitation and SH intensity, n_ω and $n_{2\omega}$ are the refractive index at the excitation wavelength and the SHG signal wavelength, l is the thickness of the sample, d is the effective second-order nonlinear coefficient, λ_ω is the excitation wavelength. To determine the second-order nonlinear coefficient of $NbOX_2$, we use a $LiNbO_3$ sample (500 nm) as a reference for comparative estimation. The d_{eff} can be obtain by considering the following relation:

$$\frac{d^2(LiNbO_3)}{d^2(NbOX_2)} = \frac{{n_\omega}^2(LiNbO_3)n_{2\omega}(LiNbO_3)}{{n_\omega}^2(NbOX_2)n_{2\omega}(NbOX_2)} \frac{l^2(NbOX_2){\lambda_\omega}^2(LiNbO_3)P_{2\omega}(NbOX_2){P_\omega}^2(LiNbO_3)}{l^2(LiNbO_3){\lambda_\omega}^2(NbOX_2)P_{2\omega}(LiNbO_3){P_\omega}^2(NbOX_2)}.$$

During the SHG experiments, the $NbOX_2$ and $LiNbO_3$ sample were measured under the same excitation conditions (λ_ω and P_1). We take d_{eff} = 40 pm/V as the reference value[3]. The refractive index dispersion data in Figure. 2c-d was employed.

Supplementary Note 5. Polarization-dependent SHG

The second-order nonlinear coefficient matrix of $NbOX_2$ crystal can be expressed as[2]:

$$\begin{bmatrix} 0 & 0 & 0 & \chi_{abc}^{(2)} & 0 & \chi_{aab}^{(2)} \\ \chi_{baa}^{(2)} & \chi_{bbb}^{(2)} & \chi_{bcc}^{(2)} & 0 & \chi_{bac}^{(2)} & 0 \\ 0 & 0 & 0 & \chi_{cbc}^{(2)} & 0 & \chi_{cab}^{(2)} \end{bmatrix}.$$

Therefore, when the propagation of pump is along *a*-axis, under the paraxial approximation, we only consider the polarization components of the pump light along the *b*- and *c*-axis, the SHG polarization along *b*- and *c*- axis are:

$$P_b(2\omega) = E_b^2\chi_{bbb}^2 + E_c^2\chi_{bcc}^2 \quad (6)$$

$$P_c(2\omega) = 2E_bE_c\chi_{cbc}^2 \quad (7)$$

Transform the above crystallographic axes (a, b, c) to the laboratory axes (x, y, z) we have:

$$P_y(2\omega) = E_b^2\chi_{bbb}^2 \cdot cos\theta + E_c^2\chi_{bcc}^2 \cdot cos\theta - 2E_bE_c\chi_{cbc}^2 \cdot sin\theta \quad (8)$$

$$P_z(2\omega) = E_b^2\chi_{bbb}^2 \cdot sin\theta + E_c^2\chi_{bcc}^2 \cdot sin\theta + 2E_bE_c\chi_{cbc}^2 \cdot cos\theta \quad (9)$$

where $E_b = E_\omega \cdot cos\theta$ and $E_c = E_\omega \cdot sin\theta$ (set the polarization of fundamental wave along *y*-axis).

Therefore, the polarization component perpendicular to the pump is calculated as $I_{2\omega}^{\perp} = \left|P_y(2\omega)\right|^2$, while the polarization component parallel to the pump is calculated as $I_{2\omega}^{\parallel} = |P_z(2\omega)|^2$. Substitute all the previous equations:

$$I_{2\omega}^{\perp} \propto cos^2\theta \cdot [\chi_{bcc}^{(2)} + sin^2\theta\left(\chi_{bbb}^{(2)} - \chi_{bcc}^{(2)} - 2\chi_{cbc}^{(2)}\right)]^2 \quad (10)$$

$$I_{2\omega}^{\parallel} \propto sin^2\theta \cdot [\chi_{bbb}^{(2)} + cos^2\theta\left(\chi_{bcc}^{(2)} - \chi_{bbb}^{(2)} + 2\chi_{cbc}^{(2)}\right)]^2 \quad (11)$$

Therefore, after fitting these two equations with the experimental data, the relative strength of these three effective matrix elements is calculated as

NbOBr2: $\chi_{bbb}^{(2)} \approx 210, \chi_{bcc}^{(2)} \approx 100, \chi_{cbc}^{(2)} \approx 9.5$

(corresponding to normalized relative strength, not the real strength).

Thus $\chi_{bbb}^{(2)}/\chi_{bcc}^{(2)} \approx 2.10,\ \chi_{cbc}^{(2)}/\chi_{bcc}^{(2)} \approx 0.10$

$NbOI_2$: $\chi_{bbb}^{(2)} \approx 339.94, \chi_{bcc}^{(2)} \approx 218.20, \chi_{cbc}^{(2)} \approx 28.46$

(corresponding to normalized relative strength, not the real strength).

Thus $\chi^{(2)}_{bbb}/\chi^{(2)}_{bcc} \approx 1.56$, $\chi^{(2)}_{cbc}/\chi^{(2)}_{bcc} \approx 0.13$

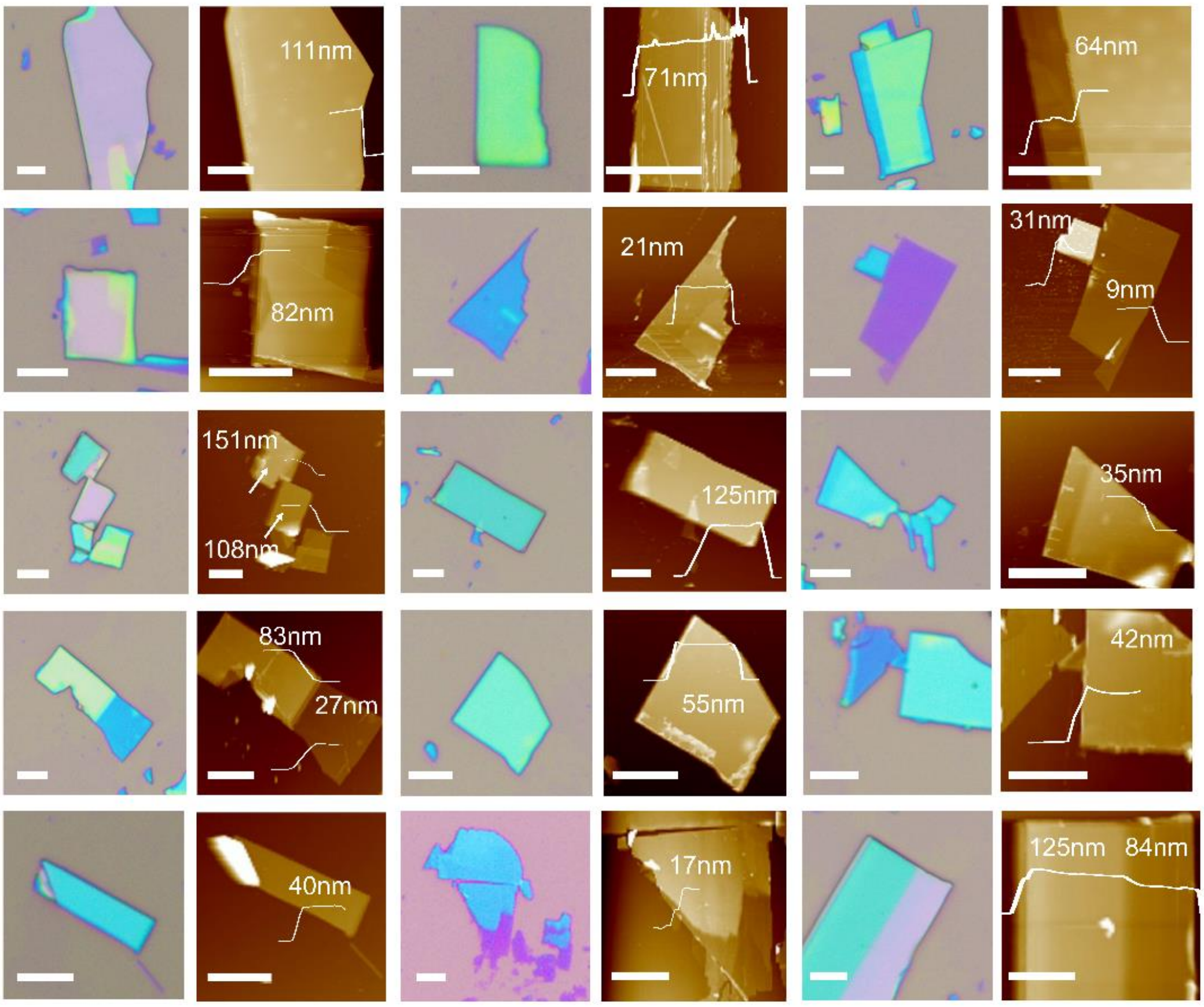

Supplementary Figure S13. OM and AFM images of exfoliated $NbOBr_2$ flake for the thickness-dependent SHG measurements in reflection mode. Scalebar: 5 µm.

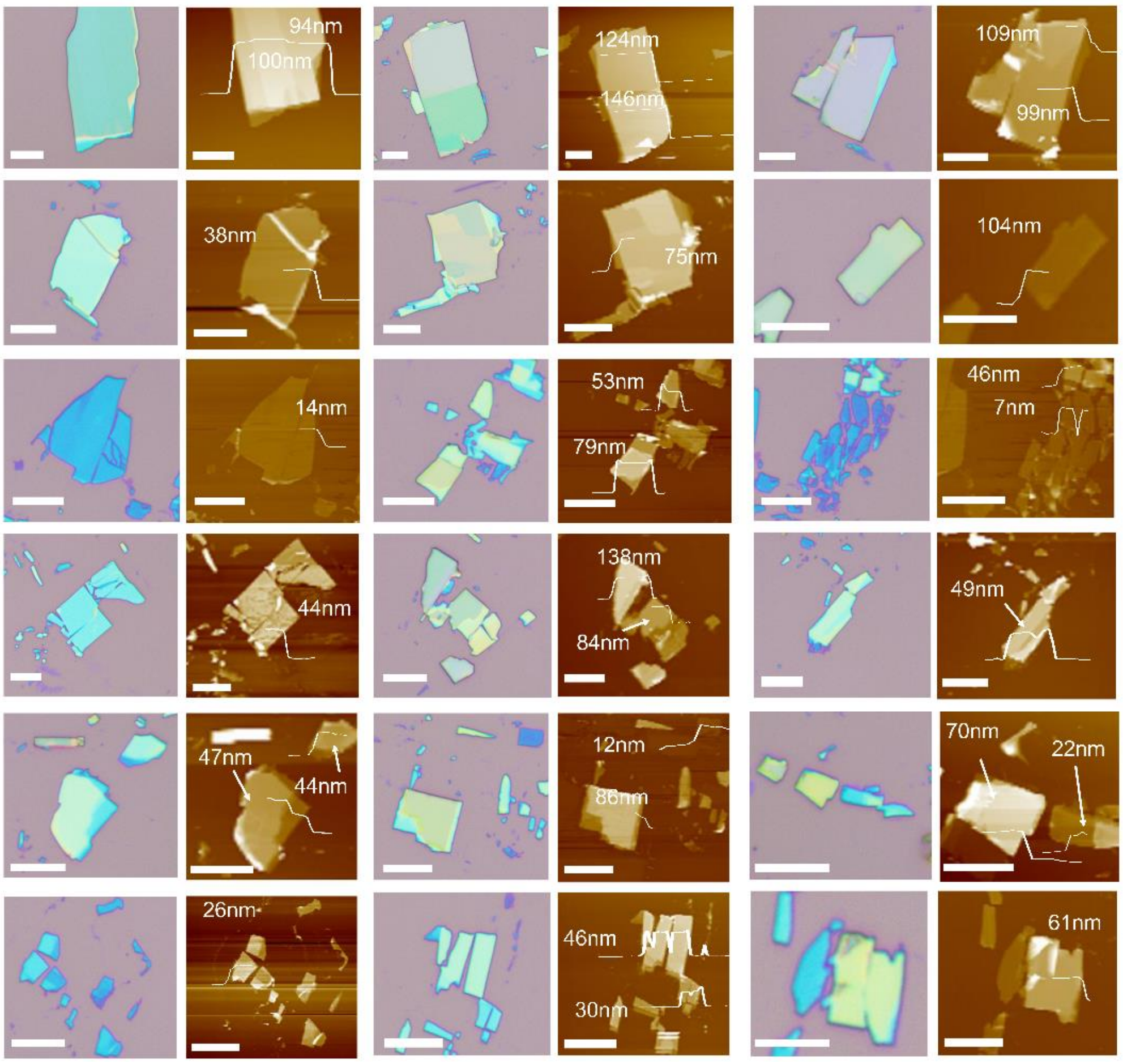

Supplementary Figure S14. OM and AFM images of exfoliated $NbOI_2$ flake for the thickness-dependent SHG measurements in reflection mode. Scalebar: 10 µm.

Supplementary Note 6. Calculation of coherence length

Here we calculate the coherence length of the main contribution element $\chi^{(2)}_{bbb}$-based SPDC process pumped by 457 nm under transmission configuration, using the refractive index dispersion data in Figure. 2c-d. According to the equation $\Delta L = \frac{\lambda_\omega}{4|n_\omega - n_{2\omega}|}$, where ΔL is the coherence length, and n_ω and $n_{2\omega}$ is the refractive index at wavelength 914 nm and 457 nm, respectively. Therefore, the calculated coherence length for $NbOBr_2$ is 2.451 μm, and for $NbOI_2$ is 775 nm. Figure. S14 shows the experimental thickness-dependent SHG intensity for $NbOBr_2$ and $NbOI_2$ under transmission configuration. The measured thicknesses were all below the coherence length and thus exhibit a monotonically increasing dependency relationship in both two materials. The lines were fitted according to the calculated coherence length and agree well with the experimental data. Figure. S15 gives an intuitive diagrammatic sketch of dependence of the normalized efficiency on the thickness.

Using the same calculation method, the coherence lengths of $NbOBr_2$(60 nm) and $NbOI_2$ (52 nm) were determined under 1030 nm pumping in a back-reflection geometry. Nevertheless, the actual coherence length should account for the effects of interfacial nonlinear contributions[2].

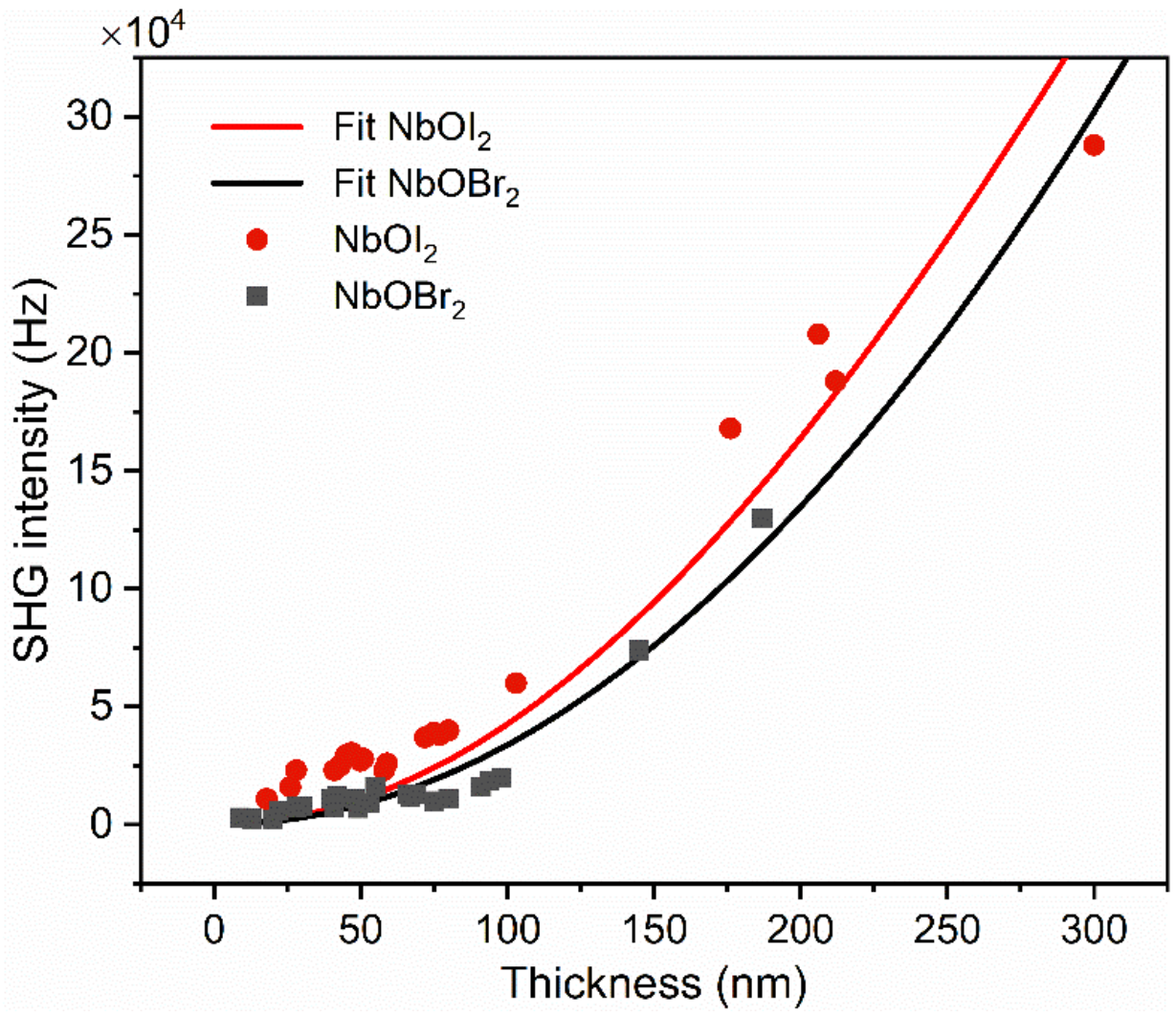

Supplementary Figure S15. Measured thickness-dependent SHG intensity for $NbOBr_2$ and $NbOI_2$ under transmission configuration.

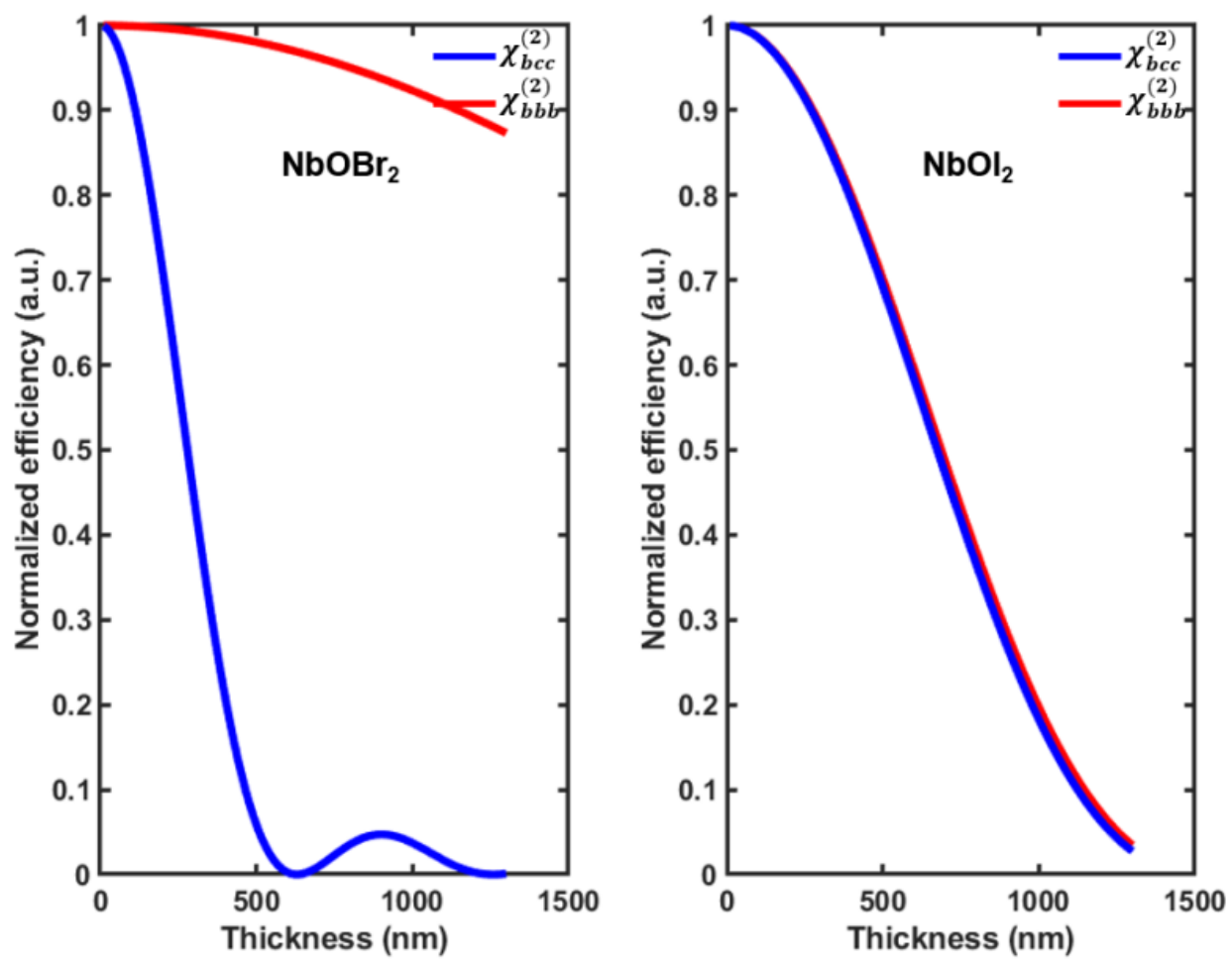

Supplementary Figure S16. Normalized relative efficiency of the SPDC process ($1/\chi^{(2)} \cdot 1/l^2$) varies with the thickness of $NbOBr_2$ and $NbOI_2$ crystal. The two curves for $NbOI_2$ nearly overlap with each other.

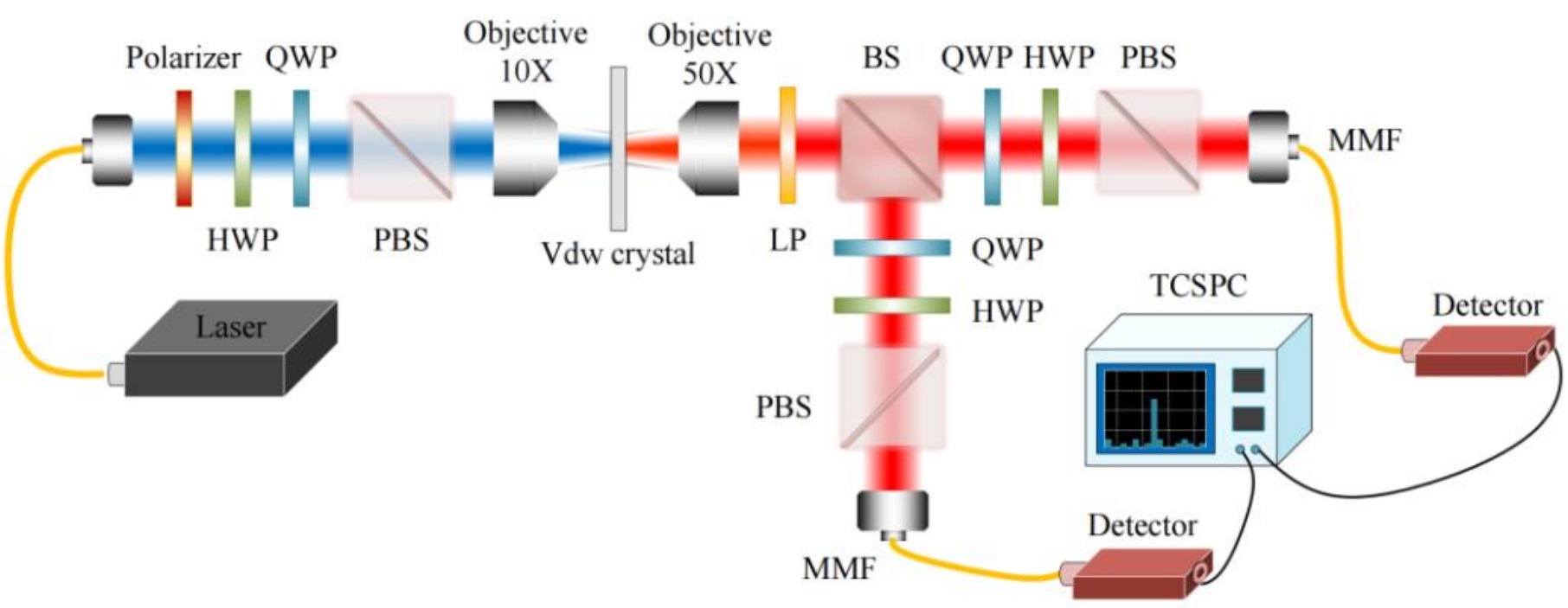

Supplementary Figure S17. Experimental setup for SPDC·measurements. Schematic diagram of the optical setup for measuring polarization-resolved second-order correlation function $g^{(2)}$ and quantum state tomography of SPDC photons. The setup for SPDC spectrum measurement is the same, but with a multimode fiber of 300 m length added before each single-photon detector. Introduction of abbreviation: HWP-half wave plate; QWP-quart wave plate; BS-beam splitter; PBS-polarization beam splitter; LP-long pass filter; MMF-multi-mode fiber; TCSPC- time-correlated single photon counting.

The whole detection efficiency for coincidence rates measurements with our system is estimated approximately as 0.26%, including the transmission through objective of twin photons $\eta_{i,s}^{Obj} = 0.7^2$, the efficiency of beam splitter $\eta_{i,s}^{BS} = 0.5 \times 0.98^2 = 0.48$, the

coupling efficiency of the fibers $\eta_{i,s}^{Fibre} = 0.72 \times 0.70$, and the intrinsic detection efficiency of the detectors as 914 nm wavelength $\eta_{i,s}^{SPD} = 0.38^2$. Considering the around 6-fold enhancement of the coincidence rate with 800 nm long-pass filter comparing to that with 900 nm, the efficiency of the filter is calculated as $\eta_{i,s}^{Filter} = 0.95^2 \times \frac{1}{6} = 0.15$.

Supplementary Note 7. Polarization state of the two-photons from $NbOI_2$

According to the relative strength of three effective matrix elements, the process induced by $\chi_{cbc}^{(2)}(\chi_{ccb}^{(2)})$ is neglected for simplicity. And according to the similar phase mismatch of nonlinear process of $NbOI_2$ induced by $\chi_{bbb}^{(2)}$ and $\chi_{bcc}^{(2)}$, shown in Figure. S15, the ratio of the generated twin photons polarized along *b*-axis to the counterpart along *c*-axis under *b*-polarized pump should roughly equal to the ratio $\chi_{bbb}^{(2)}/\chi_{bcc}^{(2)}$. We define the polarization along *b*-axis as $|H\rangle$ state and the polarization along *c*-axis as $|V\rangle$ state. Consequence, the expected quantum state of the generated SPDC photon pairs from $NbOI_2$ under H-polarized pump is $\frac{1}{\sqrt{1.56^2+1}}(1.56 \cdot |HH\rangle + 1 \cdot |VV\rangle) = 0.84|HH\rangle + 0.54|VV\rangle$.

Supplementary Note 8. Quantum state tomography

The experimental density matrix $\hat{\rho}$ of the two photon polarization state was reconstructed by the quantum state tomography method. Considering the density matrix $\hat{\rho}$ is positive semidefinite and Hermitian, there is total d^2 real variables, where d represent the dimension of the matrix. Therefore, for a *2*-qubits state in our work, the dimension of the density matrix is $2^2 = 4$, and the corresponding amount of real variables is 16. The polarized states $\{H, V, D, R\}^2$ were chosen as the measured polarization projection basis in experiments. Here $|H\rangle$ is the horizontal linearly polarization, $|V\rangle$ is the vertical linearly polarization, $|D\rangle = (|H\rangle + |V\rangle)/\sqrt{2}$

and $|R\rangle = (|H\rangle + i|V\rangle)/\sqrt{2}$, respectively. A set of orthonormal basis vectors of the two-photon polarization state can be formed through these 16 measured operators. And thus we have gotten 16 measured results to solve the 16 real variables and further reconstruct the measured parameterized density matrix. Considering the experimental noise, the maximum likelihood estimation method is used to solve the reconstruction equations[4,5]

Fidelity is calculated as: $F(\rho_{exp}, \rho_{thero}) = tr(\rho_{experiment} \cdot \rho_{theory})$[6].

Concurrence is calculated according to the method in Ref. 4. First, construct the spin-flipped density matrix $\tilde{\rho} = (\sigma_y \otimes \sigma_y)\rho^*(\sigma_y \otimes \sigma_y)$ (σ_y is the Pauli-y operator) and the Hermitian operator $R = \sqrt{\sqrt{\rho}\tilde{\rho}\sqrt{\rho}}$. The concurrence is then given by $C = \max\{0, \sqrt{\lambda_1} - \sqrt{\lambda_2} - \sqrt{\lambda_3} - \sqrt{\lambda_4}\}$, where $(\lambda_1, \lambda_2, \lambda_3, \lambda_4)$ is the eigenvalues of R arranged in decreasing order.

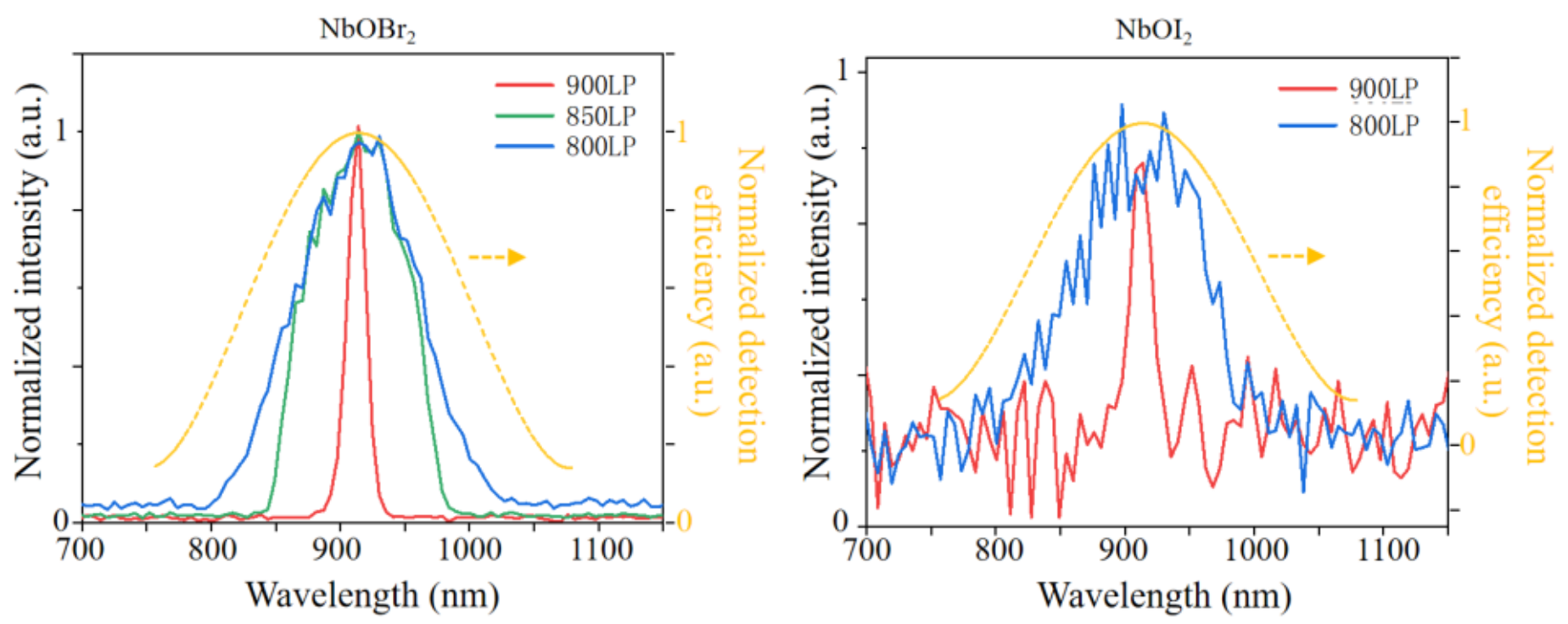

Supplementary Figure S18. Experimental spectrum of SPDC photons from $NbOBr_2$ and $NbOI_2$. The spectrum was reconstructed through temporal correlation measurements leveraging fiber dispersion as the method in Ref. 7 Different color cuve was obtained with different wavelength. LP-long pass filter.

The yellow curves represent the wavelength-dependent normalized detection efficiency of the photon detectors in our experiments. Therefore, the accurate bandwidths of the SPDC photons from both materials are supposed to be broader than the measured ones shown here.

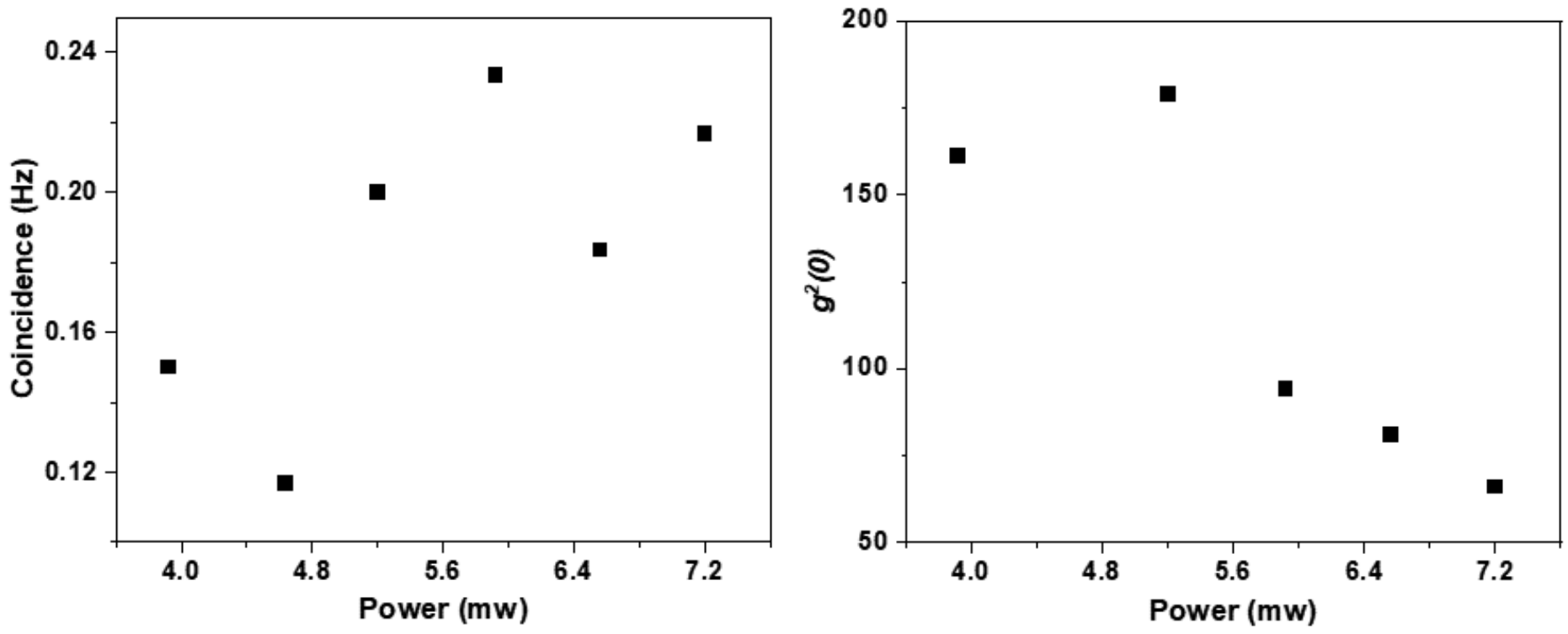

Supplementary Figure S19. Near-infrared SPDC sources based on $NbOBr_2$ (thickness ~100 nm) under pump at 655 nm wavelength. In measurements of Figure. 4, multimode fibers were used to collect the SPDC photons pumped by 457 nm wavelength, here the generated SPDC photons were coupled into single mode fibers and thus exhibit much lower collection efficiencies. Typical bunching effect of photon pairs have been observed in both $NbOBr_2$ and $NbOI_2$, demonstrating the SPDC process occurs. $NbOI_2$ shows an obviously higher photon pairs, and the power-dependent coincidence rate and $g^{(2)}(0)$ both consist with theory (see the inset of Figure 4). While the photon pairs from $NbOBr_2$ are so weak that leading significant errors.

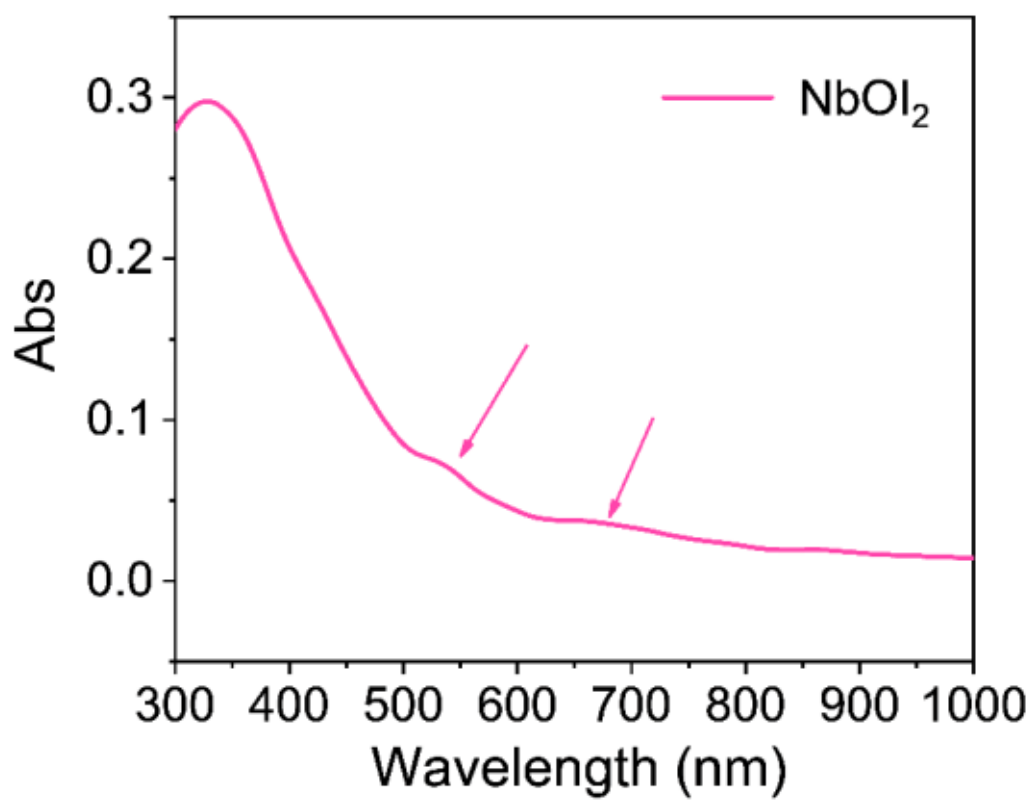

Supplementary Figure S20. Absorption spectrum of $NbOI_2$ measured by differential transmission method. Two weak absorption peaks of $NbOI_2$ at approximately 550 nm and 700 nm were observed.

Supplementary Table 1 Comparison of different materials for SPDC sources.

Materials	Wavelength (nm)	$\chi^{(2)}$ [pm/V]	Thickness (nm)	Brightness (Hz/mW)	Normalized Brightness (Hz/(mW•nm))	Normalized Brightness (Hz/(mW•nm^2))	Reference
PPLN	1560	40	4×10^{7}	3×10^{5}	0.03	7.5×10^{-10}	[8]
PPKTP	1569	10.7	1×10^{7}	2.1×10^{4}	2.1×10^{-3}	2.1×10^{-10}	[9]
TFLN	1570	40	304	0.021	7.0×10^{-5}	2.3×10^{-7}	[10]
BBO	702.2	3.9	2×10^{6}	900	4.5×10^{-4}	2.25×10^{-10}	[11]
LN film	1375	40	300	0.31	1.02×10^{-3}	3.4×10^{-6}	[7]
GaP film	1375	37	400	0.022	5.6×10^{-5}	1.4×10^{-7}	[7,12]
rBN	918	40	1.47×10^{3}	8	5.4×10^{-3}	3.7×10^{-6}	[13]
	810	40	910	9.15	0.01	1.1×10^{-5}	[14]
3R-WS_2	914	/	350	1.71	4.9×10^{-3}	1.4×10^{-5}	[15]
3R-MoS_2	1576	30	285	8.0×10^{-3}	2.8×10^{-5}	9.8×10^{-8}	[16]
$NbOCl_2$	808	200	150	1.46	9.8×10^{-3}	6.5×10^{-5}	[2]
Twisted $NbOCl_2$	818	200	1.2×10^{3}	0.57	4.68×10^{-5}	3.9×10^{-7}	[17]
$NbOBr_2$	914	232	145	1.5	1.03×10^{-2}	7.1×10^{-5}	Our work
	900	227	/	/		/	
	1300	98	100	0.04	4×10^{-4}	4×10^{-6}	
	1400	200	/	/		/	
$NbOI_2$	914	234	150	0.9	6×10^{-3}	4×10^{-5}	Our work
	1100	329	/	/		/	
	1300	252	100	0.13	1.3×10^{-3}	1.3×10^{-5}	
	1400	337	/	/		/	

Supplementary References

1 Chen, W. *et al.* Extraordinary Enhancement of Nonlinear Optical Interaction in $NbOBr_2$ Microcavities. *Advanced Materials* **36**, 2400858, doi:10.1002/adma.202400858 (2024).

2 Guo, Q. *et al.* Ultrathin quantum light source with van der Waals $NbOCl_2$ crystal. *Nature* **613**, 53-59, doi:10.1038/s41586-022-05393-7 (2023).

3 Okoth, C., Cavanna, A., Santiago-Cruz, T. & Chekhova, M. V. Microscale Generation of Entangled Photons without Momentum Conservation. *Physical Review Letters* **123**, 263602, doi:10.1103/PhysRevLett.123.263602 (2019).

4 James, D. F. V., Kwiat, P. G., Munro, W. J. & White, A. G. Measurement of qubits. *Physical Review A* **64**, 052312, doi:10.1103/PhysRevA.64.052312 (2001).

5 Banaszek, K., D'Ariano, G. M., Paris, M. G. A. & Sacchi, M. F. Maximum-likelihood estimation of the density matrix. *Physical Review A* **61**, 010304, doi:10.1103/PhysRevA.61.010304 (1999).

6 D. F. Walls , G. J. M. *Quantum Optics*. Second Edition 2008 edn, (Springer, 2010).

7 Santiago-Cruz, T., Sultanov, V., Zhang, H., Krivitsky, L. A. & Chekhova, M. V. Entangled photons from subwavelength nonlinear films. *Optics Letters* **46**, 653-656, doi:10.1364/ol.411176 (2021).

8 Bienvenu Ndagano, R. S., Lewis Wright, Krish Pandiyan, Sarah McCarthy, Ali Anwar, Corin Gawith, Loyd J. McKnight. A Gigahertz-Rate Source of Polarization-Entangled Photons Using Type-0 PPLN Waveguides. *CLEO: Applications and Technology*, 1-2 (2024).

9 Liu, Y.-C. *et al.* Observation of frequency-uncorrelated photon pairs generated by counter-propagating spontaneous parametric down-conversion. *Scientific Reports* **11**, 12628, doi:10.1038/s41598-021-92141-y (2021).

10 Zhang, J. H. *et al.* Spatially entangled photon pairs from lithium niobate nonlocal metasurfaces. *Science Advances* **8**, eabq4240,

doi:10.1126/sciadv.abq4240 (2022).

11 Kurtsiefer, C., Oberparleiter, M. & Weinfurter, H. High-efficiency entangled photon pair collection in type-II parametric fluorescence. *Physical Review A* **64**, 023802, doi:10.1103/PhysRevA.64.023802 (2001).

12 Shoji, I., Kondo, T., Kitamoto, A., Shirane, M. & Ito, R. Absolute scale of second-order nonlinear-optical coefficients. *Journal of the Optical Society of America B* **14**, 2268-2294, doi:10.1364/JOSAB.14.002268 (1997).

13 Lyu, X. *et al.* A tunable entangled photon-pair source based on a Van der Waals insulator. *Nature Communications* **16**, 1899, doi:10.1038/s41467-025-56436-2 (2025).

14 Liang, H. D. *et al.* Tunable polarization entangled photon-pair source in rhombohedral boron nitride. *Science Advances* **11**, 3710, doi:10.1126/sciadv.adt3710 (2025).

15 Feng, J. *et al.* Polarization-entangled photon-pair source with van der Waals 3R-WS_2 crystal. *eLight* **4**, 16, doi:10.1186/s43593-024-00074-6 (2024).

16 Weissflog, M. A. *et al.* A tunable transition metal dichalcogenide entangled photon-pair source. *Nature Communications* **15**, 7600, doi:10.1038/s41467-024-51843-3 (2024).

17 Kallioniemi, L. *et al.* Van der Waals engineering for quantum-entangled photon generation. *Nature Photonics* **19**, 142-148, doi:10.1038/s41566-024-01545-5 (2025).
