# Supplementary material for: Nonlinear quantum light source with van der Waals ferroelectric NbOX2 (X = Br, I)
Source: Nat Commun. 2026 Jun 30;17:8148. doi: 10.1038/s41467-026-75018-4 (PMC13458526; doi:10.1038/s41467-026-75018-4)
Supplement: Supplementary file 2 [file 41467_2026_75018_MOESM2_ESM.pdf]

nature portfolio

Peer Review File

Nonlinear quantum light source with van der Waals ferroelectric NbOX2 (X=Br, I)

Corresponding Author: Professor Xidong Duan

This file contains all reviewer reports in order by version, followed by all author rebuttals in order by version.

Attachments originally included by the reviewers as part of their assessment can be found at the end of this file.

Version 0:

Reviewer comments:

Reviewer #1

(Remarks to the Author)
Review Report
Manuscript： NCOMMS-25-83581-T
Manuscript Title: Novel nonlinear quantum light source with van der Waals ferroelectric NbOX2 (X=Br, I)
General Assessment:
This manuscript presents a systematic and valuable investigation into the second-order nonlinear optical properties and spontaneous parametric down-conversion (SPDC) capabilities of two-dimensional (2D) materials $NbOBr_2$ and $NbOI_2$, expanding the established $NbOCl_2$-based quantum source platform. The work is well-structured, experimentally sound, and provides new candidates for integrated quantum photonic sources. The findings, particularly the demonstration of polarization entanglement from $NbOI_2$, are significant for the field. However, several aspects require clarification, expansion, or correction to enhance the manuscript's impact, rigor, and readability for a broad audience.
Major Comments:
1. The abstract and introduction effectively position the work as adding new candidates to the SPDC material library. However, a more explicit discussion of the practical benefits and advantages of these $NbOX_2$ materials compared to current rapid or established material systems (e.g., PPLN, PPKTP waveguides, other 2D materials) is needed. While the Supplementary Information Table S1 is a good start, the main text should highlight key comparative metrics such as nonlinear strength per unit thickness, birefringence, operational wavelength range (especially near-infrared), and intrinsic integration advantages. Specifically addressing the question "Why choose $NbOI_2$ over a mature PPLN waveguide for a specific application?" would greatly strengthen the manuscript's impact.
2. The manuscript would benefit from a more seamless logical link between sections, particularly when transitioning from fundamental property characterization (crystal structure, refractive indices, SHG) to the final SPDC and entanglement results. For instance, the statement about $NbOI_2$'s suitability for near-infrared SPDC and 1310 nm operation should be more directly supported by the refractive index data or nonlinear susceptibility predictions extended to those wavelengths (see point 5 below).
3. Experimental Data Presentation and Rigor:
o Error Analysis: A significant omission is the lack of error bars on experimental data points in many figures (e.g., SHG intensity vs. thickness, power-dependent counts). Including error estimates is crucial for assessing the reliability of the fitted slopes (e.g., 2.09, 1.94 for quadratic dependence) and the significance of the observed trends.
o Units and Notation: Please adhere strictly to scientific notation conventions. Ensure a space between numbers and units (e.g., "1030 nm" not "1030nm"). Standardize abbreviations (e.g., "NLO" should be defined at first use and used consistently, or spelled out uniformly if preferred).
Specific Comments and Questions:
4. Introduction and Background:
o Line 74: When mentioning on-chip SPDC sources, consider broadening the example beyond PPLN to include other prominent platforms like periodically poled potassium titanyl phosphate (PPKTP) waveguides for a more comprehensive overview.
o Line 92 & Throughout: Standardize the use of "NLO." Define "nonlinear optical (NLO)" at its first occurrence and use the abbreviation thereafter, or avoid it in favor of the full term for consistency.
o 2D Materials Context: For a general audience, a brief, clear definition of 2D materials and their overarching advantages

early in the introduction would be very helpful.
5. Figure 2 – Refractive Index Dispersion: The experimental data in Figures 2c and 2d are shown only up to ~1050 nm. Given the highlighted potential for operation at 1310 nm (a key telecom wavelength), it is important to extend the experimental measurement or reliable theoretical extrapolation to 1310 nm, or based on the available dispersion model.
6. Figure 2 – Birefringence Comparison: The claim of large birefringence compared to "most conventional birefringent materials" is noted. To strengthen this, a brief quantitative comparison with other thin-film or integrated photonic material platforms would be informative. Clearly state the advantage of such high birefringence for phase-matching in nonlinear processes like SPDC.
7. Figure 3 – SHG Analysis and Efficiency:
o The SHG intensity plots require units on the y-axis.
o Following the methodology in Supplementary Section 4, please provide an estimated second-order nonlinear susceptibility ($\chi^{(2)}$) or effective nonlinear coefficient (d_eff) for both materials at the measured wavelengths. A direct comparison of these calculated values with those of common commercial bulk (e.g., BBO, $LiNbO_3$) and integrated (e.g., PPLN, PPKTP) materials, perhaps in an expanded table, would be extremely valuable for assessing their practical potential.
8. Figure 3f – Thickness-Dependent SHG and Nonlinear Engineering: The observed oscillatory behavior due is well-explained. The reviewer's question about leveraging this for "special nonlinear properties" is insightful. The authors should consider adding a brief perspective on whether this predictable thickness-dependent response could be used to engineer structures to enhance or tailor nonlinear efficiency at target wavelengths.
9. SPDC Brightness: The manuscript reports coincidence rates. A discussion on the absolute brightness (pairs/s/mW of pump power) and its implication for current applications is necessary. How does this brightness compare with state-of-the-art miniaturized sources? Acknowledging the current performance level and discussing potential pathways for improvement would provide a balanced and forward-looking view.
10. Supplementary Information:
o Regarding Figure S16 (SPDC Spectrum) and $g^{(2)}(0)$ measurement: The text should clarify if the setup diagram for the $g^{(2)}(0)$ measurement is included in the supplementary information. If not, a brief description or reference should be added.
o Table S1: This is useful. As suggested in point 7, including data from prominent integrated waveguide systems (PPLN, PPKTP) in this table would enhance its comparative value.
Summary:
This work presents a solid and promising contribution to the field of quantum photonic materials. Addressing the above points will significantly enhance its clarity, depth, and appeal to a wide audience in both the materials science and quantum photonics communities.

Reviewer #2

(Remarks to the Author)
File Attached

Version 1:

Reviewer comments:

Reviewer #1

(Remarks to the Author)
The authors have provided a detailed, point-by-point response to the comments raised by Reviewer 1 and Reviewer 2. They have also submitted a revised manuscript with extensive modifications, including new experimental data (extended refractive index measurements up to 1450 nm, absorption spectra via differential transmission method, error bars on key figures), expanded tables (Table S1 with comprehensive brightness and $\chi^{(2)}$ comparisons), corrected terminology, and improved logical flow. The rebuttal letters are thorough and scientifically sound.

After careful evaluation of the authors' responses and the revised manuscript, I conclude that the authors have adequately addressed the major concerns raised by both reviewers. The remaining points are either clarified to satisfaction or represent minor formatting issues that do not compromise the scientific merit. I recommend acceptance of the manuscript in its current form.

Reviewer #2

(Remarks to the Author)
Comments to the authors
Reviewer 2

Regarding comment 1.
The authors have responded to the comments on these highly important research papers in an elaborate, insightful, and scientifically sound manner. (Nature Photonics 19 (2025): 142–148.; Nature Communications 16 (2025): 1899.; Science Advances 11 (2025): adt3710; Small 2024, 21, 2407729; Advanced Materials 36 (2024): 2400858; Advanced Quantum Technologies, e00551, 2025; https://doi.org/10.21203/rs.3.rs-6420732/v1). The reviewer strongly recommends incorporating these important papers into the reference list of the main manuscript, as they further support the scientific context and

enhance the overall quality and relevance of the manuscript.

Regarding comment 2.
Author clarified the comment.

Regarding comment 3 and 4.
The dielectric screening effect should be carefully considered and discussed in greater detail. Furthermore, incorporating theoretical predictions or calculations would significantly strengthen the manuscript and provide deeper insight into the underlying physical behavior.

Regarding comment 5.
The authors have included the absorption spectrum of $NbOI_2$ measured using the differential transmission method, covering a broad spectral range from 300 to 1000 nm. The origin of the absorption peak observed at 350 nm has not been adequately discussed in the manuscript. The authors should clarify the physical origin of this peak and explain how it influences the optical properties, particularly the nonlinear optical response and related photophysical behavior.

Regarding comment 6.
Addressed.

Open Access This Peer Review File is licensed under a Creative Commons Attribution 4.0 International License, which permits use, sharing, adaptation, distribution and reproduction in any medium or format, as long as you give appropriate credit to the original author(s) and the source, provide a link to the Creative Commons license, and indicate if changes were made.
In cases where reviewers are anonymous, credit should be given to 'Anonymous Referee' and the source.
The images or other third party material in this Peer Review File are included in the article's Creative Commons license, unless indicated otherwise in a credit line to the material. If material is not included in the article's Creative Commons license and your intended use is not permitted by statutory regulation or exceeds the permitted use, you will need to obtain permission directly from the copyright holder.
To view a copy of this license, visit https://creativecommons.org/licenses/by/4.0/

REVIEWER COMMENTS

Response to Reviewer 1.

General Comments: This manuscript presents a systematic and valuable investigation into the second-order nonlinear optical properties and spontaneous parametric down-conversion (SPDC) capabilities of two-dimensional (2D) materials $NbOBr_2$ and $NbOI_2$, expanding the established $NbOCl_2$-based quantum source platform. The work is well-structured, experimentally sound, and provides new candidates for integrated quantum photonic sources. The findings, particularly the demonstration of polarization entanglement from $NbOI_2$, are significant for the field. However, several aspects require clarification, expansion, or correction to enhance the manuscript's impact, rigor, and readability for a broad audience.

Response: We thank the reviewer for the positive and insightful comments, especially their recognition of our research on the second-order nonlinear optical properties and spontaneous parametric down conversion (SPDC) performance of the two-dimensional (2D) materials $NbOBr_2$ and $NbOI_2$. We also value the reviewer's acknowledgment that the demonstration of polarization entanglement in $NbOI_2$ makes a significant contribution to integrated quantum photonic sources. We have carefully considered the reviewer's specific questions and provide detailed point-by-point responses below, including all corresponding modifications or supplementary explanations. We believe these revisions will enhance the manuscript's scientific impact and make it more accessible to the audience. The following are our point-by-point responses to the reviewer's specific comments.

Major Comments:

Comment 1. The abstract and introduction effectively position the work as adding new candidates to the SPDC material library. However, a more explicit discussion of the practical benefits and advantages of these $NbOX_2$ materials compared to current rapid or established material systems (e.g., PPLN, PPKTP waveguides, other 2D materials) is needed. While the Supplementary Information Table S1 is a good start, the main text should highlight key comparative metrics such as nonlinear strength per unit thickness, birefringence, operational wavelength range (especially near-infrared), and intrinsic integration advantages. Specifically addressing the question "Why choose $NbOI_2$ over a mature PPLN waveguide for a specific application?" would greatly strengthen the manuscript's impact.

Response: Thank you very much for your insightful comment regarding a more explicit discussion of the practical benefits and advantages of $NbOX_2$ materials. To address the question "Why choose $NbOI_2$ over a mature PPLN waveguide for a specific application?", we have conducted a comparative analysis based on key performance indicators, covering both conventional integrated platforms (PPLN, PPKTP waveguides) and other two-dimensional material systems (r-BN, TMDs). Mature micro-nano quantum sources represented by PPLN and PPKTP waveguides exhibit well-established performance. However, their integration into photonic circuits still presents several challenges. Firstly, the fabrication of precise quasi-phase-matching (QPM) gratings via electric-field poling is complex and costly. Besides, their integration onto arbitrary substrates relies on epitaxial growth or wafer bonding techniques, which require strict lattice matching, severely limiting

heterogeneous integration into silicon photonics and fiber optics. In contrast, van der Waals materials can be directly integrated onto arbitrary substrates without lattice matching requirements or chemical bonding, allowing seamless heterogeneous integration with diverse photonic platforms. Secondly, $NbOX_2$ exhibits a significantly higher nonlinear optical coefficient than LN and KTP, resulting in a stronger inherent nonlinear response. Moreover, the atomically thin structure of $NbOX_2$ enables device architectures unavailable for conventional crystals, such as stacking, twisting, and stretching—emerging degrees of freedom for tailoring optical nonlinear responses (*Nature Communications, 2024, 15, 10461*) and achieving phase-match conditions (*Physical Review Letters, 2023, 131, 233801*).

Compared with other two-dimensional materials such as rhombohedral boron nitride (r-BN) and transition metal dichalcogenides (TMDs, e.g., 3R-MoS_2 and 3R-WS_2), $NbOX_2$ exhibits several advantages, such as a higher nonlinear optical coefficient than r-BN. In addition, unavoidable defects in r-BN and TMDs induce strong photoluminescence (PL) at excitonic resonances, which introduce significant background noise, contaminate photon-pair signals, reduce quantum correlation purity, and raise the noise floor in coincidence measurements—thereby restricting the effective operational range and failing to cover important quantum source bands.

In contrast, $NbOI_2$ exhibits strong nonlinear responses in the technologically critical near-infrared band (900–1400 nm, covering telecommunication wavelengths) and is compatible with existing quantum memory and sensing platforms. More importantly, as an indirect bandgap semiconductor, $NbOX_2$ exhibits negligible PL emission, allowing its SPDC signals to remain highly pure and free from photoluminescence noise and thereby providing an ideal platform for quantum-optical applications that require high signal-to-noise ratios, such as heralded single-photon sources and quantum entanglement distribution. Furthermore, $NbOX_2$ possesses remarkable anisotropy and large birefringence, which not only enables it to act as an ultra-thin waveplate for simultaneously manipulating the quantum polarization states beyond the nonlinear susceptibility matrix (*Nature Communications, doi:10.1038/s41467-026-70788-3 (2026), Optica, 2023, 10, 538–543*), but also allows direct achievement of type-II phase matching by tuning the pump polarization angle and wavelength. Therefore, $NbOI_2$ not only fills the gap in high-performance nonlinear 2D materials in the near-infrared, but also demonstrates unique potential for realizing multifunctional quantum photonic devices in fiber-based quantum networks and silicon-photonic integrated systems.

Following your valuable comment, we have added a more comprehensive analysis to the **Introduction** in the revised manuscript, as follows:

"Furthermore, the intricate fabrication process raises manufacturing costs and hinders large-scale batch fabrication. And their integration onto arbitrary substrates relies on epitaxial growth or wafer bonding techniques, which require strict lattice matching, severely limiting heterogeneous integration with silicon photonics or fiber optics." (Page 3, highlighted in yellow)

"…. More importantly, unlike many 2D materials such as MoS_2, WS_2, and h-BN, the distinctive features of $NbOX_2$ lie in its absence of fluorescence, strong in-plane anisotropy, and exceptionally large in-plane birefringence, which hold the potential to function as an ultrathin waveplate to manipulate quantum polarization states and directly achieve type-II phase matching" (Page 4, highlighted in yellow)

In addition, Table S1 in Supplementary Information has also been revised to provide a comprehensive comparison with established systems (**given in response 10).**

Comment 2. The manuscript would benefit from a more seamless logical link between sections, particularly when transitioning from fundamental property characterization (crystal structure, refractive indices, SHG) to the final SPDC and entanglement results. For instance, the statement about $NbOI_2$'s suitability for near-infrared SPDC and 1310 nm operation should be more directly supported by the refractive index data or nonlinear susceptibility predictions extended to those wavelengths (see point 5 below).

Response: Thank you very much for your valuable suggestion regarding the logical flow between sections of our manuscript. As you correctly noted, the statement regarding $NbOI_2$'s suitability for near-infrared SPDC and 1310 nm operation should be more directly supported by refractive index data. In the revised manuscript, we have extended the measured range of the refractive index dispersion from the original 450–1000 nm to 450–1450 nm, which fully covers the 1310 nm wavelength. **(Fig. 2, given in response 5)**.

Comment 3. Experimental Data Presentation and Rigor:

o Error Analysis: A significant omission is the lack of error bars on experimental data points in many figures (e.g., SHG intensity vs. thickness, power-dependent counts). Including error estimates is crucial for assessing the reliability of the fitted slopes (e.g., 2.09, 1.94 for quadratic dependence) and the significance of the observed trends.

o Units and Notation: Please adhere strictly to scientific notation conventions. Ensure a space between numbers and units (e.g., "1030 nm" not "1030nm"). Standardize abbreviations (e.g., "NLO" should be defined at first use and used consistently, or spelled out uniformly if preferred).

Specific Comments and Questions:

Response: We sincerely thank the reviewer for pointing out these important details regarding units, notation, and abbreviations, and we apologize for the omissions. As suggested, we have added error bars to the insets of **Fig. 3a** and **Fig. 3b**, as well as to the corresponding data points in **Fig. 3f. (See Fig R1)**. Refitting data shows that the key fitting parameters (e.g., the exponents for quadratic dependence) are not significantly different from the previous results, supporting the reliability of the original conclusions.

For units and notation, we have added a space between all numerical values and their corresponding units (e.g., nm, μm, Å) in the text, figures, and tables, in accordance with standard scientific writing conventions. For abbreviation standardization, we have explicitly defined "nonlinear optics (NLO)" at its first mention in the main text (page 3, highlighted in yellow), and all subsequent mentions consistently use the abbreviation "NLO" to maintain uniformity. We have also carefully revised the entire manuscript to ensure consistency and compliance.

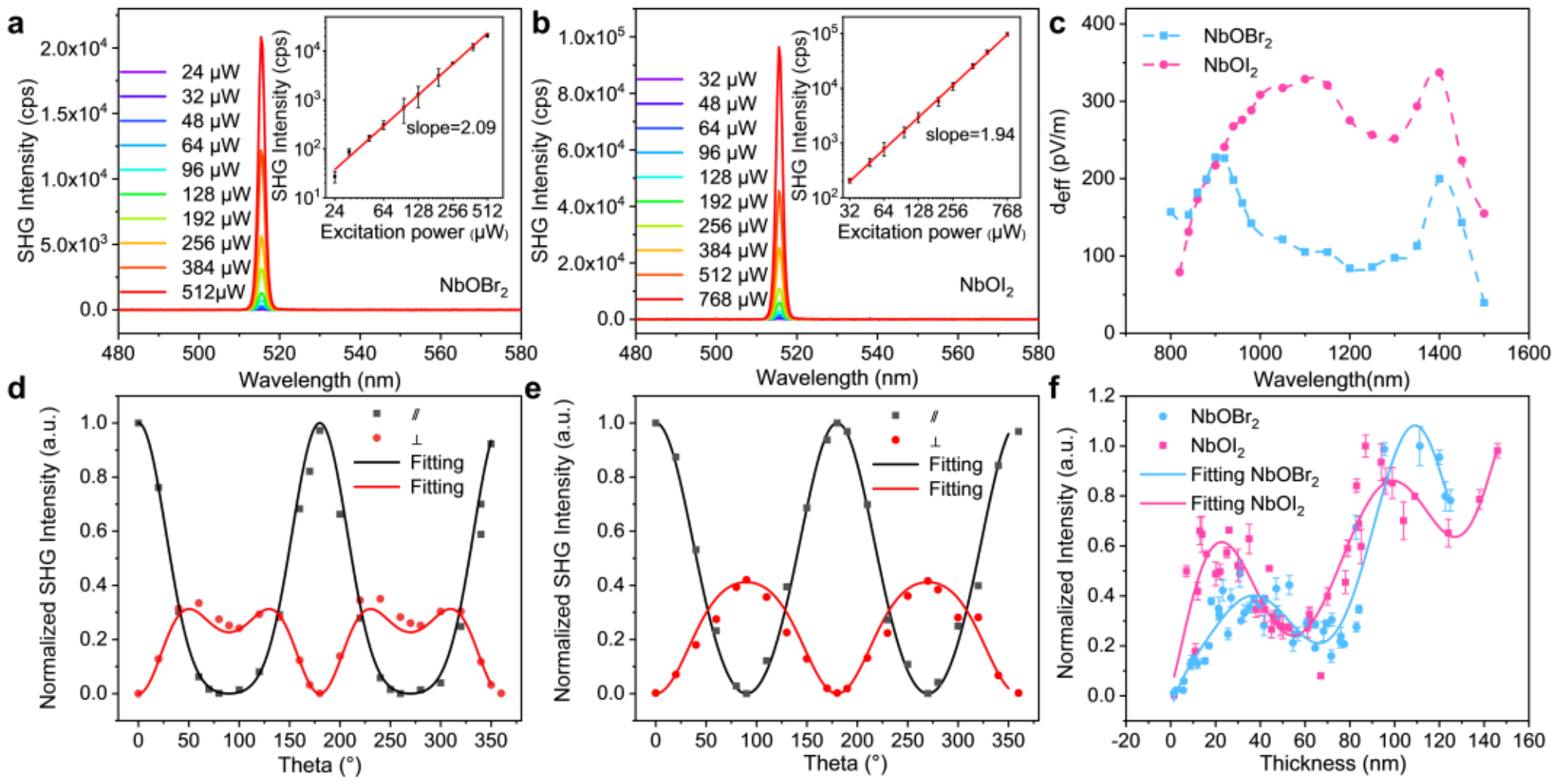

Fig R1 (Fig. 3). Anisotropic and scalable SHG response of the $NbOX_2$. a, b, Power dependent-SHG spectra of $NbOX_2$. Insets, the excitation power dependence of SHG intensity with the slope fitted to be 2.09 for $NbOBr_2$ and 1.94 for $NbOI_2$, indicating the quadratic nature of the nonlinear optical process. (cps: counts per second; Laser spot radius: 1 micrometer). c, Wavelength-dependent effective second-order nonlinear coefficient of $NbOBr_2$(blue) and $NbOI_2$(pink). d, e, Polarization-resolved SHG intensity of $NbOX_2$ along parallel (black) and perpendicular (red) direction. The zero degree is aligned with the crystallographic *b*-axis. f, Thickness-dependent SHG intensity of $NbOBr_2$ and $NbOI_2$ in a broader thickness range under a back-reflection configuration with 1030 nm wavelength pump.

Comment 4. Introduction and Background:

o Line 74: When mentioning on-chip SPDC sources, consider broadening the example beyond PPLN to include other prominent platforms like periodically poled potassium titanyl phosphate (PPKTP) waveguides for a more comprehensive overview.

o Line 92 & Throughout: Standardize the use of "NLO." Define "nonlinear optical (NLO)" at its first occurrence and use the abbreviation thereafter, or avoid it in favor of the full term for consistency.

o 2D Materials Context: For a general audience, a brief, clear definition of 2D materials and their overarching advantages early in the introduction would be very helpful.

Response: We sincerely thank the reviewer for their valuable and constructive suggestions. As suggested, we have added periodically poled potassium titanyl phosphate (PPKTP) as a key nonlinear crystal in the revised manuscript to provide a more comprehensive summary of mainstream integrated SPDC sources, as follows:

"To circumvent these limitations, researchers have developed and demonstrated several new techniques for the on-chip SPDC quantum sources, including ...., and periodically poled potassium titanyl phosphate (PPKTP), ..." (Page 3, highlighted in yellow)

For consistent use of the abbreviation "NLO", we have carefully revised the manuscript. Specifically, we have defined "nonlinear optical (NLO)" at its first occurrence (Page 3, highlighted in yellow) and removed the redundant definition on Page 4. (highlighted in yellow). All subsequent mentions of the term uniformly use the abbreviation "NLO" to ensure clarity and consistency throughout the

text.

In response to the suggestion regarding the context of 2D materials, we have added a brief and clear definition of 2D materials along with their key overarching advantages early in the Introduction, as follows:

"*Materials always have three degrees of freedom in space, however, one degree of freedom in two-dimensional materials is always at atomic thickness scale, which can be negligible and two degrees of freedom are macroscopical or near macroscopical, including 2D layed materials and 2D non-layed materials. Two-dimensional layered materials are one kind of crystalline materials composed of a single atomic layer or a few atomic layers, with strong covalent bonds within the layers and weak van der Waals (vdW) forces between adjacent layers. Their inherent thinness and the compatibility with substrate integration without chemical bonding or lattice matching offer significant integration advantages over conventional nonlinear materials.*" (Page 4, Paragraph 1, highlighted in yellow).

Comment 5. Figure 2 – Refractive Index Dispersion: The experimental data in Figures 2c and 2d are shown only up to ~1050 nm. Given the highlighted potential for operation at 1310 nm (a key telecom wavelength), it is important to extend the experimental measurement or reliable theoretical extrapolation to 1310 nm, or based on the available dispersion model.

Response: We thank the reviewer for their positive feedback on the potential of $NbOI_2$ to generate entangled photons at 1310 nm and the important comment regarding the lack of refractive index data at this wavelength. Accordingly, we have extended the measured range of the refractive index dispersion from the original 450–1000 nm to 450–1450 nm, as shown below, which fully covers 1310 nm **(Fig. 2c** and **2d, See Fig R2)**. In addition, the corresponding theoretical calculations were already presented in **Fig. S11** in the initial Supplementary materials, which may not have been noticed **(See Fig R3)**. These improvements ensure that the refractive index properties at the critical 1310 nm telecommunication wavelength are well supported by both experimental measurements and theoretical calculations.

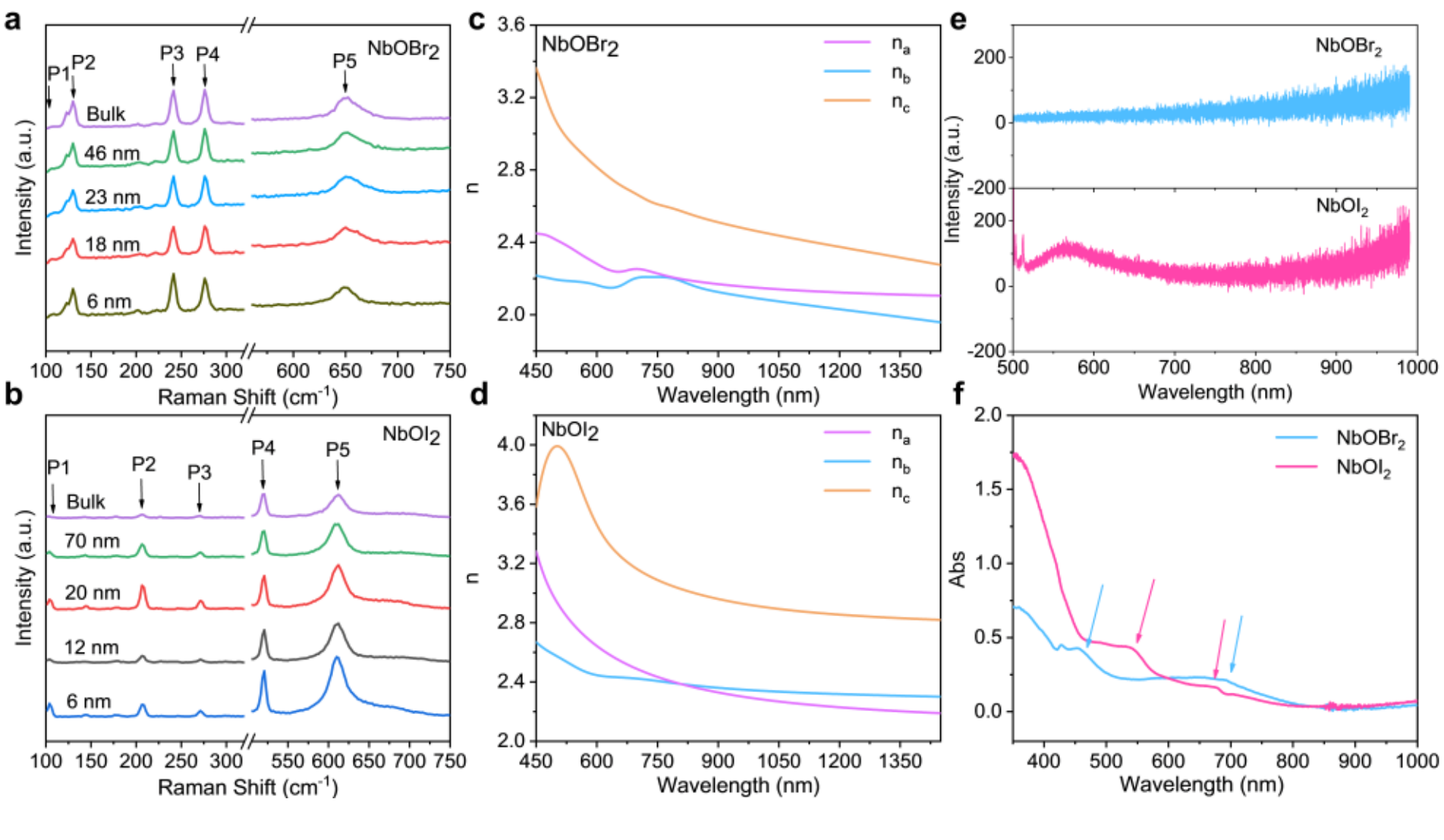

Fig R2 (Fig. 2). Intrinsic optical property characterization. a, b, Raman spectra of 2D $NbOX_2$. c, d, Experimentally measured dispersion curves of refractive indices of $NbOX_2$, where n_a denote

the refractive index perpendicular to the *b*-axis in the (100) plane, n_b and n_c represents the refractive index along *b*- and *c*-axes, respectively. e, PL spectra of $NbOX_2$. f, Experimental absorption spectrum of $NbOX_2$.

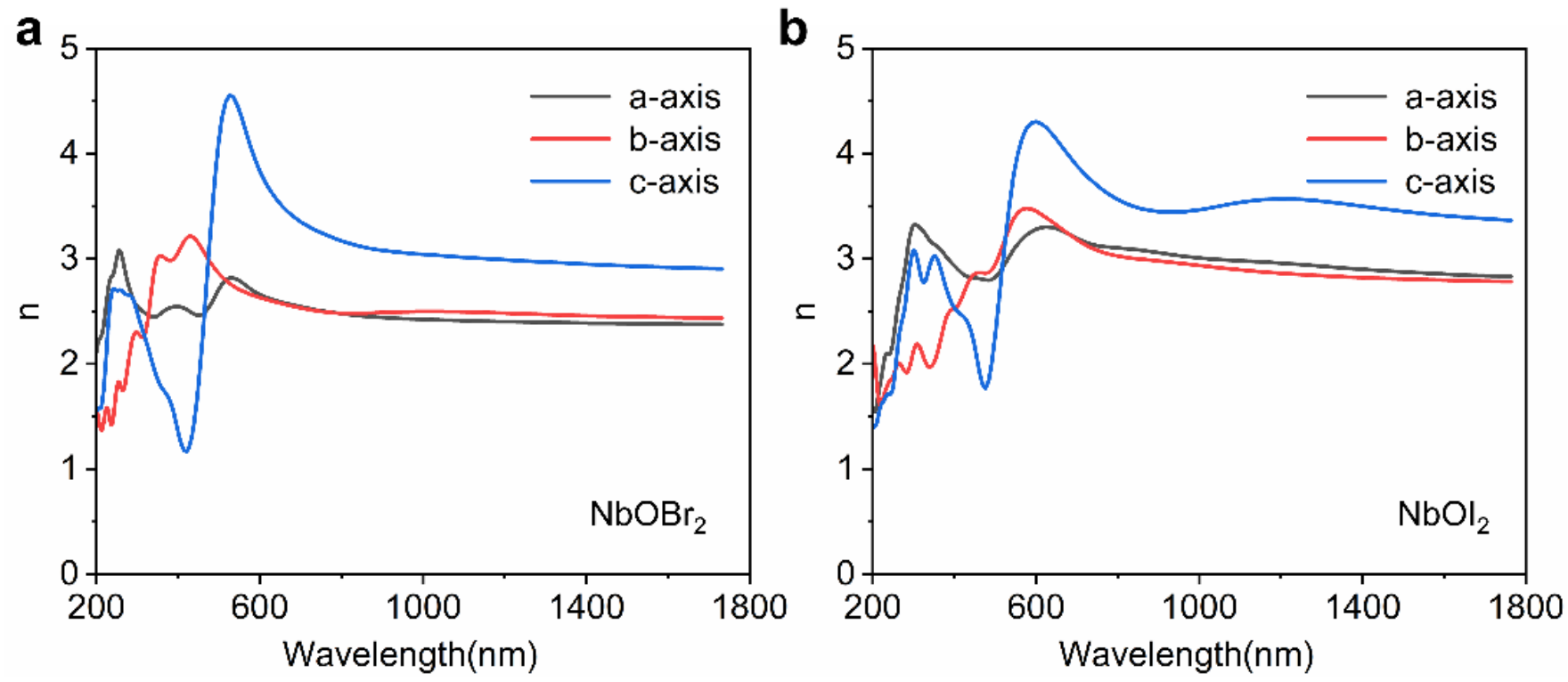

Fig. R3 (Fig. S11). Calculated refractive index of $NbOBr_2$ and $NbOI_2$. The theoretical refractive index n of $NbOBr_2$ **a** and $NbOI_2$ **b** along three crystallographic axes, derived from the GW-BSE calculation.

Comment 6. Figure 2 – Birefringence Comparison: The claim of large birefringence compared to "most conventional birefringent materials" is noted. To strengthen this, a brief quantitative comparison with other thin-film or integrated photonic material platforms would be informative. Clearly state the advantage of such high birefringence for phase-matching in nonlinear processes like SPDC.

Response: We thank the reviewer for this valuable suggestion. Other thin-film materials such as transition metal dichalcogenides (TMDs) are known to exhibit large out-of-plane birefringence (*Nature Communications, 2021, 12, 854*), which is difficult to utilize in our vertical incidence configuration. Lithium niobate (LN) can exhibit in-plane birefringence with x-cut or y-cut orientations, but its birefringence is much weaker. In contrast, the two-dimensional van der Waals crystal $NbOX_2$ exhibits strong in-plane optical anisotropy and birefringence, and its potential advantages in nonlinear SPDC processes have been added in the revised manuscript, as follows: *"This large in-plane birefringence may offer several potential advantages in SPDC. First, it can serve as an ultrathin waveplate to manipulate the polarization state of photon pairs, enabling the design of biphoton polarization states beyond the limits imposed by the nonlinear coefficient matrix and opening a new route for more complex quantum states. Second, its large birefringence may allow direct satisfaction of type-II phase matching by tuning the pump polarization angle and wavelength—without requiring additional structural engineering—thereby significantly enhancing spontaneous parametric down-conversion (SPDC) efficiency. "*（Page 9 and Page 10, highlighted in yellow）

Comment 7. Figure 3 – SHG Analysis and Efficiency:

o The SHG intensity plots require units on the y-axis.

o Following the methodology in Supplementary Section 4, please provide an estimated second-order nonlinear susceptibility ($\chi^{(2)}$) or effective nonlinear coefficient (d_eff) for both materials at the measured wavelengths. A direct comparison of these calculated values with those of common commercial bulk (e.g., BBO, $LiNbO_3$) and integrated (e.g., PPLN, PPKTP) materials, perhaps in an

expanded table, would be extremely valuable for assessing their practical potential.

Response: Thank you very much for your valuable comments regarding Figure 3 and the SHG analysis. We have carefully addressed your suggestions as follows:

Regarding the units for the SHG intensity plots, we have added the unit "cps" (counts per second) on the y-axis of **Fig. 3a** and **3b** to clearly indicate the relative intensity scale.

Regarding the estimation of second-order nonlinear susceptibility $\chi^{(2)}$, we have calculated the $\chi^{(2)}$ values for both $NbOBr_2$ and $NbOI_2$ at the measured wavelengths. Specifically, for $NbOBr_2$, we obtained $\chi^{(2)}$ values at 914 nm, 900 nm, 1310 nm, and 1400 nm; for $NbOI_2$, we obtained corresponding $\chi^{(2)}$ values at 914 nm, 1100 nm, 1310 nm, and 1400 nm. These values are summarized in **Table R1**, where we systematically compare them with several typical conventional materials (e.g., BBO, LN, KTP). As shown in the table, both $NbOBr_2$ and $NbOI_2$ exhibit significantly higher second-order nonlinear susceptibility than these conventional materials.

Following your suggestion to provide a more comprehensive comparison, we have integrated all data from **Table R1** into the expanded Table S1 (given in Response 10) to compare the nonlinear susceptibilities of common commercial bulk materials (e.g., BBO), and integrated nonlinear materials (e.g., PPLN, PPKTP), highlighting the promising potential of $NbOBr_2$ and $NbOI_2$ in nonlinear quantum sources.

Table R1. The d_{eff} of $NbOX_2$ compared with traditional nonlinear materials.

Materials	Wavelength (nm)	$\chi^{(2)}$ [pm/V]	Reference
PPLN	1560	40	*Conference on Lasers and Electro-Optics,2024,1–2*
PPKTP	1569	10.7	*Scientific Reports, 2021, 11(1): 12628*
$LiNbO_3$	1375	40	*Optics Letters 46, 653–656*
BBO	702.2	3.9	*Physical Review A, 2001, 64(2): 023802*
$NbOCl_2$	808	200	*Nature, 2023, 613, 53–59*
$NbOBr_2$	900	227	This work
	914	232	
	1300	98	
	1400	200	
$NbOI_2$	914	234	This work
	1100	329	
	1300	252	
	1400	337	

Comment 8. Figure 3f – Thickness-Dependent SHG and Nonlinear Engineering: The observed oscillatory behavior due is well-explained. The reviewer's question about leveraging this for "special nonlinear properties" is insightful. The authors should consider adding a brief perspective on whether this predictable thickness-dependent response could be used to engineer structures to enhance or tailor nonlinear efficiency at target wavelengths.

Response:

Thank you very much for your insightful comment regarding Figure 3f and the potential for leveraging thickness-dependent SHG behavior in nonlinear engineering. Following your advice, we have added a brief perspective in the revised manuscript, as follows:

"*In addition, given that the coherence length is wavelength-dependent, the thickness corresponding to the maximum SHG intensity also varies with wavelength. By selecting an appropriate thickness, the SHG efficiency can be optimized for specific target wavelengths.*" (page 13, highlighted in yellow).

Comment 9. SPDC Brightness: The manuscript reports coincidence rates. A discussion on the absolute brightness (pairs/s/mW of pump power) and its implication for current applications is necessary. How does this brightness compare with state-of-the-art miniaturized sources? Acknowledging the current performance level and discussing potential pathways for improvement would provide a balanced and forward-looking view.

Response: Thank you very much for your valuable comment. In response to your suggestion, we have provided a comparison of the absolute brightness (pairs/s/mW) in **Table R2**, showing that the brightness of our source is comparable to state-of-the-art miniaturized SPDC sources at the same thickness. These results underscore the great potential of $NbOX_2$ compounds as compact, efficient quantum light sources. As you rightly pointed out, this type of ultra-compact, ultra-thin quantum light source is still in its early research stage, and its absolute brightness remains far from the requirements for practical applications. Looking forward, we anticipate that integrating $NbOBr_2$ and $NbOI_2$ with photonic structures such as metasurfaces and cavities could improve the photon-pair generation efficiency through enhanced light–matter interaction. On the other hand, another promising pathway for performance improvement is to integrate these thin-film materials with waveguide resonators. This can be realized by designing phase-matching conditions through birefringence or periodic modulation of stacking directions. Such advances would accelerate the development of ultra-compact quantum light sources and open new opportunities in quantum computing, quantum sensing, and quantum simulation. We have added these discussions in the revised manuscript as follows:

"*Despite achieving brightness comparable to the brightest 2D materials for SPDC reported to date (Table S1), this ultra-compact and ultra-thin quantum light source is still in the early research stage, and its absolute brightness remains far from practical application levels. To overcome this limitation, future work will focus on combining this van der Waals platform with advanced micro- and nano-optical architectures, such as cavity-photonics, metasurfaces, and integrated optical platforms, we anticipate realizing significant advancements in both scientific research and applications, propelling the development of ultra-compact quantum light sources, which will further advance frontiers in the fields of quantum computing, quantum sensing, and quantum simulation.*" (Page 18, highlighted in yellow)

Table. R2 Absolute brightness comparison of miniaturized SPDC sources

Materials	Wavelength (nm)	Thickness (nm)	Brightness (pairs/s/mW)	Reference
TFLN	1570	304	0.021	*Science advances, 2022, 8(30): eabq4240*
LN film	1375	300	0.31	*Optics Letters 46, 653–656*
GaP film	1375	400	0.022	*Journal of the Optical Society of America B, 1997, 14(9): 2268–2294*
rBN	918	1.47×10^3	8	*Nature Communications, 2025, 16(1): 1899*
	810	910	9.15	*Science Advances, 2025, 11(4): eadt3710*
3R-WS_2	914	350	1.71	*elight, 2024, 4(1): 16*
3R-MoS_2	1576	285	8.0×10^{-3}	*Nature Communications, 2024, 15(1): 7600*
$NbOCl_2$	808	150	1.46	*Nature, 2023, 613(7942): 53–59*
Twisted $NbOCl_2$	818	1.2×10^3	0.57	*Nature Photonics, 2025, 19(2): 142–148*
$NbOBr_2$	914	145	1.5	This work
	900	/	/	
	1300	100	0.04	
	1400	/	/	
$NbOI_2$	914	150	0.9	This work
	1100	/	/	
	1300	100	0.13	
	1400	/	/	

Comment 10. Supplementary Information:

o Regarding Figure S16 (SPDC Spectrum) and $g^{(2)}(0)$ measurement: The text should clarify if the setup diagram for the $g^{(2)}(0)$ measurement is included in the supplementary information. If not, a brief description or reference should be added.

o Table S1: This is useful. As suggested in point 7, including data from prominent integrated waveguide systems (PPLN, PPKTP) in this table would enhance its comparative value.

Response: Thank you for your helpful suggestions regarding the supplementary information. We

have addressed both points as follows:

1. Response to the $g^{(2)}(0)$ measurement setup: We confirm that the schematic diagram of the experimental setup for the second-order correlation measurement is already included in the Supplementary Information (**Fig. S16**), and the setup for SPDC spectrum measurement is the same as Fig. S16 expect for an additional dispersion fiber. A corresponding description has also been added to the figure caption to clarify the measurement configuration, as follows:

"*Fig. S16. ... the setup for SPDC spectrum measurement is the same, but with a multimode fiber of 300 m length added before each single-photon detector.*" (Supplementary materials, Page 15, highlighted in yellow)

2. Response to Table S1: Following your valuable suggestion, we have now expanded Table S1 to include representative data from prominent integrated waveguide systems, such as PPLN and PPKTP. Additionally, to provide a more comprehensive comparison with state-of-the-art two-dimensional SPDC sources, we have also supplemented the table with brightness data from recently reported 2D nonlinear materials for SPDC, including r-BN and twisted $NbOCl_2$. The revised **Table S1** is provided in the Supplementary Information and shown below. We are grateful for your thoughtful feedback.

Table S1 Comparison of different materials for SPDC sources.

Materials	Wavelength (nm)	$\chi^{(2)}$ [pm/V]	Thickness (nm)	Brightness (Hz/mW)	Normalized Brightness (Hz/(mW•nm))	Normalized Brightness (Hz/(mW•nm²))	Reference
PPLN	1560	40	4×10^{7}	3×10^{5}	0.03	7.5×10^{-10}	1
PPKTP	1569	10.7	1×10^{7}	2.1×10^{4}	2.1×10^{-3}	2.1×10^{-10}	2
TFLN	1570	40	304	0.021	7.0×10^{-5}	2.3×10^{-7}	3
BBO	702.2	3.9	2×10^{6}	900	4.5×10^{-4}	2.25×10^{-10}	4
LN film	1375	40	300	0.31	1.02×10^{-3}	3.4×10^{-6}	5
GaP film	1375	37	400	0.022	5.6×10^{-5}	1.4×10^{-7}	5,6
rBN	918	40	1.47×10^{3}	8	5.4×10^{-3}	3.7×10^{-6}	7
	810	40	910	9.15	0.01	1.1×10^{-5}	8
$3R\text{-}WS_2$	914	/	350	1.71	4.9×10^{-3}	1.4×10^{-5}	9
$3R\text{-}MoS_2$	1576	30	285	8.0×10^{-3}	2.8×10^{-5}	9.8×10^{-8}	10
$NbOCl_2$	808	200	150	1.46	9.8×10^{-3}	6.5×10^{-5}	11
Twisted $NbOCl_2$	818	200	1.2×10^{3}	0.57	4.68×10^{-5}	3.9×10^{-7}	12
$NbOBr_2$	914	232	145	1.5	1.03×10^{-2}	7.1×10^{-5}	Our work
	900	227	/	/		/	
	1300	98	100	0.04	4×10^{-4}	4×10^{-6}	
	1400	200	/	/		/	
$NbOI_2$	914	234	150	0.9	6×10^{-3}	4×10^{-5}	Our work
	1100	329	/	/		/	
	1300	252	100	0.13	1.3×10^{-3}	1.3×10^{-5}	
	1400	337	/	/		/	

Reference:

[1] Conference on Lasers and Electro-Optics,2024,1–2;

[2] Scientific Reports, 2021, 11(1): 12628;
[3] Science advances, 2022, 8(30): eabq4240;
[4] Physical Review A, 2001, 64(2): 023802;
[5] Optics Letters 46, 653-656;
[6] Journal of the Optical Society of America B, 1997, 14(9): 2268–2294;
[7] Nature Communications, 2025, 16(1): 1899,
[8] Science Advances, 2025, 11(4): eadt3710;
[9] elight, 2024, 4(1): 16;
[10] Nature Communications, 2024, 15(1): 7600;
[11] Nature, 2023, 613(7942): 53–59;
[12] Nature Photonics, 2025, 19(2): 142–148

Summary:
This work presents a solid and promising contribution to the field of quantum photonic materials. Addressing the above points will significantly enhance its clarity, depth, and appeal to a wide audience in both the materials science and quantum photonics communities.

Response: We sincerely thank you for your positive evaluation of our work. We are also grateful for your thoughtful and constructive suggestions. We believe the revised manuscript has improved the clarity and depth of our work and meets the interests of the broader materials science and quantum photonics communities.

Response to Reviewer 2.

General Comments: In this manuscript, Duan and co-workers investigate the second-order nonlinear optical response of $NbOX_2$ and examine its intrinsic physicochemical properties. Spontaneous parametric down conversion (SPDC) is also studied. The authors report an entangled-state concurrence of 0.90 ± 0.05 and a theoretical fidelity of 0.94 ± 0.05. However, the reported brightness of the $NbOBr_2$ crystal (1.5 Hz/mW) is significantly low compared to recent reports on the same and other van der Waals materials. Consequently, the claim that $NbOBr_2$ represents a novel nonlinear quantum light source is not justified, as several studies have already reported s results using this material as well as other van der Waals systems [Nature Photonics 19 (2025): 142–148.; Nature Communications 16 (2025): 1899.; Science Advances 11 (2025): adt3710; Small 2024, 21, 2407729 ; Advanced Materials 36 (2024): 2400858; Advanced Quantum Technologies, e00551, 2025; Nanophotonics, vol. 14, no. 27, 2025, pp. 5337–5345; Nature Communications 14 (2023): 5911; " Nature 613 (2023): 53–59; Applied Physics Letters 125 (2024): 151902]. The quantum characterization presented in this work is insufficient. Quantum state tomography is a critical requirement for establishing an entangled quantum light source; however, the manuscript lacks rigorous, detailed, and convincing experimental evidence. The absence of novelty, comprehensive and statistically significant results makes not to support publication in Nature Communications, particularly given the journal's broad and multidisciplinary scientific audience.

The paper is scientifically weak in terms of novelty and broader impact, and it does not meet the standards required for publication in Nature Communications.

Response: We sincerely thank the reviewer for the careful reading, but it seems that the reviewer has some misunderstanding of our work. The reviewer's concern about the relatively low brightness of our material arises from the different calculation methods adopted to characterize brightness in our work and in the literature listed by reviewer. We demonstrate brightness comparable to all these prior works when using the same calculation method, as explained in detail in the following responses. We have carefully considered the reviewer's specific comments and provided a detailed point-by-point response below, including corresponding revisions and additional clarifications. We believe that the revised manuscript now comprehensively and convincingly demonstrates that $NbOBr_2$ and $NbOI_2$ are highly promising nonlinear quantum light sources. We sincerely hope that the revised version addresses the reviewer's concerns and merits reconsideration for publication in *Nature Communications*.

Specific Comments:

Comment 1.The authors report an entangled-state concurrence of 0.90 and a theoretical fidelity of 0.94 ± 0.05. However, the reported brightness of the $NbOBr_2$ crystal (1.5 Hz/mW) is significantly low compared to recent reports using the same material as well as other van der Waals (vdW) materials (for eg. Nature Photonics 19 (2025): 142–148.: $NbOCl_2$, 31KHz/mW.mm" Nature Communications 16 (2025): 1899: r-BN, 8667 Hz/mW.mm,). Despite this, the authors claim the system to be a novel nonlinear quantum light source, which is not accurate. There is no comparisons of results and scientific reasons of lower brightness. Several studies have already reported similar & extremely high brigntness or generation rate with Fidelity and CAR results using $NbOBr_2$ and

other vdW materials, undermining the claimed novelty. [Nature Photonics 19 (2025): 142–148.; Nature Communications 16 (2025): 1899.; Science Advances 11 (2025): adt3710; Small 2024, 21, 2407729 ; Advanced Materials 36 (2024): 2400858; Advanced Quantum Technologies, e00551, 2025; https://doi.org/10.21203/rs.3.rs-6420732/v1]

Response: Thank you for raising this important point regarding the reported brightness. As provided by the reviewer, the core discrepancy lies in the different units adopted in our work (Hz/mW) and other studies (Hz/(mW·mm)). We appreciate the opportunity to clarify the potential misunderstanding arising from inconsistent brightness calculation standards across different works. The following detailed comparisons with the references suggested by the reviewer are provided.

1. Nature Photonics 19 (2025): 142–148 ($NbOCl_2$)

This work reports polarization entangled photon pairs generated from a vertically stacked $NbOCl_2$ structure with thicknesses of 960 nm and 280 nm. The reported value of 31 kHz mW^{-1} mm^{-1} in this paper is the estimated photon pair generation rate after compensating for detection loss, not the directly measured brightness. The actual experimentally measured count rate after normalization to pump power and interaction length is 472 Hz mW^{-1} mm^{-1}. For a direct comparison, if we apply the same methodology to estimate the photon-pair generation rate of our $NbOBr_2$, the measured coincidence count rate normalized to pump power and interaction length is 10300 Hz mW^{-1} mm^{-1}. When corrected for detection loss, the estimated photon-pair generation rate reaches 39.6 kHz $mW^{-1}mm^{-1}$. Both values are higher than the corresponding figures reported for $NbOCl_2$.

2. Nature Communications 16 (2025): 1899 (r-BN)

This work demonstrates entangled photon pair generation in r-BN with a thickness of 1.47 μm. The reported value of 8667 Hz mW^{-1} mm^{-1} is normalized to pump power and interaction length. Using the same method, our calculated result is 10300 Hz $mW^{-1}mm^{-1}$, which is superior to that of r-BN.

3. Science Advances 11 (2025): adt3710 (r-BN)

This work reports a polarization entangled photon source based on r-BN with a thickness of 910 nm. The calculated thickness-normalized brightness is 0.01 $mW^{-1}mm^{-1}$, and the intrinsic brightness of the material is 1.1×10^{-5} Hz/(mW·nm^2), which is also comparable to that of $NbOBr_2$ in our work.

4. Small 2024, 21, 2407729: This work focuses on the synthesis, crystal structure, optical properties, and in-plane ferroelectric characteristics of $NbOBr_2$. It mainly characterizes the basic material properties of $NbOBr_2$, but does not involve spontaneous parametric down-conversion (SPDC), entangled photon pair generation, or quantum light source applications.

5. Advanced Materials 36 (2024): 2400858: This study investigates second-harmonic generation (SHG) and third-harmonic generation (THG) in patterned hole structures of $NbOBr_2$. It emphasizes nonlinear optical characterization at the classical optics level, with no SPDC process, entangled photon state preparation, and quantum source performance.

6. Advanced Quantum Technologies 2025, e00551: This is a comprehensive review article that summarizes recent progress in chip-integrated quantum photonic devices based on two-dimensional materials. It provides an overview of material platforms, device architectures, and potential applications, yet contains no original experimental data or results regarding SPDC and entangled photon generation in $NbOBr_2$ or $NbOI_2$.

7. https://doi.org/10.21203/rs.3.rs-6420732/v1: This preprint reports electrically tunable nonlinear optical responses in $NbOBr_2$-integrated photonic circuits, highlighting that strong ferroelectricity enables in-situ modulation of SHG intensity. However, this work is restricted to classical nonlinear optics and does not report SPDC, entangled photon pair generation, concurrence, fidelity, or any

results related to quantum entanglement.

Using a consistent methodology, we further compared our results with other representative SPDC materials, including 3R-WS_2, 3R-MoS_2, PPLN, and PPKTP. Considering the SPDC brightness is proportional to the long interaction length when satisfying the phase-matching condition (e.g., PPLN and PPKTP), yet it becomes nearly proportional to the square of the short interaction length when the interaction length is much less than the coherence length (e.g., 2D materials with sub-wavelength thickness), we used two different normalization methods (Hz/(mW•nm) and Hz/(mW•nm^2)) for comparison in **Table S1** given below. Our $NbOBr_2$ exhibits advantages in intrinsic brightness among the state-of-the-art compact SPDC material library. Meanwhile, $NbOBr_2$ and $NbOI_2$ possess the highest second-order nonlinear coefficients, which represent one of the key novelties of this work; the corresponding comparisons have been updated in the revised **Table S1**.

In summary, our work demonstrates for the first time the great potential of $NbOBr_2$ and $NbOI_2$ as high-performance SPDC quantum light sources. The brightness discrepancy noted by the reviewer mainly arises from different calculation definitions and normalization methods adopted in different reports. After fair comparison under unified metrics, our $NbOBr_2$ shows competitive or even superior intrinsic brightness. Notably, $NbOI_2$ can directly generate polarization-entangled states with a measured concurrence of 0.90±0.05 and a fidelity of 0.94±0.03.

Through systematic characterization of the crystal structures and intrinsic properties, we clarify the modulation effects of lattice distortion and material absorption on the effective second-order nonlinear optical coefficients. We also reveal that the emission intensity of the nonlinear source is strongly influenced by refractive index dispersion, a previously unreported perspective. These findings fully confirm the distinct novelty and scientific merit of this work on new nonlinear quantum light source.

Table S1 Comparison of different materials for SPDC sources.

Materials	Wavelength (nm)	$\chi^{(2)}$ [pm/V]	Thickness (nm)	Brightness (Hz/mW)	Normalized Brightness (Hz/(mW•nm))	Normalized Brightness (Hz/(mW•nm^2))	Reference
PPLN	1560	40	4×10^{7}	3×10^{5}	0.03	7.5×10^{-10}	1
PPKTP	1569	10.7	1×10^{7}	2.1×10^{4}	2.1×10^{-3}	2.1×10^{-10}	2
TFLN	1570	40	304	0.021	7.0×10^{-5}	2.3×10^{-7}	3
BBO	702.2	3.9	2×10^{6}	900	4.5×10^{-4}	2.25×10^{-10}	4
LN film	1375	40	300	0.31	1.02×10^{-3}	3.4×10^{-6}	5
GaP film	1375	37	400	0.022	5.6×10^{-5}	1.4×10^{-7}	5,6
rBN	918	40	1.47×10^{3}	8	5.4×10^{-3}	3.7×10^{-6}	7
	810	40	910	9.15	0.01	1.1×10^{-5}	8
3R-WS_2	914	/	350	1.71	4.9×10^{-3}	1.4×10^{-5}	9
3R-MoS_2	1576	30	285	8.0×10^{-3}	2.8×10^{-5}	9.8×10^{-8}	10
$NbOCl_2$	808	200	150	1.46	9.8×10^{-3}	6.5×10^{-5}	11
Twisted $NbOCl_2$	818	200	1.2×10^{3}	0.57	4.68×10^{-5}	3.9×10^{-7}	12
$NbOBr_2$	914	232	145	1.5	1.03×10^{-2}	7.1×10^{-5}	Our work
	900	227	/	/		/	
	1300	98	100	0.04	4×10^{-4}	4×10^{-6}	

	1400	200	/	/		/	
$NbOI_2$	914	234	150	0.9	6×10^{-3}	4×10^{-5}	Our work
	1100	329	/	/		/	
	1300	252	100	0.13	1.3×10^{-3}	1.3×10^{-5}	
	1400	337	/	/		/	

Reference:
[1] Conference on Lasers and Electro-Optics,2024,1–2;
[2] Scientific Reports, 2021, 11(1): 12628;
[3] Science advances, 2022, 8(30): eabq4240;
[4] Physical Review A, 2001, 64(2): 023802;
[5] Optics Letters 46, 653–656;
[6] Journal of the Optical Society of America B, 1997, 14(9): 2268–2294;
[7] Nature Communications, 2025, 16(1): 1899,
[8] Science Advances, 2025, 11(4): eadt3710;
[9] elight, 2024, 4(1): 16;
[10] Nature Communications, 2024, 15(1): 7600;
[11] Nature, 2023, 613(7942): 53–59;
[12] Nature Photonics, 2025, 19(2): 142–148

Comment 2. This is an incorrect statement!!! 'As the first reported 2D material-based nonlinear quantum source, ferroelectric van der Waals materials NbOCl2, with distinctive distorted structure exhibits several notable properties' The first report was published on 2D transition metal dichalcogenides.

Response: We appreciate the reviewer for checking the relevant literature. We have re-examined the related publications carefully to confirm our claim and we would like to clarify this statement in the following.

We guess that the reviewer may refer to the pioneering work on 2D transition metal dichalcogenides (3R-WS_2) published in eLight (*eLight, 2024, 4, 16*), which demonstrated the first polarization-entangled photon pairs from a 2D material via the SPDC process, or the first observation of single-photon emitters (SPEs) in semiconductor WSe_2 reported in Nature Nanotechnology (*Nature nanotechnology, 2015, 10(6): 491–496; Nature nanotechnology, 2015, 10(6): 497–502; Nature nanotechnology, 2015, 10(6): 503–506; Nature nanotechnology, 2015, 10(6): 507–511.*)

We would like to clarify the distinctions among the work on $NbOCl_2$, 3R-WS_2, and WSe_2 single-photon emitters.

First, we clarify the distinction between the work on $NbOCl_2$ and 3R-WS_2:

Our manuscript states that $NbOCl_2$ represents the first 2D material-based nonlinear quantum source via second-order nonlinear optics *(Nature, 2023, 613, 53–59)*. Owing to its low crystal symmetry, $NbOCl_2$ exhibits a complex nonlinear susceptibility tensor, which leads to complicated optical fields after nonlinear conversion and poorly defined entanglement in the generated photon pairs. Therefore, pristine $NbOCl_2$ itself does not support the generation of well-defined entangled polarization states, even though it clearly generates quantum photon pairs (entangled in time and frequency). Notably, subsequent studies have successfully realized quantum entanglement based on the SPDC process by orthogonally stacking $NbOCl_2$ layers (*Nat. Commun.,2024, 15, 10461; Nat. Photonics., 2025,*

19, 142–148).

The later work on 3R-WS_2 (*eLight, 2024, 4, 16*) represents the first 2D material in which well-defined polarization-entangled photon pairs were successfully generated via the SPDC process.

Both works rely on the quantum second-order nonlinear optical process (SPDC), but they correspond to different milestones in 2D nonlinear quantum photonics:

$NbOCl_2$: the first 2D material showing SPDC emission (the first 2D material-based nonlinear quantum source).

3R-WS_2: the first 2D material achieving well-defined polarization-entangled photon pairs via SPDC.

Second, we clarify the fundamental difference between nonlinear quantum source ($NbOCl_2$) and the WSe_2 single-photon emitters reported in the 2015 Nature Nanotechnology papers (*Nature Nanotechnology, 2015, 10(6): 491–496; Nature Nanotechnology, 2015, 10(6): 497–502; Nature Nanotechnology, 2015, 10(6): 503–506; Nature Nanotechnology, 2015, 10(6): 507–511*). These works reported the first observation of single-photon emitters in 2D materials and belong to single-photon quantum sources; their emission mechanism essentially originates from fluorescence radiation rather than optical nonlinearity. Importantly, our original content is "*the first reported 2D material-based **nonlinear** quantum source*". $NbOCl_2$ is the first 2D material-based quantum source via second-order nonlinear optical effects (the SPDC process), which generates entangled two-photon quantum states rather than single photons. The two types of sources differ fundamentally in their physical mechanisms, emission characteristics, and research categories, and thus cannot be confused with each other.

Thus, our statement that $NbOCl_2$ is the first reported 2D material-based nonlinear quantum source is correct and consistent with the literature.

If our understanding of the reference mentioned by the reviewer is inaccurate, we would highly appreciate it if the reviewer could kindly specify the related work so that we can revise it accordingly.

Comment 3. Unlike two-dimensional (2D) materials, the Raman peak positions do not exhibit any noticeable shift; however, the intensities of the two modes (P3 and P4) vary irregularly. The observation is unclear.

Response: We sincerely appreciate the reviewer's valuable comment regarding the thickness-dependent Raman behavior of $NbOX_2$. As highlighted in the manuscript, the invariance of all Raman peak positions with increasing thickness unambiguously confirms the weak interlayer coupling in $NbOX_2$, which is distinct from typical 2D materials such as TMDCs. The observed irregular intensity variations of modes P3 and P4 can be well understood as a consequence of thin-film optical interference combined with the strong in-plane anisotropy of $NbOX_2$. Different Raman modes correspond to distinct lattice vibrations with specific polarization responses, and their scattering signals are selectively enhanced or suppressed by thickness-dependent interference at the air/material and material/substrate interfaces. This is an extrinsic optical effect, not an indicator of structural distortion, symmetry modification, or altered interlayer coupling. (*Journal of Physical Chemistry C, 2025, 129, 18092–18111, Journal of materials chemistry C, 2024, 12, 11402–11411).*

To clarify this point, we have added a sentence in the revised manuscript as follows:

"The irregular intensity evolution of Raman peaks with thickness arises from thin-film optical interference and the anisotropic vibrational characteristics of different modes, rather than structural or symmetry changes." (Page 9, Paragraph 1, highlighted in yellow).

Comment 4. The Raman peak positions are signatures of the crystal vibrational modes and are critical for defining the existence and preservation of symmetry. However, the peak energies in the spectra have been overlooked.

Response: We sincerely apologize for the overlooking in the original manuscript. We have carefully revised the relevant sections to address this point.

Specifically, in the figure captions of the Supporting Information (Figures S7 and S8, the polarization-dependent Raman measurements for $NbOBr_2$ and $NbOI_2$), we have now added the specific wavenumbers (in cm^{-1}) for each observed peak, as follows:

"Fig. S7 ....*The polarization-dependent Raman peak intensity of* ***b****, P1 (104.6 cm^{-1});* ***c****, P2 (130.3 cm^{-1});* ***d****, P3 (241.6 cm^{-1});* ***e****, P4 (276.2 cm^{-1}); and* ***f****, P5 (649.7 cm^{-1}) at various polarization angles under parallel (red line) and perpendicular (black line) configuration.*"

"Fig. S8....*The polarization-dependent Raman peak intensity of* ***b****, P1 (104.1 cm^{-1});* ***c****, P2 (206.3 cm^{-1});* ***d****, P3 (271.1 cm^{-1});* ***e****, P4 (519.3 cm^{-1}); and* ***f****, P5 (611.2 cm^{-1}) at various polarization angles under parallel (red line) and perpendicular (black line) configuration.*"

Furthermore, to correlate these positions to the vibrational modes and symmetry, we have added the following physical explanation in the revised manuscript (Page 8, highlighted in yellow)s:

"*The lower-energy peaks (P1–P3) are primarily governed by vibrations involving the Nb–X bonds and collective motions of the octahedral framework, while the higher-energy peaks (P4–P5) arise from vibrations of the stronger Nb–O bonds.*" (*Applied Physics Letters*, *2025*, 126, 062103).

Comment 5. The authors claim that $NbOI_2$ exhibits two main peaks at approximately 550 nm and 700 nm. However, the reviewer believes that the quality of the absorption spectra is extremely poor. If differential reflection measurements cannot be introduced, the resulting conclusions remain ambiguous. Moreover, both crystals exhibit a strong and well-defined peak around 350 nm, which appears to have been intentionally skipped or overlooked.

Response: We sincerely appreciate the reviewer's constructive comment. Following the reviewer's suggestion, we remeasured the absorption spectra using the differential transmission method, considering that our samples were prepared on transparent quartz exfoliation substrates and all optical nonlinear measurements were performed by the transmission method. It is worth noting that the differential transmission method requires the sample thickness to be less than 1/50 of the wavelength (usually within 10 nm) to ensure that the obtained spectrum is proportional to the absorption. Due to the ultra-thin thickness of the sample, the absorption signal is relatively weak, resulting in the low intensity of the absorption peaks at approximately 550 nm and 700 nm. In the previous manuscript, the absorption spectra were measured via the transmission method after subtracting the substrate background, and the test results also show two absorption peaks of $NbOI_2$ at approximately 550 nm and 700 nm. The consistent detection of these two peaks via two different instruments and measurement methods confirms the real existence of the two absorption peaks and their actual influence on the SHG properties of $NbOI_2$. The absorption spectra measured by the differential transmission method have been added to the revised supplementary materials (Page 18, **Fig. S19, See Fig R4**).

With respect to the strong, well-defined peak around 350 nm mentioned by the reviewer, we sincerely apologize for this oversight. Due to limitations in our experimental conditions, we do not have access to single-photon detectors and lasers operating at 350 nm (UV band), which prevented

us from measuring the SHG or SPDC photons at this wavelength. But we agree that the UV absorption peak of the two materials may show potential for UV laser and visible quantum sources, and we would like to address this point in future work with improved experimental setups.

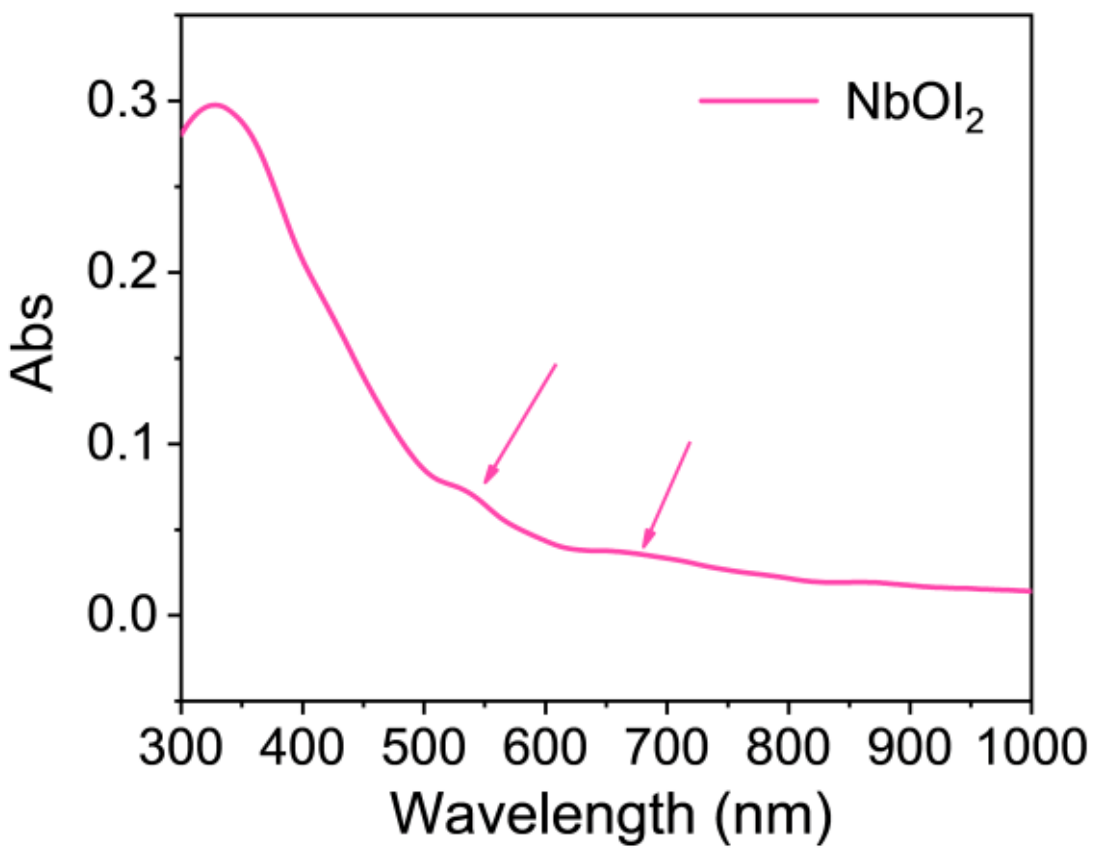

Fig R4 (Fig. S19). Absorption spectrum of $NbOI_2$ measured by differential transmission method. Two weak absorption peaks of $NbOI_2$ at approximately 550 nm and 700 nm were observed.

Comment 6. Line#321/322 ‘is negligible for both materials (see more details in 322 Supplementary Section 4). This appears to be merely superficial, as no specific details are provided to address this comment.

Response: We apologize for the inadvertent error in citing the supplementary section—the relevant details are actually provided in Supplementary Section S5, not S4 as previously stated. We have now corrected this citation in the revised manuscript (Page 13, highlighted in yellow).

The statement that “the contribution is negligible for both materials” is supported by specific calculations presented in Supplementary Section S5, in which we analyze the relative strength of the three effective tensor components in the second-order nonlinear susceptibility matrix of $NbOX_2$ crystals. The key results are as follows:

$NbOBr_2$: $\chi^{(2)}_{bbb} \approx 210, \chi^{(2)}_{bcc} \approx 100, \chi^{(2)}_{cbc} \approx 9.5$

(corresponding to normalized relative strength, not the real strength).

Thus $\chi^{(2)}_{bbb}/\chi^{(2)}_{bcc} \approx 2.10,\ \chi^{(2)}_{cbc}/\chi^{(2)}_{bcc} \approx 0.10$

$NbOI_2$: $\chi^{(2)}_{bbb} \approx 339.94, \chi^{(2)}_{bcc} \approx 218.20, \chi^{(2)}_{cbc} \approx 28.46$

(corresponding to normalized relative strength, not the real strength).

Thus $\chi^{(2)}_{bbb}/\chi^{(2)}_{bcc} \approx 1.56,\ \chi^{(2)}_{cbc}/\chi^{(2)}_{bcc} \approx 0.13$

The calculation details can be found in the revised Supplementary Section S5. The intensity ratio of the polarization states corresponding to the elements follows $I_{cbc}/I_{bcc} = (\chi^{(2)}_{cbc}/\chi^{(2)}_{bcc})^2$, thus we claimed that the contribution from $\chi^{(2)}_{cbc}(\chi^{(2)}_{ccb})$ is negligible for both materials.

Comment 7. The author uses the term “coherent length”, whereas the scientifically accepted term used by the research community is “coherence length.”

Response: We thank the reviewer for the valuable comment on terminology. The non-standard term

"coherent length" has been replaced with the correct scientific term "coherence length" throughout the revised manuscript. (Page 13, highlighted in yellow). In addition, we have corrected this term in the Supplementary Material as well. (Page 14, Supplementary Section S6, highlighted in yellow).

Comment 8. Why did the authors not choose a $NbOBr_2$ thickness corresponding to a shorter coherence length? Initially, in the Introduction, this was presented as an advantage of two-dimensional materials. However, the interpretation of the SHG and SPDC results appears to be contradictory.

Response: Thank you very much for your thoughtful question. First, we would like to clarify that in the Introduction, we did not present a shorter coherence length as an inherent advantage of two-dimensional materials. Instead, we noted that when the material thickness is smaller than the coherence length, the SPDC process exhibits distinct characteristics compared with conventional phase-matched bulk crystals, the original content is as follows:
"*Besides, thin-film based SPDC source exhibits different properties from phase-matched light sources due to the extremely small interaction length resulting from the reduction in size. The relaxation of phase-matching conditions broadens the frequency spectrum for entangled photon pairs, offering potential applications in quantum precision measurement.*" (Page 3 and 4). As we have calculated in Supplementary Section 6, the coherence length of $NbOBr_2$ is actually longer than that of $NbOI_2$ under our experimental conditions. We selected similar thickness of $NbOBr_2$ and $NbOI_2$ to present an intuitive comparison between the two materials.
The reviewer is correct in noting the contradiction between the relative strenghs of SHG and SPDC in the two materials. We would like to emphasize that this observation constitutes one of the key findings of our work. Previous studies have generally assumed that the relative strengths of SHG and SPDC are always positively correlated and have even directly used SHG intensity to estimate SPDC intensity, as both processes originate from the same second-order nonlinear susceptibility $\chi^{(2)}$. However, our results demonstrate that this is not necessarily the case: although $NbOBr_2$ exhibits slightly lower SHG intensity than $NbOI_2$ (914nm to 457nm), its coincidence rate (SPDC intensity) remains superior (457nm to 914nm). Therefore, we have explained this in detail (Page 15, Paragraph 2) and demonstrate the influence of refractive index dispersion on the brightness of nonlinear optical sources in practical experimental measurements. This counterintuitive phenomenon, observed for the first time and revealed by the correlation between the intrinsic refractive index properties of materials and their optical nonlinear responses, constitutes one of the key innovations of this work.

Comment 9. 'the calculated lower efficiencies of NbOI2 comparing to NbOBr2 confirm the aforementioned influence of longitudinal phase mismatch and index contrast.' The statement is overly general and merely reiterates advantages of two-dimensional materials over conventional bulk crystals by citing previous studies. No new fundamental insights or original scientific contributions are presented here.

Response: We apologize for any confusion. We wish to clarify that the term "aforementioned" in our statement refers specifically to our own earlier discussion in the manuscript, as follows:

"*Firstly, the coherence length of the main contribution element* $\chi^{(2)}_{bbb}$ *for* $NbOBr_2$ *(~2450 nm) is significantly longer than the counterpart for* $NbOI_2$ *(~775 nm)(see Supplementary Section 6). This*

implies the degree of phase mismatch for $NbOI_2$ is greater than that for $NbOBr_2$ under this specific thickness and wavelength of the experiment, resulting in a faster decreased normalized relative efficiency. Secondly, the efficiency of second order nonlinear optical conversion inversely proportional to the refractive index of the participating photons: $\eta \propto \frac{1}{n_i \cdot n_s \cdot n_p}$*, while the refractive index contrast at wavelength of pump light and SPDC photon pairs both exhibit $n(NbOI_2)>n(NbOBr_2)$(Fig. 2c and 2d).*" (Page 15, Paragraph 2).

Here, the first point corresponds to the "longitudinal phase mismatch", and the second to the "index contrast" mentioned by reviewer. This statement, along with the sentences presented above, explains the experimental results: why $NbOBr_2$ demonstrates a slightly lower measured SHG intensity yet a clearly higher measured coincidence rate (SPDC intensity) than $NbOI_2$. The entire paragraph is completely unrelated to advantages of two-dimensional materials over conventional bulk crystals. This interesting observation, which has not been documented in previous studies, exactly offers new insights and original scientific contributions to the decoupling of SHG and SPDC efficiencies.

Comment 10. The term used in incorrect 'coincidence-accident-rate' is basically 'coincidence to accidental ratio'.

Response: Thank you for your meticulous review and for pointing out the incorrect terminology. We apologize for misusing the term 'coincidence-accident-rate', and we have replaced it with the scientifically the accurate term 'coincidence to accidental ratio (CAR)' in the revised manuscript. (Page 16, highlighted in yellow).

Comment 11. Author reported that 'NbOBr2 crystal achieves a brightness of 1.5 Hz/mw at 914nm, which is comparable to the performance of NbOCl2 crystal at 808nm', which is pretty low compared to the performance of other reported results same NbOCl2 and other 2D. [Nature Photonics 19 (2025): 142–148.; Nature Communications 16 (2025): 1899.; Science Advances 11 (2025): adt3710.]

Response: We sincerely appreciate the reviewer's great attention to the brightness of our quantum light source, which is indeed a key metric for evaluating SPDC quantum sources. As we have provided a detailed comparison and clarification in our response to the first comment, we briefly restate the core points here for clarity**.**

The seemingly low brightness of our $NbOBr_2$ crystal (1.5 Hz/mW at 914 nm) compared with the referenced works [Nature Photonics 19 (2025): 142–148; Nature Communications 16 (2025): 1899; Science Advances 11 (2025): adt3710] arises from different brightness calculation and normalization standards. Specifically, the high values reported in these references are either photon pair generation rates compensated for detection loss (e.g., the value of 31 kHz mW^{-1} mm^{-1} for $NbOCl_2$ in Nature Photonics 19 (2025) is obtained by dividing the actual measured brightness by the estimated detection efficiency, while the actual measured count rate is 472 Hz mW^{-1} mm^{-1}) or normalized by interaction length and pump power (e.g., the value of 8667 Hz mW^{-1} mm^{-1} for r-BN in Nature Communications 16 (2025)). When compared under the same metric (intrinsic brightness), our $NbOBr_2$ exhibits a directly measured brightness of 10300 Hz mW^{-1} mm^{-1} without being normalized by the detection loss. We again express our gratitude for the reviewer's attention to this key issue; detailed comparisons with the three references, including material thickness and

brightness calculation details, are provided in our response to the first question.

Minor comments,

Comment 1. The manuscript contains numerous typographical errors and requires substantial attention. Overall, it appears to have been written carelessly.

Response: We sincerely apologize for the typographical errors in our manuscript. In response to your comment, we have conducted a thorough, line-by-line revision of the entire manuscript. This includes adding missing spaces, italicizing mathematical symbols, and other minor formatting improvements. All corrections have been highlighted in yellow. We hope that the revised manuscript now meets the expected standards of clarity and professionalism.

Comment 2. 'photonical structures' is incorrect scientific term.

Response: Thank you for your careful reading and for pointing out the incorrect terminology. We apologize for misusing the term 'photonical structures' and have replaced it with the scientifically accurate term 'photonic structures' in the revised manuscript (Page 3, highlighted in yellow).

Comment 3. 'AlN microring', 'LN microdisk' abbreviations without proper scientific pronounced name.

Response: Thank you for your careful reading and for pointing out the missing scientific names for the abbreviations. We apologize for this oversight. In the revised manuscript, we have provided the complete scientific names when these abbreviations first appear: 'AlN microring' has been revised to 'aluminum nitride (AlN) microring', and 'LN microdisk' has been revised to 'lithium niobate (LN) microdisk'. (Page 3, highlighted in yellow).

Comment 4. Inconsistence of the Figure abbreviation in the text. For Eg Line# 284 Figure 3a and 3b and line#247 (Fig. 2f).

Response: We thank the reviewer for the careful reading and for noting the inconsistent abbreviation of "Figure" in the text. We would like to clarify that this usage is intentional and follows the standard formatting guidelines of the Nature Portfolio journals. According to the Nature formatting guide, "Fig." is used for in-parenthesis citations, whereas the full word "Figure" is used when integrated into sentence structure (e.g., at the beginning of a sentence).
In our manuscript:

- Line 284: "Figure 3a and 3b clearly display..." — "Figure" is spelled out in full, as it begins the sentence.
- Line 247: "(Fig. 2f)" — "Fig." is correctly abbreviated, as it appears within parentheses.

To ensure consistency, we have carefully reviewed all occurrences of 'Figure' and 'Fig.' throughout the manuscript. We confirm that their usage complies with the standard formatting conventions: 'Figure' appears at the beginning of sentences, while 'Fig.' is used within parentheses.

Comment 5. Line#110: The anonymous abbreviation "vdW" must be defined.

Response: Thank you for your careful reading and for pointing out the undefined abbreviation. We

apologize for this oversight. In the revised manuscript, we have defined the abbreviation "vdW" at its first appearance as "van der Waals (vdW)". (Page 4, highlighted in yellow).

Comment 6. Typos: Line#107 'stacking phase(2H)' line#198 Fig.2; Line#207 Fig.5; 209, NbOX2(Fig…. ; linwe 217, thickness(Fig. 2a, b); line#350, Fig.4|;

Response: Thank you for your careful reading and for pointing out these formatting details. We have corrected all the spacing issues you highlighted, including: Line 107: "stacking phase(2H)" → "stacking phase (2H)"; Line 198: "Fig.2" → "Fig. 2"; Line 207: "Fig.5" → "Fig. 5" Line 209: "$NbOX_2$(Fig." → "$NbOX_2$ (Fig."; Line 217: "thickness(Fig. 2a, b)" → "thickness (Fig. 2a, b)"; Line 350: "Fig.4|" → "Fig. 4 |". In addition, we have systematically checked the entire manuscript for similar spacing inconsistencies and made corrections where needed. All changes have been highlighted in yellow.

Comment 7. Typos: Page#293, 'reported·in Ref27' line#301, '550/700nm nm'; line#308, elements--χbbb;

Response: Thank you for your careful reading. We have incorporated all the formatting corrections as suggested:

Reference format: The citation "Ref.[37]" has been revised to "Ref. 37". (page 12, highlighted in yellow)

Unit correction: The redundant unit in "550/700 nm nm" has been corrected to "550/700 nm". (page 12, highlighted in yellow)

Mathematical notation: The second-order nonlinear susceptibility expression "element--$\mathrm{\chi}^{(2)}_{\mathrm{bbb}}$" has been italicized with proper formatted as element -- $\chi^{(2)}_{bbb}$", with appropriate spacing added. In addition, all matrix elements of the entire text have been set in italics. (page 13, highlighted in yellow)

Reviewer #1 (Remarks to the Author):

The authors have provided a detailed, point-by-point response to the comments raised by Reviewer 1 and Reviewer 2. They have also submitted a revised manuscript with extensive modifications, including new experimental data (extended refractive index measurements up to 1450 nm, absorption spectra via differential transmission method, error bars on key figures), expanded tables (Table S1 with comprehensive brightness and $\chi^{(2)}$ comparisons), corrected terminology, and improved logical flow. The rebuttal letters are thorough and scientifically sound.

After careful evaluation of the authors' responses and the revised manuscript, I conclude that the authors have adequately addressed the major concerns raised by both reviewers. The remaining points are either clarified to satisfaction or represent minor formatting issues that do not compromise the scientific merit. I recommend acceptance of the manuscript in its current form.

Response: We are sincerely grateful for your careful evaluation of our revised manuscript and for your recommendation of acceptance. Thank you for your constructive comments and guidance throughout the review process.

Reviewer #2 (Remarks to the Author):

Regarding comment 1.
The authors have responded to the comments on these highly important research papers in an elaborate, insightful, and scientifically sound manner. (Nature Photonics 19 (2025): 142–148.; Nature Communications 16 (2025): 1899.; Science Advances 11 (2025): adt3710; Small 2024, 21, 2407729; Advanced Materials 36 (2024): 2400858; Advanced Quantum Technologies, e00551, 2025; https://doi.org/10.21203/rs.3.rs-6420732/v1). The reviewer strongly recommends incorporating these important papers into the reference list of the main manuscript, as they further support the scientific context and enhance the overall quality and relevance of the manuscript.

Response: Thank you very much for your valuable suggestion. We fully agree that these references further support the scientific context and enhance the overall quality of our manuscript. All recommended papers have been added to the reference list as Refs. 28, 35–39 and 45 (Page 21–22, highlighted in yellow).

Regarding comment 2.
Author clarified the comment.

Response: We appreciate the reviewer's approval of our clarification.

Regarding comment 3 and 4.
The dielectric screening effect should be carefully considered and discussed in greater detail. Furthermore, incorporating theoretical predictions or calculations would significantly strengthen the manuscript and provide deeper insight into the underlying physical behavior.

Response: We sincerely appreciate the reviewer's valuable comment on dielectric screening effect. Following this comment, we calculated the real parts and imaginary parts of dielectric constants along the *a*, *b* and *c* axis for $NbOBr_2$ and $NbOI_2$ within the framework of density functional theory (DFT) using the Vienna Ab initio Simulation Package (VASP 6.3.2). The calculated real part of the dielectric constant demonstrates that the material has an extremely strong in-plane dielectric screening effect, which is far stronger than the interfacial screening caused by sample thickness variation. This is one of the reasons why no obvious Raman peak shift occurs with changing thickness. The imaginary part of the dielectric constant characterizes the optical absorption properties (*Physical Review B,2015, 92, 245123*). Calculations demonstrate a strong absorption peak around 2.5 eV in the visible region. The photon energy of the 488 nm excitation laser used in the experiment is approximately 2.54 eV, which matches this absorption peak well. Under such circumstances, the Raman intensity is highly sensitive to the interfacial screening, leading to an irregular variation in peak intensity as the sample thickness changes (*ACS Photonics, 2025, 4731-4736*). The calculated results have been incorporated into the revised supplementary materials (**Figure S9**). In the revised manuscript, we elaborate on the irregular thickness-dependent variations in Raman peak intensities, as follows:

"*The irregular intensity evolution of Raman peaks with thickness arises from thin-film optical interference, and the anisotropic vibrational characteristics of different modes and anisotropic dielectric screening effect of the crystal, rather than structural or symmetry changes. Calculated dielectric constants verify that strong intrinsic in-plane dielectric constants give rise to robust bulk screening, which stabilizes phonon frequencies against thickness-induced interfacial dielectric perturbations. Meanwhile, the large in-plane/out-of-plane dielectric anisotropy, coupled with visible-band resonant absorption, render these out-of-plane modes susceptible to interfacial screening fluctuations and thus induce irregular intensity changes (Supplementary Fig. 9).*" (Page 8, highlighted in yellow)

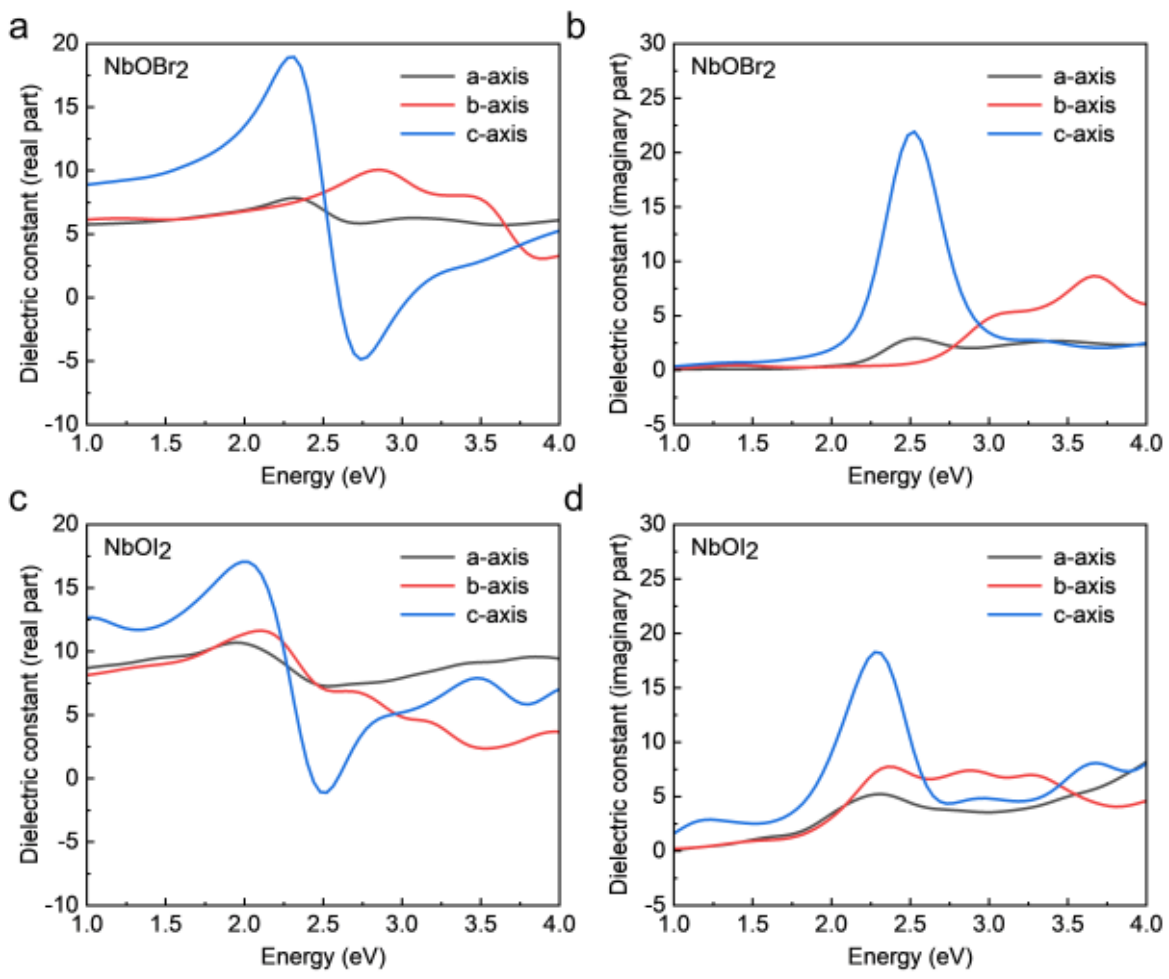

Figure S9 | Anisotropic dielectric constants of bulk $NbOBr_2$ and $NbOI_2$. (a, c) Real part and (b, d) imaginary part of dielectric constants along different crystallographic axes.

Regarding comment 5.

The authors have included the absorption spectrum of $NbOI_2$ measured using the differential transmission method, covering a broad spectral range from 300 to 1000 nm. The origin of the

absorption peak observed at 350 nm has not been adequately discussed in the manuscript. The authors should clarify the physical origin of this peak and explain how it influences the optical properties, particularly the nonlinear optical response and related photophysical behavior.

Response: Thank you very much for your valuable comment. The absorption peak at ~350 nm (3.54 eV) is assigned to high-energy excitonic transition in the polar (*b*-axis) direction, as unambiguously verified by GW-BSE calculations and ellipsometry measurements in previous work (*Nature. Photonics, 2022, 16, 644–650*). Theoretically, this excitonic resonance can substantially enhance the SHG tensor along the polar axis. However, restricted by experimental conditions, we did not investigate its influence on the second-order nonlinear susceptibility $\chi^{(2)}$. Following your advice, we have added a brief explanation in the revised manuscript as follows:

"*Apart from the above observations, $NbOI_2$ exhibits a prominent absorption peak at 350 nm. It is attributed to high-energy excitonic transitions in the polar (b-axis) direction and is expected to significantly enhance the second-order nonlinear susceptibility. Restricted by experimental conditions, we were unable to measure the SHG and SPDC photons at this wavelength. Nevertheless, we believe the ultraviolet absorption peaks of these two materials show great potential for ultraviolet lasers and visible quantum light sources.*" (Page 9, highlighted in yellow)

Regarding comment 6.
Addressed.

Response: We sincerely appreciate the reviewer's approval of our explanations.

Article No-

Title: Novel nonlinear quantum light source with van der Waals ferroelectric NbOX2 2 (X=Br, I)

In this manuscript, Duan and co-workers investigate the second-order nonlinear optical response of $NbOX_2$ and examine its intrinsic physicochemical properties. Spontaneous parametric down-conversion (SPDC) is also studied. The authors report an entangled-state concurrence of 0.90 ± 0.05 and a theoretical fidelity of 0.94 ± 0.05. However, the reported brightness of the $NbOBr_2$ crystal (1.5 Hz/mW) is significantly low compared to recent reports on the same and other van der Waals materials. Consequently, the claim that $NbOBr_2$ represents a *novel* nonlinear quantum light source is not justified, as several studies have already reported s results using this material as well as other van der Waals systems [Nature Photonics 19 (2025): 142–148.; Nature Communications 16 (2025): 1899.; Science Advances 11 (2025): adt3710; *Small* 2024, 21, 2407729 ; Advanced Materials 36 (2024): 2400858; Advanced Quantum Technologies, e00551, 2025; *Nanophotonics*, vol. 14, no. 27, 2025, pp. 5337-5345; Nature Communications 14 (2023): 5911; " Nature 613 (2023): 53–59; Applied Physics Letters 125 (2024): 151902]. The quantum characterization presented in this work is insufficient. Quantum state tomography is a critical requirement for establishing an entangled quantum light source; however, the manuscript lacks rigorous, detailed, and convincing experimental evidence. The absence of novelty, comprehensive and statistically significant results makes not to support publication in *Nature Communications*, particularly given the journal's broad and multidisciplinary scientific audience.

The paper is scientifically weak in terms of novelty and broader impact, and it does not meet the standards required for publication in *Nature Communications*.

The article should be published in lower impact journals.

Recommendation: The article is not appropriate for publication in *Nature Communications*. Detailed comments are provided below.

Note. The manuscript contains numerous typographical errors and requires substantial revision. Overall, it appears to have been prepared without sufficient care and attention to detail.

Comments

1. The authors report an entangled-state concurrence of 0.90 and a theoretical fidelity of 0.94 ± 0.05. However, the reported brightness of the $NbOBr_2$ crystal (1.5 Hz/mW) is significantly low compared to recent reports using the same material as well as other van der Waals (vdW) materials (for eg. Nature Photonics 19 (2025): 142–148.: NbOCl2,

31KHz/mW.mm" Nature Communications 16 (2025): 1899: r-BN, 8667 Hz/mW.mm,). Despite this, the authors claim the system to be a *novel nonlinear quantum light source*, which is not accurate. There is no comparisons of results and scientific reasons of lower brightness. Several studies have already reported similar & extremely high brigntness or generation rate with Fidelity and CAR results using $NbOBr_2$ and other vdW materials, undermining the claimed novelty. [Nature Photonics 19 (2025): 142–148.; Nature Communications 16 (2025): 1899.; Science Advances 11 (2025): adt3710; *Small* 2024, 21, 2407729 ; Advanced Materials 36 (2024): 2400858; Advanced Quantum Technologies, e00551, 2025; https://doi.org/10.21203/rs.3.rs-6420732/v1]

2. This is an incorrect statement!!! 'As the first reported 2D material-based nonlinear quantum source, ferroelectric van der Waals materials NbOCl2, with distinctive distorted structure exhibits several notable properties' The first report was published on 2D transition metal dichalcogenides.
3. Unlike two-dimensional (2D) materials, the Raman peak positions do not exhibit any noticeable shift; however, the intensities of the two modes (P3 and P4) vary irregularly. The observation is unclear.
4. The Raman peak positions are signatures of the crystal vibrational modes and are critical for defining the existence and preservation of symmetry. However, the peak energies in the spectra have been overlooked.
5. The authors claim that $NbOI_2$ exhibits two main peaks at approximately 550 nm and 700 nm. However, the reviewer believes that the quality of the absorption spectra is extremely poor. If differential reflection measurements cannot be introduced, the resulting conclusions remain ambiguous. Moreover, both crystals exhibit a strong and well-defined peak around 350 nm, which appears to have been intentionally skipped or overlooked.
6. Line#321/322 'is negligible for both materials (see more details in 322 Supplementary Section 4). This appears to be merely superficial, as no specific details are provided to address this comment.
7. The author uses the term **"**coherent length**"**, whereas the scientifically accepted term used by the research community is **"**coherence length**."**
8. Why did the authors not choose a $NbOBr_2$ thickness corresponding to a shorter coherence length? Initially, in the Introduction, this was presented as an advantage of two-dimensional materials. However, the interpretation of the SHG and SPDC results appears to be contradictory.
9. 'the calculated lower efficiencies of NbOI2 392 comparing to NbOBr2 confirm the aforementioned influence of longitudinal phase 393 mismatch and index contrast.' The statement is overly general and merely reiterates advantages of two-dimensional materials over conventional bulk crystals by citing previous studies. No new fundamental insights or original scientific contributions are presented here.

10. The term used in incorrect 'coincidence-accident-rate' is basically 'coincidence to accidental ratio'.
11. Author reported that 'NbOBr2 crystal achieves a brightness of 1.5 Hz/mw at 914nm, which is comparable to the performance of NbOCl2 crystal at 808nm', which is pretty low compared to the performance of other reported results same NbOCl2 and other 2D. [Nature Photonics 19 (2025): 142–148.; Nature Communications 16 (2025): 1899.; Science Advances 11 (2025): adt3710.]

Minor comments,

1. The manuscript contains numerous typographical errors and requires substantial attention. Overall, it appears to have been written carelessly.
2. 'photonical structures' is incorrect scientific term.
3. 'AlN microring', 'LN microdisk' abbreviations without proper scientific pronounced name
4. Inconsistence of the Figure abbreviation in the text. For Eg Line# 284 Figure 3a and 3b and line#247 (Fig. 2f).
5. Line#110: The anonymous abbreviation "vdW" must be defined.
6. Typos: Line#107 'stacking phase(2H)' line#198 Fig.2; Line#207 Fig.5; 209, NbOX2(Fig…. ; linwe 217, thickness(Fig. 2a, b); line#350, Fig.4|;
7. Typos: Page#293, 'reported·in Ref27' line#301, '550/700nm nm'; line#308, elements--χbbb;
